# One‐Pot Biphasic Photocatalytic CO_2_‐to‐Carbonylation Cascades

**DOI:** 10.1002/anie.5220236

**Published:** 2026-09-25

**Authors:** Sampurna Mitra, David M. Vahey, Shannon A. Bonke, Erwin Reisner

**Affiliations:** ^1^ Yusuf Hamied Department of Chemistry University of Cambridge Cambridge UK

**Keywords:** biphasic catalysis, carbon dioxide fixation, carbonylation, photocatalysis

## Abstract

Photosynthesis compartmentalizes reactions across the thylakoid membrane in vivo to upcycle CO_2_ into complex chemicals that are fundamental to life. Herein, we demonstrate a step toward achieving a synthetic equivalent to the chloroplast with a compartmentalized cascade reaction in vitro that enables light driven CO_2_ utilization for organic synthesis in one‐pot. This bioinspired compartmentalization enables the photocatalytic reduction of aqueous CO_2_ by water‐soluble catalysts and dyes to produce CO, which then moves into the more buoyant organic layer and is consumed in aminocarbonylation or carbonylative variants of Sonogashira and Suzuki couplings, including the synthesis of the pharmaceutical itopride. The reaction compartmentalization significantly increases the biomimicry of CO_2_ utilization by enabling the light‐driven generation and utilization of CO from CO_2_ for chemical synthesis in the same pot compared to previous two‐pot approaches. The fundamental platform and design criteria described herein can enable future reactions to utilize building blocks as abundant as CO_2_ to generate complex molecules through bioinspired pathways.

## Graphical abstract

Bioinspired biphasic compartmentalization enables one‐pot cascades where photocatalytic CO_2_ reduction generates CO for carbonylative cross‐couplings to synthesize complex organic products, including a pharmaceutical.

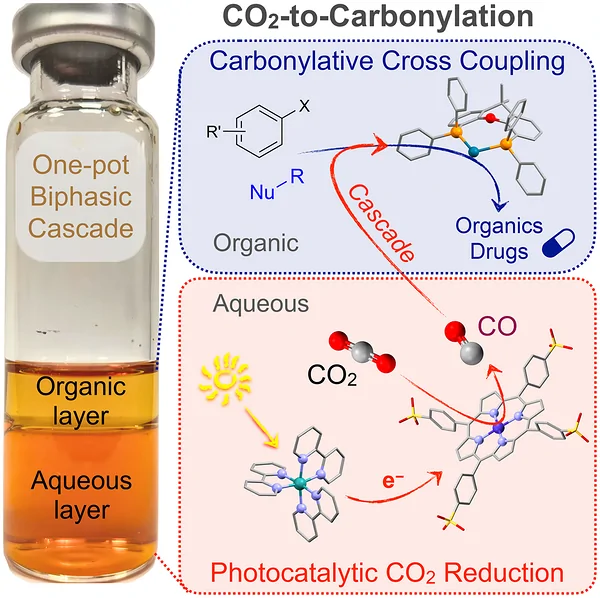

## 1 Introduction

Petrochemical consumption accounts for over 75% of global anthropogenic CO_2_‐equivalent emissions [1], but the ability to use CO_2_ as a synthetic building block for complex molecules, outside of biology, remains an unresolved challenge [2]. Unlocking biomimetic chemistry to fix CO_2_ into organic chemicals provides an attractive path toward sustainable and economically viable carbon management. Chloroplasts in photosynthetic systems use sunlight to produce complex organic chemicals from CO_2_ and H_2_O [3, 4]. These transformations are achieved through a cascade of reactions, with key steps compartmentalized by phase boundaries to create ideal microenvironments; that is, the solar‐powered generation of reducing equivalents (light reaction) on the lumen side of the membrane, which are then used, on the other side of the membrane, in the stroma for the Calvin cycle (dark reaction) that forms complex molecules from CO_2_ [2, 3, 4, 5, 6]. Thus, nature provides a blueprint for solar‐powered compartmentalized reaction cascades that produce bioactive molecules from the simplest building blocks.

State‐of‐the‐art synthetic chemistry enables the selective light‐driven reduction of CO_2_ to CO using dyes paired with molecular catalysts such as metal porphyrins [7, 8, 9, 10, 11, 12], but the selective one‐pot conversion of CO_2_ to complex and valuable multi‐carbon products remains a challenge [13, 14, 15, 16, 17, 18, 19]. To this end, the formation of CO_2_‐derived CO provides a handle to construct more complex organic products [14, 20, 21]. The in situ generation of CO would also render CO_2_ as a CO surrogate thus avoiding toxic gas handling. Carbonylative cross‐coupling reactions such as aminocarbonylation pioneered by Heck [22], or the carbonylative Sonogashira [23, 24] and Suzuki [25, 26, 27] reactions use CO and enable new carbon‐carbon and carbon‐heteroatom bonds for the synthesis of pharmaceutically relevant molecules in organic solvents [28, 29, 30]. However, the typical environments for these reactions are quite different from the reductive aqueous environments that have been a focus for CO_2_ reduction work [31, 32, 33, 34, 35].

The different reaction environments required for photocatalytic CO_2_ reduction and carbonylation have thus far resulted in the design of synthetic systems with physically separate reaction chambers with a shared headspace (two‐pot reactions). This approach follows the practical industrial‐chemical model of ‘consecutive reactions’, or ‘telescoped reactions’, by generating CO in one‐pot and then using it for a carbonylation reaction in a second separate pot. A pioneering example of a telescoped reaction system utilizes a CO_2_‐to‐CO electro‐reduction chamber where the headspace is connected to a carbonylation chamber containing organic solvent [36, 37]. Subsequent work has shown photochemical CO_2_‐to‐CO conversion in one chamber with carbonylation chemistry in the second chamber [38, 39, 40, 41, 42, 43]. These works establish the use of CO_2_ reduction reactions for carbonylation chemistry, but their limitation from a biomimetic perspective is that the two reaction environments are physically separated by a gas phase (two‐pots). In contrast, biology compartmentalizes reactions while maintaining liquid‐liquid contact (one‐pot); for example with the lipid phase boundaries of the thylakoid membrane [2, 3, 4, 5, 6], or the carbon monoxide dehydrogenase/acetyl‐CoA synthase (CODH‐ACS) cluster that generates CO and channels it to the carbonylation active site where a new C─C bond is formed [44]. Achieving this level of reaction compartmentalization (chemical separation), and the consequent control over reaction pathways and substrate channeling, remains a major challenge for synthetic chemistry [45].

An elegant one‐pot reaction cascade using CO_2_ electroreduction to CO followed by a coupling reaction has been shown [46], but this required DMF as a solvent, was not photochemical, and the substrate scope was limited to symmetrical ketones, thus limiting its biomimicry. Formal cascade reactions, where intermediates are made and consumed at the same time in the same pot [47], are unknown for photocatalytic CO_2_‐to‐CO‐to‐carbonylation chemistry. Replacing physical separation (two‐pots) with chemical separation (one‐pot) is therefore a step toward compartmentalized cascades as used by biology to build molecular complexity from CO_2_.

In this study, we use a biphasic reaction compartmentalization strategy to enable photocatalytic CO_2_ reduction and carbonylation in one‐pot. This bioinspired approach avoids the gas phase separation of the two reaction pots by exploiting the different reaction environments required for CO_2_ reduction and carbonylation. The system enables photocatalytic CO_2_ reduction to CO in an aqueous layer and Pd cross‐coupling carbonylation reactions to complex products in a hydrophobic organic toluene layer (Scheme 1). Keeping the two reactions in liquid‐liquid contact with CO preferentially moving from one reaction site to the next mimics the CODH‐ACS enzyme cluster that pairs CO_2_ reduction with carbonylation [44]. The key design principles to establish these reaction cascades are first explored with water‐soluble cobalt porphyrin electrocatalysts that are selective for aqueous CO_2_ to CO conversion when used together with either ruthenium(II) tris(bipyridine) or Cu(I) diamine‐diphosphine dyes and ascorbic acid as an electron donor. These photocatalytic systems enable the production of CO, which is then utilized in situ by a Pd‐Xantphos cross‐coupling catalyst in the organic layer to carbonylate aryl iodides with primary amines at ambient temperatures. The substrate scope is explored before the organic reaction scope is expanded to include carbonylative Sonogashira and Suzuki couplings, as well as the synthesis of the drug itopride. The conceptual versatility and compartmentalization of the system is shown by the ability to replace both the CO_2_ reduction photocatalysis and CO utilization reactions, showing valuable generality, including deployment of a precious‐metal‐free light reaction. This work establishes a photocatalytic cascade reaction for direct utilization of CO_2_ as a carbon source for chemical synthesis, enabled by bioinspired reaction compartmentalization.

**SCHEME 1:**
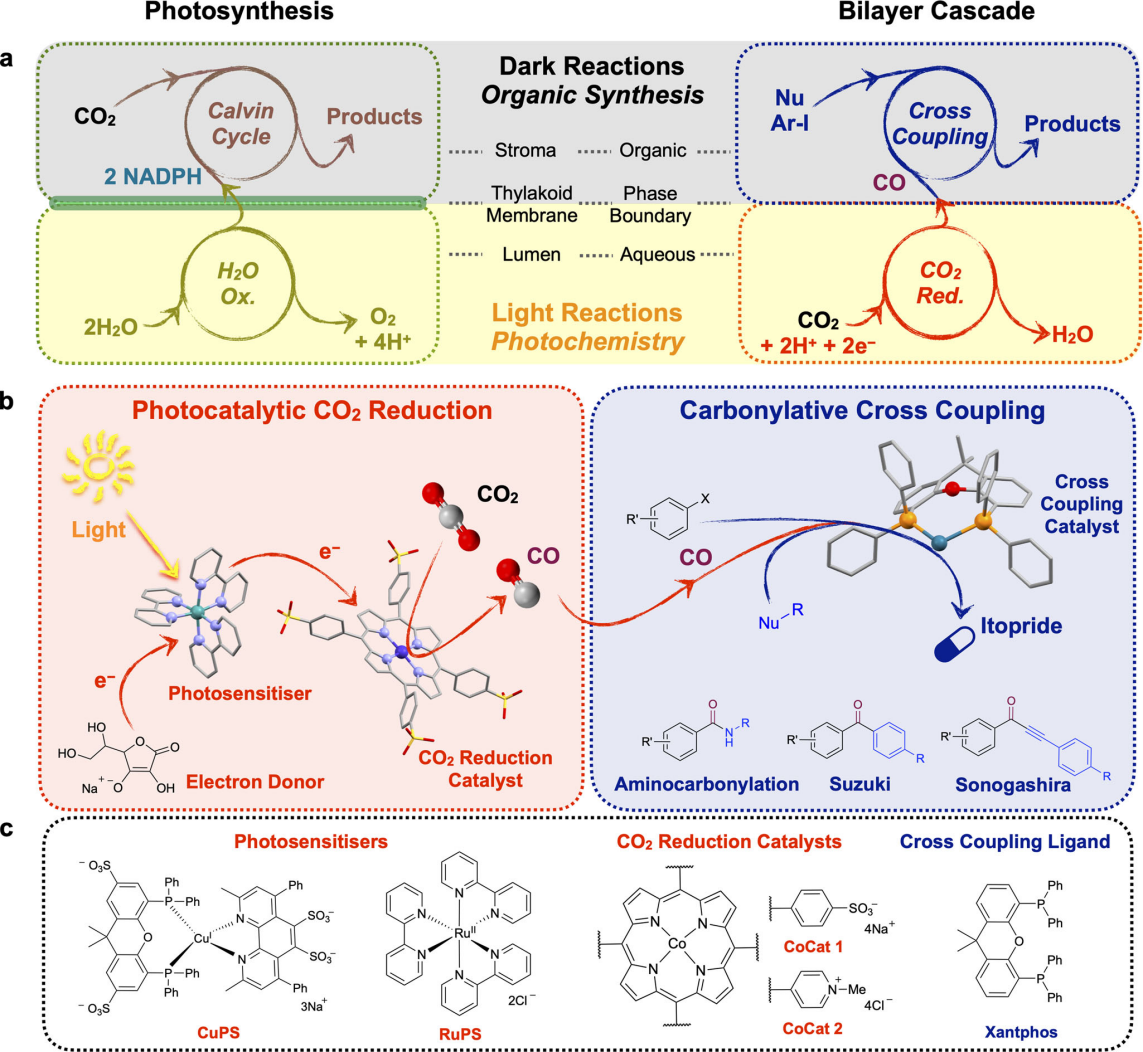
Schematic for (a) comparison of photosynthesis with the bilayer cascade highlighting the separation of the light (photochemistry) and dark (synthesis) reactions by a phase boundary in both cases, (b) photocatalytic CO_2_ reduction in the aqueous layer in the presence of a photosensitizer and CO_2_ reduction catalyst (RuPS and CoCat1 respectively depicted) and carbonylative cross‐coupling initiated by cross coupling catalyst ([Pd/Xantphos]) in the organic layer with CO moving from aqueous to organic to enable the cascade and (c) structures of photosensitizers, catalysts and cross coupling ligand. Nu = nucleophile, X = halide.

## 2 Results and Discussion

### 2.1 Photocatalytic CO_2_‐to‐CO in Aqueous Layer

To establish a cascade reaction utilizing the in situ generated CO from photocatalytic CO_2_ reduction, specific performance criteria were first identified; namely, (i) CO_2_ to CO reduction must proceed with a high rate and selectivity to generate a sufficiently high concentration of CO; and (ii) the photocatalytic components need to be preferentially soluble and functional in water rather than organic solvent to enable biphasic compartmentalization. Cobalt porphyrins are highly active and selective CO_2_‐to‐CO molecular catalysts, and can be made water soluble through substituent functionalization to create a charged periphery [8, 9, 48]. This includes anionic tetra‐sulfonato‐phenyl porphyrins and cationic tetra‐methyl‐pyridinio porphyrins, which can be selected to have a complementary electrostatic attraction to a water‐soluble metal complex photosensitizer [8]. Suitable visible‐light photosensitizers to deliver electrons and drive the CO_2_ reduction reaction are ruthenium(II)tris(2,2’‐bipyridine) dichloride (RuPS; Scheme 1) and Na_4_[Cu(5,6‐disulfonato‐bathocuproine)(2,7‐bissulfonato‐xantphos)](BF_4_) (CuPS; Scheme 1), which have each been previously shown to function with cobalt porphyrins [8, 9, 10, 11].

Initial photocatalytic studies utilized Na_4_[Co tetra(sulfonatophenyl)porphyrin] (CoCat1 at 10 µM; Scheme 1) and an excess of RuPS (500 µM) in a CO_2_ saturated bicarbonate solution (100 mM, pH 6.8), also containing sodium ascorbate (NaHAsc; 100 mM) as an electron donor (reductive quencher of RuPS) [8, 9, 10, 11]. The solution was illuminated from below with a blue LED (447 nm, 2.3 W) at 25°C with orbital convection and the headspace gas sampled by GC.

Upon illumination, the RuPS/CoCat1 photocatalytic system displayed a linear increase of CO in the headspace, producing 34 ± 2 µmol after 4 h (8.5 µmol_CO_ h^−1^) with a catalyst‐based turnover number (TON_CO_) and frequency (TOF_CO_) of 561 ± 28 and 140 ± 7 h^−1^, respectively (Figure 1a). The CO yield then reached its maximum of 74 ± 4 µmol of CO after 24 h irradiation with a TON_CO_ of 1225 ± 61 and apparent TOF_CO_ of 51 ± 3 h^−1^, with the selectivity for CO over H_2_ stable at 83 ± 4% throughout the experiment. Allowing the reaction to proceed for 48 h did not result in further CO formation, which is attributed to degradation of the photocatalytic system [8, 11, 49]. Exclusion controls showed that no CO formed under dark conditions or when the photosensitizer or electron donor was excluded (photocatalytic system optimization is discussed in Note S1; Figures S1, S2, and Table S1).

**FIGURE 1:**
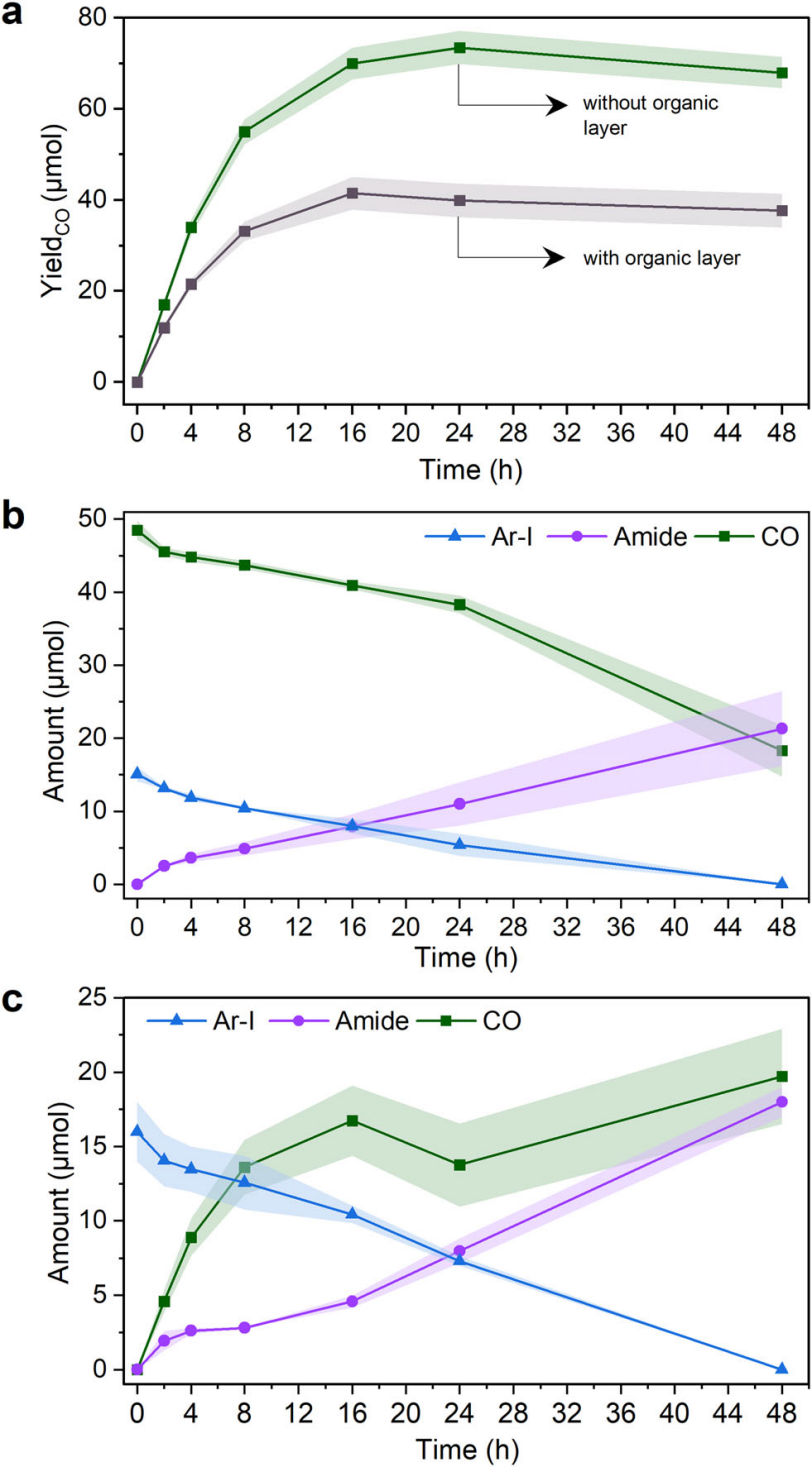
Kinetic traces for (a) aqueous photocatalytic generation of CO from CO_2_: CoCat1 (10 µM), RuPS (500 µM), NaHAsc (100 mM), NaHCO_3_ (100 mM) in H_2_O (CO_2_ sat., pH 6.8, 6 mL); (b) aminocarbonylation bilayer with injected CO in the dark: organic layer: iodoanisole (**1**, 20 µmol, 1 equiv.), octylamine (**2**, 100 µmol, 5 equiv.), Pd(OAc)_2_ (5 mol%), Xantphos (10 mol%) in toluene (N_2_ sat., 2 mL); aqueous layer_:_ NaHCO_3_ (100 mM) in H_2_O (CO_2_ sat., pH 6.8, 6 mL); (c) biphasic photocatalytically induced reaction cascade layering the organic solution from B on the aqueous solution from A with no exogenous CO added. Illumination with blue LED (447 nm, 2.3 W), 25°C, 250 rpm orbital shaking. Quantification by GC (gases) and GC‐MS (liquids). Reactions in duplicate, errors plotted as ± 1 standard deviation with a minimum value of 5%, 48 h data point from different reaction batch. Tabulated values in SI.

Layering toluene on top of the aqueous layer resulted in CO dissolution within the organic phase [50] and lowered the detected headspace CO by ∼45% across the kinetic series (74 ± 4 vs 40 ± 4 µmol after 24 h), with linearity of CO production before saturation remaining unchanged (Figure 1a and Table S1; CO dissolution in the organic layer is discussed in Note S2, Table S2). This results in an initially linear observed CO formation rate of 5.5 µmol_CO_ h^−1^ in the headspace before reaching a yield of 40 ± 4 µmol after 24 h, with no further increase to 48 h.

The RuPS was then replaced by the precious‐metal‐free photosensitizer, CuPS, which is a Cu(I) diamine‐diphosphine complex with sulfonate functional groups to enable water solubility (Scheme 1) [9, 51, 52]. The concentrations were all unchanged from the optimized conditions, but the wavelength was changed to 405 nm (2.3 W LED) to more closely match the photosensitizer absorption band (λ_max_ 390 nm; Figure S2).

The anionic CuPS photosensitizer was coupled with cationic [Co tetra(N‐methyl)pyridino porphyrin]Cl_4_ (CoCat2; Scheme 1) as the CO_2_ reduction catalyst to pair the peripheral electrostatics of the complexes and consistent with literature precedent [9]. This CuPS/CoCat2 photosystem produced 105 ± 5 µmol of CO in 24 h with 81 ± 4% selectivity over H_2_ for a TON_CO_ and TOF_CO_ of 1742 ± 87 and 73 ± 4 h^−1^, respectively (Figure S3 and Table S3). Consistent with literature, using CoCat1 with CuPS results in a lower CO yield (35 ± 2 µmol), while the RuPS/CoCat2 combination yields 80% less CO than the RuPS/CoCat1 system used herein (15 ± 5 vs 74 ± 4 µmol CO) [9]. Thus, both the RuPS/CoCat1 and the CuPS/CoCat2 systems reduce CO_2_ to CO with high yield and selectivity rendering them suitable for initiating CO_2_ reaction cascades.

### 2.2 Aminocarbonylation in the Organic Layer

Aminocarbonylation is a fundamental and established cross‐coupling reaction that employs catalytic palladium catalysts to couple an aryl halide with CO and an amine to form an aryl amide [53]. This reaction proceeds at ambient temperature and pressure when using nucleophilic amines in water‐immiscible solvents [53], which enables bilayer formation when added to an aqueous photocatalytic CO_2_ reduction system. Therefore, aminocarbonylation was evaluated for performance in an aqueous‐organic biphasic solvent system prior to demonstrating the integrated photocatalytic biphasic cascade reaction.

The model aminocarbonylation reaction employed reagents that partition into the toluene layer of an immiscible aqueous‐organic mixture to compartmentalize them in the organic layer; specifically, 4‐iodoanisole (**1**, 0.02 mmol, 1 equiv.) and octylamine (**2**, 0.10 mmol, 5 equiv.). The reaction was catalyzed by Pd (5 mol%) with Xantphos ligand (10 mol%; Scheme 1) at 25°C for 24 h (these standard reaction conditions were optimized for the full photocatalytic system, vide infra). In toluene with 2.5 equiv. of CO delivered exogenously by syringe, 4‐methoxy‐N‐octylbenzamide (**3**), was formed in 82% yield (Table 1, Entry 1). To probe the aminocarbonylation reaction compatibility with the biphasic configuration, the reaction was repeated after the organic toluene solution was added on top of a CO_2_ saturated bicarbonate aqueous layer (1:3 toluene:water; 100 mM NaHCO_3_, pH 6.8), with 2.5 equiv. of CO still delivered exogenously. This configuration enabled formation of benzamide **3** in 73% yield (Table 1, Entry 2).

**TABLE 1:** Model reaction for aminocarbonylation and system development for monophasic and biphasic systems using exogenous CO, and for the biphasic system with photogenerated CO reduced from CO_2_ ^a^.

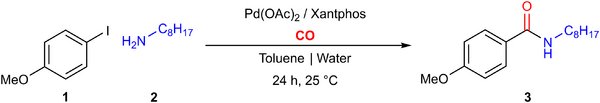									
Entry	System	2 (equiv.)	External CO (equiv.)	Photocatalytically produced CO (equiv.)	Pd(OAc)_2_ (mol %)	Xantphos (mol%)	Solvent	Misc	Yield 3 (%)
**1**	Monophase	5	2.5	—	5	10	Toluene	—	82
**2**	Biphase	5	2.5	—	5	10	Toluene | Water	—	73
**3**	Photocatalytic Biphase	5	—	hν (∼2.5)	5	10	Toluene | Water	—	74
**4**	Photocatalytic Biphase ^b^	5	—	hν (∼2.5)	5	10	Toluene | Water	CuPS	71
**5**	Telescoped Two‐Compartment^c^	5	—	hν (∼2.5)	5	10	Toluene and Water Vials	—	50
**6**	Photocatalytic Biphase^d^	5	—	hν (∼2.5)	5	10	Toluene | Water	Solar Light	64
**7**	Photocatalytic Biphase	5	—	hν (∼2.5)	5	5	Toluene | Water	—	61
**8**	Photocatalytic Biphase	3	—	hν (∼2.5)	5	10	Toluene | Water	—	22
**9**	Photocatalytic Biphase	5	—	hν	5	10	Toluene | Water	No [Ru]	n.d.
**10**	Photocatalytic Biphase	5	—	hν	5	10	Toluene | Water	No CO_2_	n.d.
**11**	Photocatalytic Biphase	5	—	—	5	10	Toluene | Water	No light	n.d.
**12**	Photocatalytic Biphase	5	—	hν (∼2.5)	0	10	Toluene | Water	No [Pd]	n.d.

^a^ Organic layer: iodoanisole (**1**, 20 µmol, 1 equiv.), octylamine (**2**), Pd(OAc)_2_ and Xantphos in toluene (N_2_ sat., 2 mL). Biphase tests (Entry 2)—the organic layer on top of an aqueous layer: NaHCO_3_ (100 mM) in H_2_O (CO_2_ sat., pH 6.8, 6 mL); Photocatalytic biphase tests (Entries 3,5‐12) used aqueous photocatalytic generation of CO from CO_2_ with organic layer on solution of CoCat1 (10 µM), RuPS (500 µM), NaHAsc (100 mM), NaHCO_3_ (100 mM) in H_2_O (CO_2_ sat., pH 6.8, 6 mL). LED illumination (447 nm, 2.3 W), 25°C, 250 rpm orbital shaking. All reactions 24 h. Yields determined by ^1^H NMR using 1,3,5‐trimethoxybenzene as an internal standard, n.d. = none detected. Yields have a 10% batch‐to‐batch error that is representative of all measurements.

^b^ CoCat2 (10 µM) and CuPS (500 µM) used in place of CoCat1 and RuPS with LED illumination (405 nm, 2.3 W)

^c^ headspace of a vial containing the aqueous layer for CO_2_ reduction to CO connected to a second vial that contained the organic layer for carbonylation.

^d^ illumination with simulated solar light (AM 1.5G, 100 mW cm^−2^).

The kinetics of the biphasic reaction were tracked by headspace GC analysis and GC‐MS sampling of the toluene phase to quantify CO and aryl iodide conversion as well as benzamide yield (Figure 1b and Table S4). At the start of the reaction (0 h), the 48 ± 1 µmol of injected CO was detected within the headspace while 15 ± 1 µmol of aryl iodide **1** was within the organic layer and no benzamide **3** was detected. The kinetic tracking revealed that the formation of benzamide **3** was linear from 2–48 h (r^2^ = 0.99, 0.4 µmol h^−1^). The reaction reaches completion at 48 h (21 ± 5 µmol of **3**), concurrent with the expected linear decay of aryl iodide until it is fully consumed. The yield of benzamide **3** at 24 h based on GC‐MS quantification is 73%, which is consistent with the batch reaction (Figure 1b and Table S4).

The linearity of the carbonylation reaction over time, that is, not showing a rate change as CO was consumed, indicates that the organic reaction rate should not be perturbed by the photocatalytic CO generation rate (cf. Figure 1b,a). The reaction rate of CO generation from CO_2_ compared to CO consumption through carbonylation indicates that carbonylation will be rate limiting at this reaction temperature with an approximately 10‐fold difference in rates (5.5 vs 0.4 µmol h^−1^). Overall, these results confirm the suitability of incorporating aminocarbonylation as part of a cascade, as the reaction is (i) water tolerant and functional in a biphasic configuration, and (ii) the rate of photocatalytic CO generation is sufficient to provide stoichiometric or greater amounts of CO for the carbonylation.

### 2.3 One‐Pot CO_2_ Reduction‐to‐Aminocarbonylation Cascade

The photocatalytic and organic environments were then combined to enable the compartmentalized cascade reaction where CO_2_ is reduced to CO in the aqueous layer, before the in situ generated CO is incorporated into the organic product via carbonylation in the organic layer to form benzamide **3** (reaction scheme in Table 1). To prepare the biphasic configuration, the organic layer containing 4‐iodoanisole (**1**), octylamine (**2**), Pd(OAc)_2_ and Xantphos in toluene (N_2_‐sat., 2 mL) was layered onto the optimized aqueous layer containing catalyst (10 µM), photosensitizer (500 µM), NaHAsc (100 mM) and NaHCO_3_ (100 mM) in water (CO_2_ sat., pH 6.8, 6 mL), under a CO_2_ atmosphere with no CO added (Figure S4). Either the RuPS/CoCat1 or CuPS/CoCat2 CO_2_ reduction photosystems were used, with the biphasic solutions illuminated from below (447 nm for RuPS and 405 nm for CuPS, 2.3 W LED) at 25°C with orbital convection (250 rpm) for 24 h. The organic layer was first optimized to enable the reaction cascade, with the yield of organic product determined by quantitative ^1^H NMR spectroscopy.

Starting with the RuPS/CoCat1 CO_2_ reduction photosystem and typical aminocarbonylation conditions in the organic layer [53], specifically, 4‐iodoanisole (**1**, 0.02 mmol, 1 equiv.), octylamine (**2**, 0.03 mmol, 1.5 equiv.), base (triethylenediamine = DABCO; 0.06 mmol, 3 equiv.), Pd(OAc)_2_ (2 mol%) and Xantphos (2 mol%), only 5% yield of benzamide **3** was obtained in the photocatalytic cascade (Table S5). In contrast, we found that these typical conditions yielded 91% benzamide **3** if only anhydrous toluene (without any water layer) and injected CO was used [53], indicating that a specific optimization would be required to enable the envisioned biphasic cascade. Thus, optimization of the carbonylation reaction was performed with the full photocatalytic cascade utilizing CO_2_ as the CO source. Starting with the role of the base and amine, the carbonylation reaction requires deprotonation of the amine upon coupling, but the presence of base did not affect the reaction yields (Table S5), which was attributed to the extraction of the base into the aqueous layer and the buffering effect of aqueous bicarbonate (0.6 mmol NaHCO_3_ at pH 6.8). Adding excess octylamine so that it functions as both a reagent and base only resulted in a marginal yield increase (from 5% up to 9% at 5 equiv.; Table S5), indicating the limitation was the catalyst. Running the cascade reaction with increased Pd(OAc)_2_ (5 mol%) and Xantphos (5 mol%), and 5 equiv. of amine, enabled good yields of benzamide **3** in the full cascade (61%, Table 1, Entry 7), while further increasing the Xantphos loading to 10 mol% further improved the yield to 74% (Table 1, Entry 3). When using these higher loadings of Pd(OAc)_2_ (5 mol%) and Xantphos (10 mol%), decreasing the amine loading from 5 to 3 equiv. decreases the yield from 74% to 22% (Table 1, Entry 8), corroborating the rationale detailed above. The potential instability of Xantphos or limited activation of the Pd(II) by Xantphos in ‘wet’ organic conditions was postulated to be necessitating the higher cross‐coupling ligand loading, but a screening of alternative Pd ligands revealed none that displayed significant activity (Table S6). Overall, suitable conditions were established to enable the cascade and the reaction optimization exemplifies the importance of pairing and optimizing the reactions for cascade conditions as demonstrated herein.

Thus, the standard conditions were established to be 4‐iodoanisole (**1**, 0.02 mmol, 1 equiv.), octylamine (**2**, 0.10 mmol, 5 equiv.), Pd(OAc)_2_ (5 mol%) and Xantphos (10 mol%) in toluene (N_2_‐sat., 2 mL), layered onto water (CO_2_ sat., pH 6.8, 6 mL) containing catalyst (10 µM), photosensitizer (500 µM), NaHAsc (100 mM) and NaHCO_3_ (100 mM) under a CO_2_ atmosphere. Using the RuPS/CoCat1 photosystem resulted in a 74 ± 10% yield of benzamide **3** (Table 1, Entry 3; the 10% error is a batch‐to‐batch error and representative of all measurements), while using the CuPS/CoCat2 photosystem resulted in a 71% yield of benzamide **3** (Table 1, Entry 4). These consistent yields with different CO generating photosystems support that the light and dark reactions are compartmentalized with transfer of CO across the phase boundary. It is also notable that there was no loss of benzamide yield when comparing the cascade reaction to the biphasic configuration with injected CO (71%–74% vs 73%, Table 1, Entry 2 vs 3 and 4). Therefore, the bilayer configuration when using in situ generated CO results in no losses compared to adding exogenous CO in excess. Exclusion controls show that there is no product formed without light, CO_2_, photosensitizer or the [Pd] catalyst (Table 1, Entries 9‐12). Taken together, these results support that the cascade is compartmentalized and proceeds from CO_2_ reduction to CO, through to carbonylation to form benzamide **3**.

The kinetics of the cascade reaction using the RuPS/CoCat1 photosystem were then studied with the CO and dissolved organics tracked over time by GC and GC‐MS, respectively (Figure 1c and Table S7). This kinetic study reveals that the CO in the headspace grows linearly for the first 4 h (r^2^ = 0.99, 2.2 µmol h^−1^), which is consistent with the linearity observed for the purely aqueous and toluene layered photocatalytic systems (Figure 1c vs 1a). However, the rate of CO formation is decreased 60% compared to the latter system, which is attributed to a combination of partial CO_2_ capture as carbamate by the amine, CO_2_ dissolution into toluene and a contribution from interference of the light path by immiscible solvent mixing during the reaction convection/shaking (see Note S2). Regardless, the yield of benzamide **3** increases linearly from 0–48 h (r^2^ = 0.99, 0.4 µmol h^−1^; Figure 1c), with the rate of benzamide formation being identical to that observed when excess CO is injected at the start of the reaction (cf. Figure 1b,c), which indicates the cascade reaction is not CO limited. The reaction proceeds to completion in 48 h with full conversion of 16 ± 2 µmol iodoanisole into 18 ± 1 µmol of benzamide **3**. The photocatalytic system remains in the aqueous layer and visibly shows degradation post reaction (RuPS and CoCat1 bleaching; see Note S3 and Figure S4). These photochemical processes have not prevented the organic reaction, which supports compartmentalization. A practical benefit is that the organic products can be collected by decanting the organic layer, with the preferential organic solubility of the product also facilitating extraction, to minimize issues associated with purification from water.

We next compared our compartmentalized cascade reaction, where the CO intermediate is produced and consumed in one‐pot, to a telescoped reaction where the CO from one‐pot is used for carbonylation in a second pot as previously reported [36, 37, 38, 39, 40, 41, 42, 43]. The latter two‐pot setup was assembled by connecting the headspace of a vial containing the RuPS/CoCat1 aqueous layer for CO_2_ reduction to CO, to a second vial that contained the organic layer for carbonylation (all solution volumes unchanged; similar headspace volumes). This telescoped configuration resulted in 50% yield of benzamide **3** (Table 1, Entry 5), whereas our one‐pot reaction cascade provided a 1.5‐fold higher yield of 74%. This improvement may be attributed to lower mass transport limitations, that is better extraction of CO resulting in a higher local concentration in the organic layer and thereby more effective utilization of CO.

The cascade reaction also proceeds under simulated solar light illumination (AM 1.5G, 100 mW cm^−2^, side illumination) with 64% yield after 24 h using the RuPS/CoCat1 photosystem (Table 1, Entry 6), which is within error of tests using the blue LEDs (74 ± 10%; with 447 nm, bottom illumination). Taken together, these results show that the cascade demonstrated herein enables solar‐powered carbonylation reactions utilizing simulated sunlight and CO_2_ as a carbon source for fine chemical production in compartmentalized cascades.

### 2.4 Isotopic Labelling

Isotopic labelling experiments using ^13^CO_2_ were then conducted, using the standard conditions with RuPS/CoCat1 and 447 nm LED illumination, to track the carbon from CO_2_ to CO, and then into the carbonylated product. IR spectroscopy of the headspace gas from the photocatalytic system shows that ^13^CO is produced when using ^13^CO_2_, with only a trace amount of ^12^CO also detected that was attributed to ascorbate decomposition (Figure 2a). The formation of ^13^CO was confirmed by the CO stretch shifting from 2143 to 2096 cm^−1^ when ^13^CO_2_ was used, which confirms that the observed CO originates from the reduction of CO_2_ [10, 11, 54].

**FIGURE 2:**
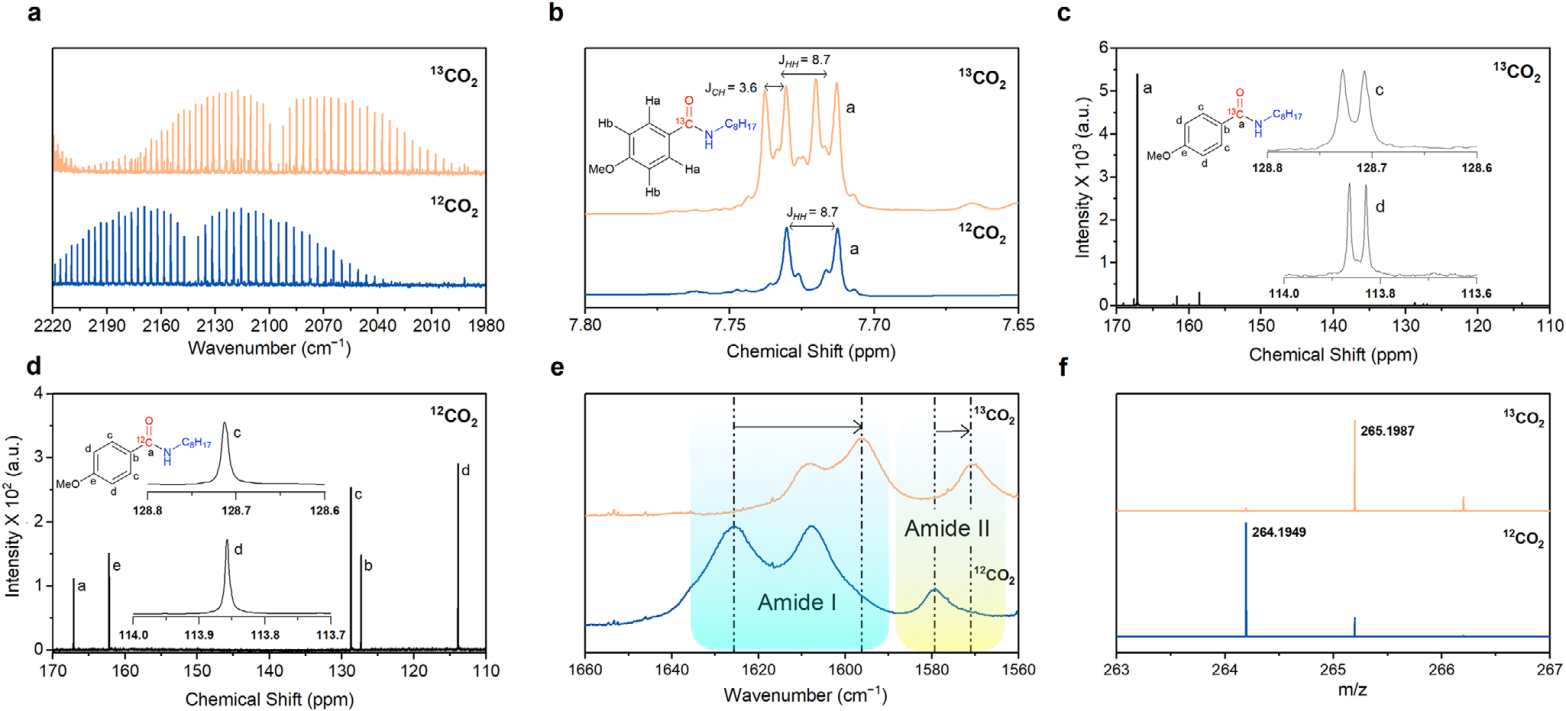
Isotopic labelling experiments using ^12^CO_2_ or ^13^CO_2_ and analysis of (a) reaction headspace by IR spectroscopy followed by (b‐f) corresponding benzamide product (**3**) characterization by (b) ^1^H (500 MHz) and (c, d) ^13^C (125 MHz) NMR spectroscopy, (e) IR spectroscopy and (f) LC‐MS. Reaction conditions: organic layer: iodoanisole (20 µmol, 1 equiv.), octylamine (100 µmol, 5 equiv.), Pd(OAc)_2_ (5 mol%), Xantphos (10 mol%), toluene (N_2_ sat., 2 mL); aqueous layer: CoCat1 (10 µM), RuPS (500 µM), NaHAsc (100 mM), NaHCO_3_ (100 mM) in H_2_O (CO_2_ sat., pH 6.8, 6 mL); 24 h illumination from blue LED (447 nm, 2.3 W), 25°C, 250 rpm orbital shaking.

When ^13^CO_2_ is used for the cascade reaction, the benzamide product contains a ^13^C carbonyl as a handle for characterization. This is clear from the ^1^H NMR spectrum, where the aromatic protons *ortho* to the amide are observed to be split into a doublet of doublets by the ^13^C (δ = 7.7 ppm, ^3^ *J_HH_* = 8.7 Hz, ^3^ *J_CH_* = 3.6 Hz), in contrast to the typical doublet observed when ^12^CO is inserted (δ = 7.7 ppm, ^3^ *J_HH_* = 8.7 Hz; Figures 2b and S5a). Quantitative ^13^C NMR spectroscopy also confirms the incorporation of ^13^C from ^13^CO_2_ through a carbonyl signal that is ∼100‐fold greater than when using ^12^CO_2_ due to the higher ^13^C abundance (δ = 167 ppm; Figure 2c vs 2d; carbonyl peak integrates to 100 carbons while *para* aromatic carbon integrates to 1 carbon when ^13^CO_2_ is used). The ^13^C carbonyl also splits the signals for the aromatic carbons *ipso*, *ortho* and *meta* into doublets (δ = 127.3 ppm, ^1^ *J_CC_* =  66.3 Hz; δ = 128.7 ppm, ^2^ *J_CC_* = 2.6 Hz; and δ = 113.8 ppm, ^3^ *J_CC_* = 4.4 Hz), in contrast to the singlets observed when ^12^CO_2_ is used in the reaction (compare insets to Figure 2c,d).

Furthermore, IR spectroscopy of the benzamide product shows the Amide I band, corresponding primarily to the vibration of the carbonyl [55, 56, 57], is distinctly shifted to lower wavenumber when ^13^CO_2_ is used (1625 to 1596 cm^−1^; Figure 2e, Figure S5b). This shift is attributed to the higher mass of the ^13^C affecting the C═O stretching frequency in the amide compared to ^12^C. The observed shift to lower wavenumber is 29 cm^−1^, which is reasonably concordant with the ∼36 cm^−1^ shift predicted for the mass change of the carbonyl unit using Hooke's Law for harmonic oscillators (which only accounts for two masses rather than the three present in this case) [58]. Lastly, mass spectrometry shows the additional mass of the ^13^C in the product when ^13^CO_2_ is used, with a peak at 265.1987 rather than 264.1949 *m/z* (calculated values are 265.1992 and 264.1958 *m/z*, respectively; Figure 2f). Thus, it is conclusive that the carbon from CO_2_ has been incorporated into the benzamide carbonyl, confirming that the biphasic reaction cascade described herein utilizes the carbon from CO_2_ in the synthesis of complex molecules.

### 2.5 Substrate Scope for CO_2_‐to‐Aminocarbonylation Cascade

To demonstrate the broad applicability of the CO_2_ to carbonylation cascade reaction, a range of coupling partners were screened to determine the functional group tolerance. This substrate scope included cyano, ester, ketone, phenyl, methoxy, nitro, and trifluoromethyl functionality on the aryl iodide together with functionalized benzylamine, naphthylmethylamine, and aniline (Figure 3). These reactions were run as the full photocatalytic reaction cascade starting with CO_2_ and using the standard conditions with RuPS/CoCat1 and 447 nm LED illumination.

**FIGURE 3:**
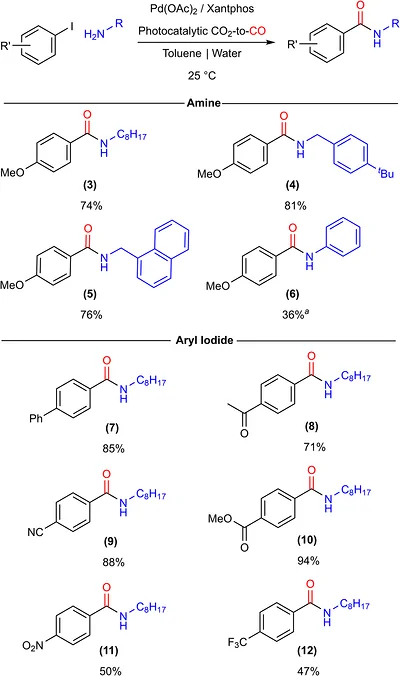
Substrate scope for the biphasic cascade using photocatalytic CO_2_ reduction to generate CO. Reaction conditions: organic layer: aryl iodide (20 µmol, 1 equiv.), amine (100 µmol, 5 equiv.), Pd(OAc)_2_ (5 mol%), Xantphos (10 mol%), in toluene (N_2_ sat., 2 mL); aqueous layer: CoCat1 (10 µM), RuPS (500 µM), NaHAsc (100 mM), NaHCO_3_ (100 mM) in H_2_O (CO_2_ sat., pH 6.8, 6 mL); 24 h illumination from blue LED (447 nm, 2.3 W), 25°C, 250 rpm orbital shaking. *^a^* 4 h illumination then 24 h at 80°C. All products were identified through ^1^H, ^13^C and 2D (HSQC, HMBC, COSY) NMR of the crude, followed by mass spectrometry, with values compared to the NMR spectra of isolated products for new compounds and comparison to literature values for known compounds. Quantification of cascade yields was by ^1^H NMR with an internal standard.

The variety of aryl iodide substrates was selected to show a range of functional groups with a variety of conjugative and inductively electron‐donating/withdrawing effects. *para*‐methoxy and phenyl groups enabled 74% and 85% yields of benzamide (**3**, **7**; Figure 3); while *para‐*aceto (**8**, 71%), *para‐*methylester (**10**, 94%) and *para*‐cyano (**9**, 88%) groups performed similarly or better. Conversely, nitro and trifluoromethyl groups resulted in yields of 50 and 47%, respectively (**11**, **12**; Figure 3). Varying the amine coupling with iodoanisole showed comparable yields of 74%–81% for benzylic or primary amines (Figure 3), with naphthylmethylamine (**5**, 76%) and *para‐^t^* butyl‐benzylamine (**4**, 81%) comparable to the standard octylamine substrate (**3**, 74%). Using a less nucleophilic amine, such as aniline, requires heating for the aminocarbonylation to proceed (**6**, 36% at 80°C).

Overall, the substrate scope trends indicate a wide range of carbonylative couplings are possible in photochemically initiated cascades with biphasic compartmentalization. Key constraints include that the reaction compartmentalization requires preferential solubility of the reagents in the organic layer. This results in a preference for lipophilic molecules, while increasing the nucleophilicity of the amine by employing secondary amines results in nucleophilic attack of CO_2_ and precipitation of unproductive carbamates; both of which are more restrictive on the scope of the amine. The examples shown focused on ambient temperature aminocarbonylation with aryl iodides, thus aryl bromides or chlorides were not examined.

### 2.6 Sonogashira and Suzuki Cascades

The biphasic cascade concept demonstrates a general reaction platform that compartmentalizes the light and dark reactions and enables different CO_2_ reducing photosystems to be used with various carbonylative organic reactions. Thereby, the concept provides flexibility in not only the substrate scope of a reaction, but also in driving different organic reactions. This breadth was examined by pairing an additional two carbonylative organic reactions, Sonogashira and Suzuki couplings, with both the RuPS/CoCat1 and CuPS/CoCat2 photosystems. The standard reaction conditions established above were used as the basis for each reaction, with some changes necessary to provide suitable conditions and reagents for each reaction in the organic layer, but no changes were made to reagents or concentrations in the aqueous layer. Thus, these reactions used an organic layer that contained aryl iodide (0.02 mmol, 1 equiv.), nucleophile (0.03 mmol, 1.5 equiv.), trioctylamine (0.06 mmol, 3 equiv.) as a base, Pd(OAc)_2_ (5 mol%) and ligand (10 mol%) in toluene (N_2_‐sat., 2 mL), which was layered onto water (CO_2_ sat., pH 6.8, 6 mL) containing catalyst (10 µM), photosensitizer (500 µM), NaHAsc (100 mM) and NaHCO_3_ (100 mM) under a CO_2_ atmosphere. The biphasic solutions were illuminated from below (447 nm for RuPS and 405 nm for CuPS, 2.3 W LED) with orbital convection (250 rpm) at 25°C for 4 h, after which they were heated to 80°C in a two‐stage sequence because the organic reactions require above ambient temperatures to proceed.

The first reaction examined in this reaction scope was the carbonylative Sonogashira alkyne coupling, which is a widely used process that couples aryl halides with CO and terminal alkynes to yield an alkynyl ketone using catalytic Pd with a Cu co‐catalyst [23, 24]. This reaction was demonstrated in the biphasic system by coupling 4‐iodoanisole (the aryl iodide) and 4‐ethynylanisole (the nucleophile) with the reaction catalyzed by Pd(OAc)_2_ (5 mol%) and Xantphos (10 mol%), as well as CuI (20 mol%) as the standard co‐catalyst for Sonogashira coupling (Figure 4b). Trioctylamine was also included as a water insoluble base (0.06 mmol, 3 equiv.), because, unlike the amine in the aminocarbonylation, the nucleophile is not a suitable base. The organic reaction components were dissolved in toluene and added onto the optimized aqueous layer under CO_2_ atmosphere. Illumination (4 h) and then heating the reaction (72 h, 80°C) resulted in a yield of 40% dianisyl‐propynone **13** when using the RuPS/CoCat1 photosystem, and a 68% yield of **13** when using the CuPS/CoCat2 photosystem (Figure 4b). This demonstrates that the approach is compatible with carbonylative Sonogashira couplings.

**FIGURE 4:**
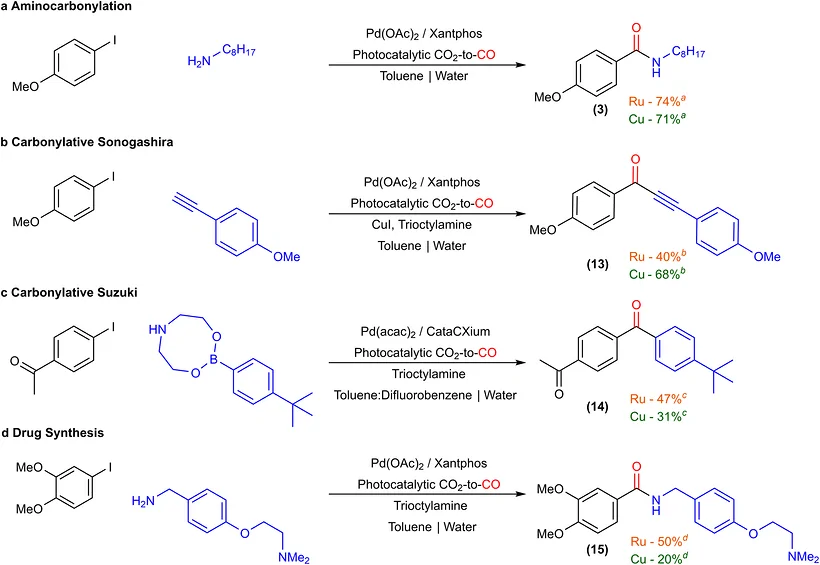
Comparison of yields from biphasic reactions using ruthenium or copper photosystems to convert CO_2_ to CO for carbonylation reactions. Photosystems: [Ru] = RuPS/CoCat1 or [Cu] = CuPS/CoCat2 with 2.3 W LED illumination at 447 nm or 405 nm, respectively. Reaction conditions: organic layer: aryl iodide (20 µmol, 1 equiv.), Nucleophile (Nu), Pd (5 mol%), Xantphos (10 mol%), in toluene (N_2_ sat., 2 mL); aqueous layer: CO_2_‐to‐CO photosystem, NaHAsc (100 mM), NaHCO_3_ (100 mM) in H_2_O (CO_2_ sat., pH 6.8, 6 mL); 25°C, 250 rpm orbital shaking. All products were identified through ^1^H, ^13^C and 2D (HSQC, HMBC, COSY) NMR of the crude, followed by mass spectrometry; yield quantification by ^1^H NMR with internal standard. *^a^* Nu (100 µmol, 5 equiv.), 24 h. *^b^* Nu (30 µmol, 1.5 equiv.) with trioctylamine (60 µmol, 3 equiv.) and CuI (20 mol%) also added to toluene layer; reaction heated to 80°C for 72 h after 4 h illumination. *^c^* CataCXium (10 mol%) in place of Xantphos, Nu (30 µmol, 1.5 equiv.) and trioctylamine (60 µmol, 3 equiv.) in toluene:1,2‐difluorobenzene (4:1) solvent; reaction heated to 80°C for 24 h after 4 h illumination. *^d^* Nu (30 µmol, 1.5 equiv.) with trioctylamine (60 µmol, 3 equiv.) and reaction heated to 80°C for 24 h after 4 h illumination.

The second reaction examined was the carbonylative Suzuki coupling, which is another widely used reaction and uses catalytic Pd to couple an aryl halide with CO and a boronic acid to form a ketone. Incorporation into the bilayer is convenient for Suzuki reactions, as they are known to be water‐tolerant and sometimes even accelerated by it [59, 60]. A Suzuki reaction was demonstrated in the bilayer using 4‐iodoacetophenone (the aryl iodide) with 4‐*^t^* butyl‐phenylboronic acid (the nucleophile), masked as a diethanolamine complexed heterocyclic (DABO) boronate (Figure 4c). The cataCXium ligand was used with Pd(acac)_2_ in place of Xantphos following literature precedent [61], while the boronic acid was masked as a boronate to avoid use of an external base and to reduce its water solubility. The high polarity of the boronate still limited its solubility in toluene, but the addition of 1,2‐difluorobenzene in a 1:4 ratio with toluene enabled dissolution while also maintaining the biphasic separation. Further increases of the 1,2‐difluorobenzene ratio resulted in solvent mixtures denser than water, which inverted the biphase. These modifications demonstrate both limitations of the system, in terms of solubility requirements as discussed in the substrate scope, and advantages, in terms of flexibility to change and mix solvents. After layering the organic solution onto the aqueous layer, illumination (4 h) followed by heating (24 h, 80°C) when using the RuPS/CoCat1 photosystem resulted in 47% yield of the aceto and tert‐butyl substituted benzophenone **14**, while use of the CuPS/CoCat2 photosystem resulted in 31% yield of **14** (Figure 4c). Thus demonstrating that the biphasic concept is compatible with aminocarbonylation and the Sonogashira and Suzuki carbonylative couplings.

### 2.7 Using CO_2_ as a Carbon Source for Pharmaceutical Synthesis

The upcycling of CO_2_ is important to make carbon management economically viable, and the use of CO_2_ in pharmaceutical production represents perhaps the largest possible waste‐to‐value conversion, that is, converting a pollutant into high‐value fine chemicals. Consequently, a reaction cascade was assembled to utilize the carbon from CO_2_ in pharmaceutical synthesis within one‐pot. The target drug was itopride **15**, which is used to treat gastrointestinal disease by inhibiting dopamine D2 receptors and acetylcholinesterase [62], and has a market size expected to grow from $150 M to $250 M USD by 2032 [63]. This synthesis requires carbonylative coupling of 2‐methoxy‐4‐iodoanisole (the aryl iodide) and 4‐(2‐dimethylaminoethoxy)benzylamine (the nucleophile) to form a new amide functional group.

To establish a benchmark prior to assembling a cascade reaction, itopride was first synthesized in typical fashion from the starting materials using exogenous CO in excess [64]. The conditions were therefore 2‐methoxy‐4‐iodoanisole (0.02 mmol, 1 equiv.), 4‐(2‐dimethylaminoethoxy)benzylamine (1.5 equiv.), trioctylamine (3 equiv.), Pd(OAc)_2_ (5 mol%) and Xantphos (10 mol%) in toluene (N_2_‐sat., 2 mL). Trioctylamine was included as a water insoluble base (3 equiv.) to prevent use of the amine substrate as a base. Placing the reaction under a CO atmosphere (excess CO) and heating at 80°C for 24 h yielded itopride with 60% yield.

To enable itopride synthesis where CO_2_ is added rather than CO, the organic reagents were dissolved in toluene as above, and added onto water (CO_2_ sat., pH 6.8, 6 mL) under a CO_2_ atmosphere containing a CO_2_ reduction photosystem using the optimized conditions, that is, catalyst (10 µM), photosensitizer (500 µM), NaHAsc (100 mM), and NaHCO_3_ (100 mM). The solution was then illuminated for 4 h before being placed at 80°C for 24 h with use of the RuPS/CoCat1 and CuPS/CoCat2 photosystems resulting in 50% and 20% respective yields of itopride **15** (Figure 4d). The RuPS/CoCat1 system thus enables the use of CO_2_ in a biphasic system with yields within error (± 10%) of those from the addition of excess CO in water‐free conditions, which supports the plausibility of replacing fossil‐derived CO with CO_2_ for fine chemical synthesis.

## 3 Conclusion

Inspired by the separation of the light and dark reactions of photosynthesis in the chloroplast, this work demonstrates compartmentalized reaction cascades that enable CO_2_ to be used in the synthesis of organic molecules in one‐pot under visible illumination, including solar light. Photocatalytic CO_2_ reduction to CO in aqueous media enables effective delivery of CO into an immiscible organic solvent in a bilayer configuration, wherein Pd‐catalyzed carbonylation reactions can proceed with high yield. The applicability of compartmentalization is shown by employing different CO_2_ reduction photosystems and their pairing with a range of carbonylative couplings to synthesize a range of organic products. This compartmentalized reaction cascade design can thus be considered as a general carbonylative Pd cross‐coupling platform that enables CO_2_ to be used as a bioinspired carbonyl source in aminocarbonylation as well as the carbonylative Sonogashira and Suzuki reactions, without handling toxic CO.

The reaction compartmentalization significantly increases the biomimicry of CO_2_ utilization for organic synthesis by enabling the light‐driven generation and utilization of CO from CO_2_ for chemical synthesis in the same pot. These findings represent a step toward utilizing the simplest building blocks, in the form of CO_2_, to assemble complex molecules in a similar way as biology has achieved through evolution. Future work may couple the reaction with water oxidation to better mimic a chloroplast by using nature's electron source. Thereby, this reaction platform offers a pathway to further transformations utilizing CO_2_ and moves in vitro chemistry nearer to the elegant molecule‐building machinery of nature.

## Author Contributions

**Sampurna Mitra**: data curation, writing – original draft, conceptualization, investigation, writing – review and editing, visualization, methodology, project administration, formal analysis, software. **David M. Vahey**: investigation, methodology, writing – review and editing, formal analysis. **Shannon A. Bonke**: supervision, methodology, conceptualization, investigation, writing – original draft, writing – review and editing, project administration. **Erwin Reisner**: supervision, resources, funding acquisition, investigation, conceptualization, writing – review and editing.

## Conflicts of Interest

The authors declare no conflicts of interest.

## Supporting information



Additional supporting information can be found online including supplementary notes, experimental details [65, 66], synthesis procedures for Na_4_[Co *meso*‐tetra(sulfonatophenyl)porphyrin] (CoCat1) [8], [Co *meso*‐tetra(N‐methyl)pyridyl porphyrin]Cl_4_ (CoCat2) [48], and Na_4_[Cu(5,6‐disulfonato‐bathocuproine)(2,7‐bissulfonato‐‌xantphos)](BF_4_) (CuPS) [9, 51, 52] and characterization of all synthesized molecules [67, 68, 69, 70, 71, 72]. **Supporting File**: anie74748‐sup‐0001‐SuppMat.pdf.

## Data Availability

Primary data supporting the findings of this study are available from the University of Cambridge open‐access data repository: https://doi.org/10.17863/CAM.133634.
