# Supplementary material for: One‐Pot Biphasic Photocatalytic CO2‐to‐Carbonylation Cascades
Source: Angew Chem Int Ed Engl. 2026 Sep 25;65(46):e5220236. doi: 10.1002/anie.5220236 (PMC13749338; doi:10.1002/anie.5220236)
Supplement: Supplementary file 1 [file ANIE-65-e5220236-s001.pdf]

Supplementary Information for

One-Pot Biphasic Photocatalytic CO_2-to-Carbonylation Cascades

Sampurna Mitra, David M. Vahey, Shannon A. Bonke,* Erwin Reisner*

Yusuf Hamied Department of Chemistry, University of Cambridge, Lensfield Rd, Cambridge CB2 1EW, UK.

* Correspondence to: Dr S. A. Bonke (sabonke@ch.cam.ac.uk) and Prof E. Reisner (reisner@ch.cam.ac.uk)

Contents

Supplementary Notes	2-3
Experimental and Characterisation	4-17
Supplementary Tables	18-22
Supplementary Figures	23-25
^{1}H and ^{13}C NMR Spectra	26-34
Mass Spectra	35-37
Supplementary References	38

Supplementary Note 1: Optimisation of the Photocatalytic System

Optimisation of the photocatalytic system started with a solution of CoCat1 (10 µM), RuPS (500 µM) and NaHAsc (100 mM) in a CO_2-saturated bicarbonate solution (100 mM, pH 6.8, 2 mL).[1] This system produced 23±1 µmol of CO ($Yield_{CO}$) in 24 h with 84±3% selectivity towards CO over H_2 and a catalyst turnover number and frequency for CO of 1129±11 and 47±<1, respectively (Fig S1a and Table S1).

Variation of the catalyst concentration showed a linear increase of $Yield_{CO}$ with catalyst concentration from 0.1 to 10 µM (1.4±0.1 to 23±1 µmol; r^2 = 0.99). Further increasing the catalyst concentration to 30 µM nearly doubles the $Yield_{CO}$ (38±2 µmol), but increasing to 50 µM does not yield further improvement. The lack of improvement is attributed to overlapping absorption bands from the catalyst and photosensitiser (425 and 456 nm, respectively; Fig S2), which results in the catalyst significantly shading the photosensitiser beyond 30 µM. The CO selectivity is 87±2% in the 10 to 50 µM catalyst range. The 13±2% H_2 formation is primarily attributed to photodegradation of the Rubpy photosensitiser, which is known to generate $[Ru(bpy)_2(CO)H]^+$ and $[Ru(bpy)_2(CO)_2]^{2+}$ that catalyse hydrogen evolution and lower the observed selectivity.[2]

Varying the photosensitiser concentration whilst maintaining the catalyst at 10 µM reveals that $Yield_{CO}$ increases in the range of 10 to 250 µM (0.9±0.1 to 24±1 µmol CO), consistent with increased photon utilisation, but there is no improvement with further increases (Fig. S1b). To further increase the $Yield_{CO}$, the reaction volume was increased, which increased the $Yield_{CO}$ linearly from 23±1 to 35±8, 74±3 and 83±1 µmol for 2, 4, 6, and 8 mL, respectively (r^2 = 0.99; Fig S1c). This corresponds to 11±2 $µmol_{CO}$ mL^{-1} with 83±1% CO selectivity across all volumes tested, which indicated that degradation of the photosensitiser during the experiment (24 h) leads to incomplete use of photons over time. The loading of NaHAsc ensures the electron donor is always in 10-fold excess to the photosensitiser.

Supplementary Note 2: CO Dissolution in Organic Layer

The fraction of CO dissolved in the toluene layer was quantified as ~16% (8 of 50 μmol; Table S2), which corresponds to 12 of 73 μmol detected in the headspace without the organic layer, whereas the CO in the headspace has decreased ~45% with the addition of the toluene layer (40 vs 73 μmol). The residual difference (21 μmol) is attributed to CO_2 dissolution/extraction in the toluene lowering the aqueous CO_2 concentration available to the catalyst and mixing through orbital convection that results in temporary emulsion formation that scatters light and shades the photosensitiser; both lead to decreased CO production. In the full cascade system, there is an additional contribution from the amine capturing CO_2 as carbamate and thereby lowering the aqueous CO_2 concentration available to the catalyst.

Supplementary Note 3: Post Reaction UV-Visible Spectrophotometry

Comparing UV-Vis spectra of the aqueous layer before and after the 24 h full cascade reaction shows that bleaching of the photosensitiser reduces its absorption at 455 nm by 40% (A = 0.77 to 0.46; same dilution factor used; Fig. S4c). Post-reaction, the non-photosensitiser component below 500 nm can be fit with oxidised ascorbate, but a residual catalyst contribution cannot be fully excluded (the ascorbate gives a better fit to low-wavelength shoulder). A new absorption band is also observed at ~525 nm after 24 h and attributed to a degradation product of the photosensitiser, as the visible absorption is concordant with literature assignment from tests excluding catalyst.[1]

Experimental

Chemicals and Treatment of Data. All commercially available chemicals were bought from either Sigma Aldrich, Alfa Aesar, Thermo Scientific or Porphychem and used as supplied. Deionised water was used throughout (18 MΩ cm). Unless otherwise stated, each set of experiments was performed in duplicate and errors are given as the unweighted mean ± one standard deviation; all values stated in the text or plotted have a minimum error value of 5%. Synthesis of Na_4[Co *meso*-tetra(sulfonatophenyl)porphyrin] (CoCat1),[3] [Co *meso*-tetra(N-methyl)pyridyl porphyrin]Cl_4 (CoCat2),[3] and Na_4[Cu(5,6-disulfonato-bathocuproine)(2,7-bissulfonato-xantphos)](BF_4) (CuPS)[4–6] followed or adapted literature procedures (details and characterisation below).

Photocatalytic Reaction Cascade (Method A). The aqueous layer (6 mL) was prepared from stock solutions of each reagent, delivered into crimp-cap vials sealed with septa (PTFE coated for T>50 °C) and purged through inlet and outlet needles for 10 min with CO_2, including 2 vol.% CH_4 as an internal standard. The organic layer was prepared by dissolving Ar-I (0.02 mmol, 1 equiv.), $Pd(OAc)_2$ (5 mol%) and Xantphos (10 mol%) in dry toluene (2 mL) under N_2, prior to the addition of Nu (0.1 mmol, 5 equiv.). The toluene solution was then layered on top of the sealed aqueous layer by syringe, before the vials were placed in an LED photoreactor (Treellum Technologies) with a thermostatically controlled heating block at 25 °C with 250 rpm orbital convection; wherein the vials were illuminated from the bottom with a 405 (CuPS) or 447 (RuPS) nm 2.3 W LED with 20 nm spectral width.[7,8]

The reaction headspace was sampled via gastight syringe (Hamilton) and analysed by gas chromatography (Shimadzu Tracera GC-2010 Plus with a discharge ionisation detector) using a molecular sieve column (Restek 5Å PLOT, 0.53 mm ID, 30 m) at 60 °C. H_2 and CO peak areas were converted to moles using calibrated response factors and the 2 vol.% CH_4 internal standard. The organics were then extracted from the solution with EtOAc, and the solvent removed *in vacuo* prior to NMR and MS. Product identification was via ^{1}H and ^{13}C NMR incl. HSQC, HMBC and COSY followed by ESI-MS. 1,3,5-trimethoxybenzene (0.02 mmol) was used as an internal standard for NMR quantification using the aromatic protons of the product. NMR signals were compared to literature values for known compounds,[9–

[14] while new compounds were synthesised and purified in larger batches via Method B for subsequent comparison, with full characterisation thereof provided below.

For kinetic studies, 50 µL aliquots of toluene were removed and diluted into 450 µL EtOAc containing acetic acid to quench the reaction. The accumulated time points were then analysed by a GC-MS equipped with an FID detector and peak areas were compared to a calibration curve (Fig S6).

For isotopic labelling, the aqueous solution was degassed and placed under a $^{13}CO_2$ atmosphere prior to reaction, and $NaH^{13}CO_3$ was used. Evolved gas was analysed by transferring the vial headspace into an evacuated gas infrared cell (SpecAc, 10 cm path length, KBr windows) and collecting a FT-IR transmission spectrum. Organics were then extracted from the solution for further analysis as above.

General Synthesis of Carbonylative Amination Compounds (Method B). Ar-I (0.2 mmol, 1 equiv.), $Pd(OAc)_2$ (5 mol%), and Xantphos (10 mol%) were dissolved in dry toluene (2 mL) prior to the addition of Nu (1.0 mmol, 5 equiv.). The reaction was stirred at 25 °C for 24 h under a CO atmosphere (balloon). Solvent was then removed *in vacuo* and the product was purified by column chromatography (SiO_2 | Hexane:Et_2O) to yield the carbonylated product. Characterisation of each compound and any deviations in method are provided below.

Physical Characterisation. Infrared spectroscopy was carried out on a Thermo Scientific Nicolet iS50 IR spectrometer. UV–vis spectroscopy was conducted on an Agilent Cary 60 UV–vis spectrophotometer using quartz cuvettes with 10 mm path length. ^{1}H and ^{13}C nuclear magnetic resonance spectroscopy (NMR) was recorded on Bruker 400, 500 and/or 700 MHz Spectrometers under standard conditions (298 K) unless otherwise indicated. All ^{1}H chemical shifts were reported in ppm and have been internally calibrated to the residual protons of the deuterated solvent or the internal standard present. ^{19}F (decoupled) nuclear magnetic resonance spectroscopy (NMR) was recorded on a Bruker 400 Hz Spectrometer under standard conditions (298 K). Elemental analyses (CHN analysis using a Perkin-Elmer 240 Elemental Analyzer) and Mass Spectrometric analyses (using Waters Xevo G2-S, Waters Synapt G2-Si, ThermoFinnigan Orbitrap Classic, Waters LCT Premier, Micromass Quattro LC, Perkin Elmer Turbomass GCMS, Waters SQD2 with Waters H-Class UPLC, Waters Vion IMS Qtof) were respectively conducted by the Microanalysis and Mass Spectrometry Services of the Yusuf Hamied Department of Chemistry, University of Cambridge.

GC-MS Calibration. A series of calibration solutions containing both 4-methoxy-N-octylbenzamide **(3)** and 4-Iodoanisole **(1)** at 0.01, 0.02, 0.05, 0.1, 0.5, 0.8, and 1 mM in EtOAc were analysed on an Agilent 5977C GC/MSD with a fused silica GC column (30 m, Dia. 0.250 mm, film 0.25 μm; Agilent HP-5MS 19091S-433UI). Oven programme: 80 °C (4 min), ramp to 200 °C (20 °C/min), hold (11 min), ramp to 260 °C (20 °C/min) and hold (11 min). This yielded a linear response of the peak area vs. concentration (r^2 = 0.99; Fig. S6), using which the concentration of iodoanisole and benzamide was interpolated.

Measuring CO solubility in the toluene layer. DI water and 100 mM bicarbonate solution in water (6 mL), with and without toluene (2 mL), were purged for 10 mins with N_2 or CO_2, including 2 vol.% CH_4 as an internal standard, and 50 μmol of CO was externally injected into the solutions. The solutions were shaken at 25 °C for 1 h, and the headspace gas was analysed by GC. The results reveal that negligible gas is taken up by the water or bicarbonate solutions; however, there is a decrease of 8.0 ± 0.5 μmol of CO in the headspace with the presence of toluene (Table S2); which is broadly consistent with literature values.[15]

Synthesis of CoCat1 (Na_4[Co tetra(sulfonatophenyl)porphyrin]). The method was adapted from literature procedures.[3] $CoCl_2 \cdot 6H_2O$ (1.4 g, 6 mmol) and *meso*-tetra(sulfonatophenyl)porphyrin (0.9 g, 1 mmol) were dissolved in DI water (25 mL), the pH was adjusted to 8 with 1 M NaOH and the solution was stirred for 24 h at refluxing conditions. The solution was filtered to remove insoluble materials before loading onto a cation exchange column (Amberlyst 15 resin) for the removal of residual Co cations. The solvent was then removed under vacuum to obtain a purple solid as the final product. Yield = 648 mg (0.5 mmol, 50%).

^{1}H NMR (400 MHz, D_2O) δ 9.29 (s, 8H), 8.43 (d, J = 7.5 Hz, 8H), 8.26 (d, J = 8.5 Hz, 8H). **^{13}C NMR** (100 MHz, D_2O) δ 144.04, 143.76, 142.19, 134.76, 134.49, 124.08, 118.78. **Elemental analysis** calculated for $C_{44}H_{24}Co^{III}N_4Na_4ClO_{12}S_4 \cdot 10H_2O$ (1115.3): C 40.80, H 3.42, N 4.32%. Found: C 40.29, H 3.20, N 4.96%. **ESI-MS** (−, MeOH): m/z calculated for $[C_{44}H_{24}N_4S_4O_{12}Co]^{3-}$: 328.9874. Found: 328.9881. **UV-Vis** (H_2O): λ_{max} (nm) (ε, M^{-1} cm^{-1}) 425 (21.5×10^4), 538 (1.13×10^4), 573(sh).

Synthesis of CoCat2 ([Co tetra(N-methyl)pyridino porphyrin]Cl_4). The method was adapted from literature procedures.[3] [*meso*-tetra(4-N-methylpyridyl)porphyrin]I_4 (500 mg, 0.3 mmol) and

$Co(OAc)_2 \cdot 4H_2O$ (814 mg, 3.3 mmol) were refluxed for 16 h in H_2O (40 mL). EDTA (1.49 g, 4 mmol) was added to the cooled solution to coordinate residual Co^{2+} as an anionic complex, which was separated from the product on an anion exchange column (Amberlite IRA-410, Cl-form) after filtration. The solvent was removed in vacuo, the product was dissolved in warm MeOH and precipitated with Et_2O before it was collected by Büchner filtration. The product was then dissolved in warm H_2O and precipitated with acetone twice to remove residual salt before final collection by Büchner filtration and washing with acetone and Et_2O. Yield: 86 mg (0.09 mmol, 27%).

Elemental analysis: Calculated for $C_{44}H_{36}N_8CoCl_4 \cdot 5H_2O$ (967.57 g mol^{-1}): C 54.62, H 4.79, N 11.58%. Found: C 54.96, H 4.71, N 11.53%. **ESI-MS** (+, MeOH): m/z calculated for $C_{44}H_{36}N_8Co^{4+}$: 183.8099. Found: 183.8093. **UV-Vis** (H_2O): λ_{max} (nm) (ε, M^{-1} cm^{-1}) 432 (12×10^4).

Synthesis of the tetra(N-methyl)pyridino porphyrin]. The method was adapted from literature procedures.[3] *meso*-tetra(4-pyridyl)porphyrin (615 mg, 1 mmol) and MeI (2.5 mL, 40 mmol) in DMF (60 mL, anhydrous) were heated at 145 °C for 16 h. The product was precipitated with Et_2O (~400 mL), collected by Büchner filtration and washed with acetone and Et_2O. The product was redissolved in H_2O and impurities extracted with EtOAc prior to precipitation with acetone and washing with acetone and Et_2O. Yield: 1.255 g (0.82 mmol, 82 %).

Elemental analysis: Calculated for $C_{44}H_{38}N_8I_4 \cdot 7H_2O \cdot C_3H_7NO \cdot NaI$ (1535.55 g mol^{-1}): C 37.76, H 3.87, N 8.21%. Found: C 36.79, H 4.13, N 8.20%. **ESI-MS** (+, MeOH): m/z calculated for $C_{44}H_{38}N_8^{4+}$: 169.5805. Found: 169.5862. **^{1}H NMR** (DMSO-d^6, 400 MHz): δ (ppm): 9.49 (d, 8H, 9.5 Hz), 9.21 (br, s, 8H), 9.00 (d, 8H, 9.0 Hz), 4.73 (s, 12H), −3.10 (s, 2H). **^{13}C NMR** (DMSO-d6, 100 MHz): δ (ppm): 156.27, 144.24, 132.07, 118.07, 115.83, 47.94. **UV-Vis** (H_2O): λ_{max} (nm; ε, M^{-1}cm^{-1}) 422 (1×10^4).

Synthesis of CuPS (Na_4[Cu(5,6-disulfonato-bathocuproine)(2,7-bissulfonato-xantphos)](BF_4)). The method was adapted from literature procedures.[4] Cu_2O (1.000 g) was refluxed at 55 °C in dry MeCN (30 mL) with HBF_4 (5 mL, 47% solution) under N_2 atmosphere until the solids dissolved. The hot solution was filtered and cooled to rt under inert conditions. White colourless crystals were formed which were collected through filtration, washed with dry ether and dried under vacuum. The final product was stored under nitrogen at low temperatures to avoid oxidative decomposition by air. $[Cu(MeCN)_4]BF_4$ (11 mg,

0.03 mmol) and L2 (25 mg, 0.03 mmol) were stirred in anhydrous MeOH (10 mL) at rt for 1 h under N_2 atmosphere. L1 (18 mg, 0.03 mmol) dissolved in anhydrous MeOH (5 mL) kept under N_2 was added dropwise to the above solution and further stirred for 2 h. The solution was filtered using a PTFE filter, adding it dropwise to a large excess of ether. The yellow precipitate was collected through filtration, dried in vacuo. Yield: 31.2 mg (0.02 mmol, 67 %).

^{1}H NMR (500 MHz, Methanol-d^4) δ 8.26 (d, J = 1.9 Hz, 2H), 8.22-7.59 (m, 14H), 7.48-7.06 (m, 20H), 2.60 – 2.32 (m, 6H), 1.81 (t, J = 16.5 Hz, 6H). **Elemental analysis**: Calculated for $C_{65}H_{48}CuN_2Na_3O_{13}P_2S_4 \cdot 4H_2O \cdot 1NaBF_4$ (1569.59): C 49.74, H 3.6, N 1.78%. Found: C 49.58, H 3.44, N 1.58%. **ESI-MS** (+, MeOH): m/z calculated for $[C_{65}H_{48}CuN_2Na_4O_{13}P_2S_4]^+$: 1409.0396. Found: 1409.0355. **UV-Vis** (MeOH): λ_{max} (nm) (ε M^{-1} cm^{-1}) 390 (3.7×10^3). Spectroscopic data are in agreement with those previously reported.[4]

Synthesis of L1 for CuPS.

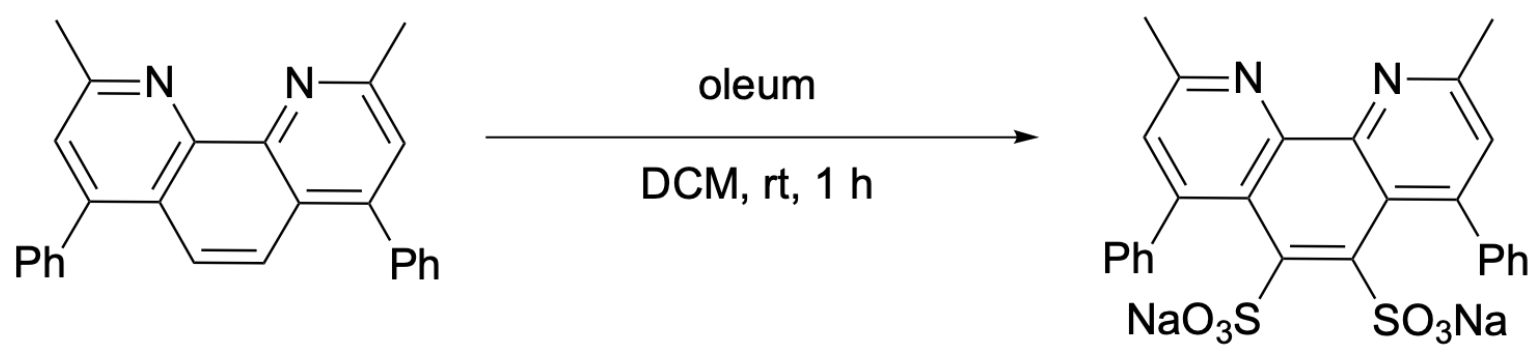

The ligand L1 (5,6-disulfonato-bathocuproine) was prepared according to the previous literature with minor modifications.[4] Oleum (fuming 30%, 4 mL) was added dropwise to a bathocuproine (714 mg, 1.98 mmol) solution in dry DCM (30 mL) at 0 °C and stirred for 1 h. The reaction was warmed to room temperature following the addition of dry diethyl ether (40 mL) to obtain a yellow precipitate, which was collected by filtration. The solid was dissolved in H_2O and passed through a cation exchange column to maximise the yield of the sodium form of the product. The solvent was removed, and the solid was redissolved in MeOH and filtered using a 0.22 µm Millex-GP syringe filter to completely remove undissolved inorganic salts. Finally, the solvent was removed to dryness, and a yellow product was obtained. Yield: 429 mg (0.76 mmol, 38 %).

^{1}H NMR (500 MHz, Methanol-d^4) δ 8.10 – 8.00 (m, 6H), 7.99-7.93 (m, 2H), 7.75-7.67 (m, 4H), 3.10 (s, 6H). Spectroscopic data are in agreement with those previously reported.[4]

Synthesis of L2 for CuPS.

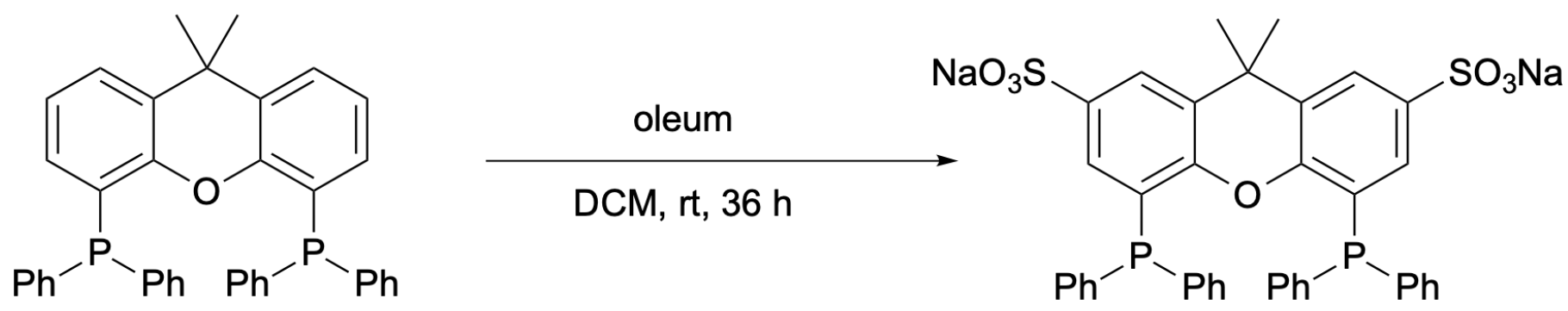

The ligand L2 (2,7-bissulfonato-xantphos) was prepared according to the previous literature with minor modifications.[4] Xantphos (500 mg, 0.86 mmol) was dissolved in dry DCM, and the mixture was cooled to 0 °C before the dropwise addition of concentrated sulphuric acid (98%, 1.2 mL). DCM was removed under vacuum, and oleum (fuming 30%, 2 mL) was added in a dropwise fashion, keeping the reaction at 0 °C and then stirred at rt for 36 h. The reaction was again cooled to 0 °C, followed by the addition of N_2-purged ice-cold water (20 mL). The resulting white precipitate was collected through filtration and redissolved in a minimum amount of H_2O. The solution was neutralised using a saturated NaOH solution. The solution was freeze-dried to obtain a fine white solid product, which was dissolved in MeOH/EtOH (1:2) and filtered using a 0.22 µm Millex-GP syringe filter to remove insoluble materials like oxidised phosphine monoxides and inorganic salts. MeOH/EtOH was evaporated and the solid was passed through a cation-exchange column to maximise the sodium form of the product. The aqueous solution was freeze-dried to yield the final white powder. Yield: 400 mg (0.5 mmol, 60%)

^{1}H NMR (500 MHz, Methanol-d^4) δ 8.00 (d, J = 2.1 Hz, 2H), 7.30 – 7.16 (m, 22H), 1.68 (s, 6H). Spectroscopic data are in agreement with those previously reported.[4]

Characterisation of Amides and Ketones.

4-methoxy-N-octylbenzamide (**3**)

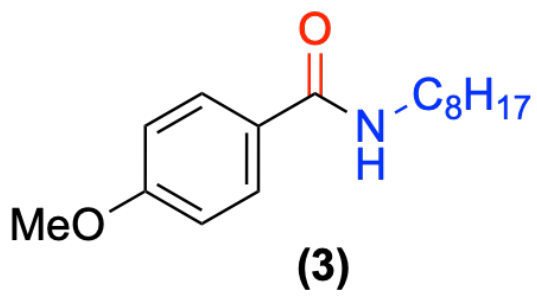

(3)

Repeating the reaction with standard conditions from the literature,[16] Ar-I (0.1 mmol, 1 equiv.), $Pd(OAc)_2$ (2 mol%), and Xantphos (2 mol%) and DABCO (0.30 mmol, 3 equiv.) were dissolved in dry toluene (2 mL) prior to the addition of Nu (0.15 mmol, 1.5 equiv.). The reaction was stirred at 25 °C for 24 h under a CO atmosphere (balloon). Solvent was then removed *in vacuo,* and the product was analysed via ^{1}H NMR, resulting in a 91 % crude NMR yield.

Reaction by Biphasic Cascade (Method A, see Experimental Methods) gave a crude yield of 74% by ^{1}H NMR. Crude ^{1}H NMR was matched with values from the isolated compound in the literature and with the isolated compound mentioned below.[9] ESI-MS (+, MeOH): m/z calculated for $C_{16}H_{25}O_2N + H^+$: 264.1964. Found: 264.1950.

Reaction by General Batch Synthesis (Method B, see Experimental Methods) yielded the isolated product after column chromatography (SiO_2 | Hexane:Et_2O, 7:3; Rf = 0.4) as *a crystalline pale pink solid*.[9] Yield: 52 % (27.5 mg, 0.10 mmol).

^{1}H NMR (700 MHz, $CDCl_3$) δ (ppm): 7.72 (d, *J* = 8.5 Hz, 2H), 6.89 (d, *J* = 8.4 Hz, 2H), 6.22 (s, 1H), 3.82 (s, 3H), 3.40 (q, *J* = 6.7 Hz, 2H), 1.58 (m, *J* = 7.4 Hz, 2H), 1.35 (p, *J* = 6.9 Hz, 2H), 1.28 (m, *J* = 10.7, 5.9 Hz, 8H), 0.87 (t, *J* = 6.9 Hz, 3H). **^{13}C NMR** (176 MHz, $CDCl_3$) δ (ppm): 167.17, 162.12, 128.74, 127.22, 113.78, 55.48, 40.20, 31.91, 29.85, 29.42, 29.33, 27.15, 22.75, 14.20. **ESI-MS** (+, MeOH): m/z calculated for $C_{16}H_{25}O_2N + H^+$: 264.1964. Found: 264.1949. **IR** (ATR): 3333, 2962, 2917, 2848, 1625, 1606, 1532, 1493, 1463, 1240, 1180 cm^{-1}. **UV-Vis** (EtOAc): λ_{max} 252, 272, 282 nm. **m.p.** 63-65 °C.

N-(4-(tert-butyl)benzyl)-4-methoxybenzamide (**4**)

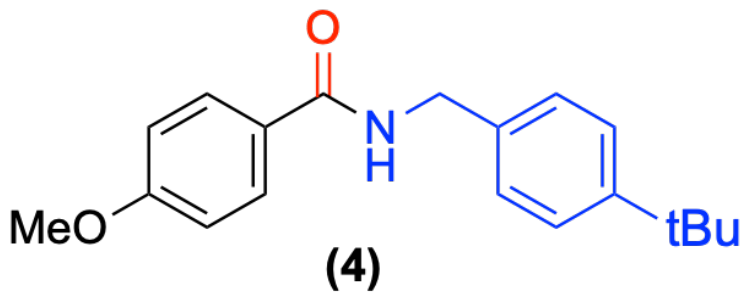

(4)

Reaction by Biphasic Cascade (Method A) gave a crude yield of 81% by ^{1}H NMR. Crude ^{1}H NMR was matched with values from the isolated compound synthesised below. ESI-MS (+, MeOH): m/z calculated for $C_{19}H_{23}O_2N + H^+$: 298.1807. Found: 298.1804.

Reaction by General Batch Synthesis (Method B) yielded the isolated product after column chromatography (SiO_2 | Hexane:Et_2O, 1:1; R_f = 0.2) as a *crystalline pale pink solid*. Yield: 59% (35.1 mg, 0.12 mmol)

^{1}H NMR (700 MHz, $CDCl_3$) δ (ppm): 7.76 (d, *J* = 8.9 Hz, 2H), 7.37 (d, *J* = 8.4 Hz, 2H), 7.29 (d, *J* = 8.5 Hz, 2H), 6.90 (d, *J* = 8.9 Hz, 2H), 6.41 (s, 1H), 4.59 (d, *J* = 5.6 Hz, 2H), 3.83 (s, 3H), 1.32 (s, 9H). **^{13}C NMR** (176 MHz, $CDCl_3$) δ (ppm):166.93, 162.27, 150.67, 135.47, 128.88,127.86, 126.84, 125.77, 113.83, 55.50, 43.89, 34.64, 31.46. **ESI-MS** (+, MeOH): m/z calculated for $C_{19}H_{23}NO_2 + H^+$: 298.1807. Found: 298.1804. **IR** (ATR): 3307, 2950, 2915, 1623, 1604, 1546, 1504, 1463, 1434, 1322, 1310, 1255, 1233, 1175, 1029 cm^{-1}. **UV-Vis** (EtOAc): λ_{max} 253 nm. **m.p.** 146-148 °C.

4-methoxy-N-(naphthalen-1-ylmethyl)benzamide (**5**)

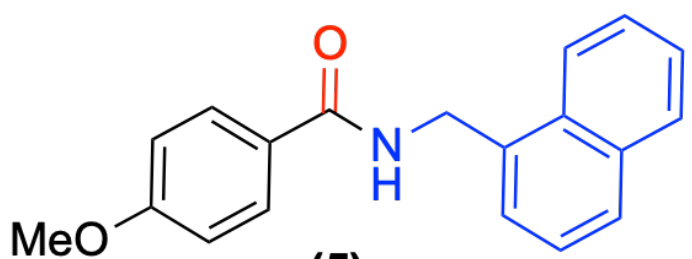

(5)

Reaction by Biphasic Cascade (Method A) gave a crude yield of 76% by ^{1}H NMR. Crude ^{1}H NMR was matched with values from the isolated compound synthesised below. ESI-MS (+, MeOH): m/z calculated for $C_{19}H_{17}NO_2 + H^+$: 292.1338. Found: 292.1339.

Reaction by General Batch Synthesis (Method B) yielded the isolated product after column chromatography (SiO_2 | Hexane:Et_2O, 7:3; Rf = 0.3) as a *crystalline white solid*. Yield: 64% (37.3 mg, 0.13 mmol)

^{1}H NMR (700 MHz, $CDCl_3$) δ (ppm): 8.08 (d, *J* = 8.3 Hz, 1H), 7.89 (d, *J* = 9.2 Hz, 1H), 7.83 (d, *J* = 8.2 Hz, 1H), 7.72 (d, *J* = 8.9 Hz, 2H), 7.57 – 7.49 (m, 3H), 7.44 (dd, *J* = 8.2, 6.9 Hz, 1H), 6.87 (d, *J* = 8.9 Hz, 2H), 6.31 (s, 1H), 5.07 (d, *J* = 5.3 Hz, 2H), 3.81 (s, 3H). **^{13}C NMR** (176 MHz, $CDCl_3$) δ (ppm): 166.80, 162.33, 134.05, 133.71, 131.65, 128.92, 128.91, 128.84, 127.02, 126.86, 126.67, 126.17, 125.55, 123.70, 113.86, 55.51, 42.49. **ESI-MS** (+, MeOH): m/z calculated for $C_{19}H_{17}NO_2$ + H^+: 292.1338. Found: 292.1339. **IR** (ATR): 2918, 2853, 1630, 1604, 1548, 1500, 1301, 1250, 1187, 1112, 1029 cm^{-1}. **UV-Vis** (EtOAc): λ_{max} 253, 271, 280, 292 nm. **m.p.** 175-177 °C.

4-methoxy-N-phenylbenzamide (**6**)

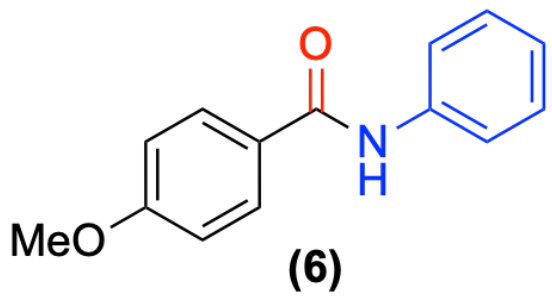

(6)

Reaction by Biphasic Cascade (Method A) with the following modification gave a crude yield of 36% by NMR. The reaction vial was placed under the light for 4 h at 25 °C before heating it at 80 °C for 24 h. Crude ^{1}H NMR was matched with isolated compounds in the literature.[10] ESI-MS (+, MeOH): m/z calculated for $C_{14}H_{13}O_2N$ + H^+: 228.1025. Found: 228.1026.

N-octyl-[1,1'-biphenyl]-4-carboxamide (**7**)

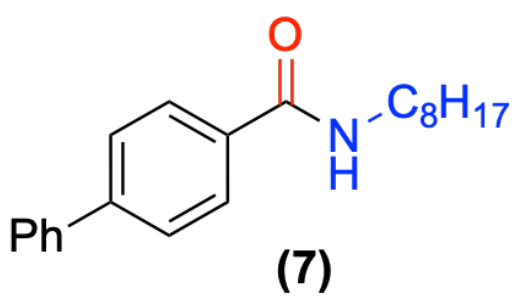

(7)

Reaction by Biphasic Cascade (Method A) gave a crude yield of 85% by ^{1}H NMR. Crude ^{1}H NMR was matched with isolated compounds in the literature.[11] ESI-MS (+, MeOH): m/z calculated for $C_{21}H_{27}NO$ + H^+: 310.2171. Found: 310.2161.

4-acetyl-N-octylbenzamide (**8**)

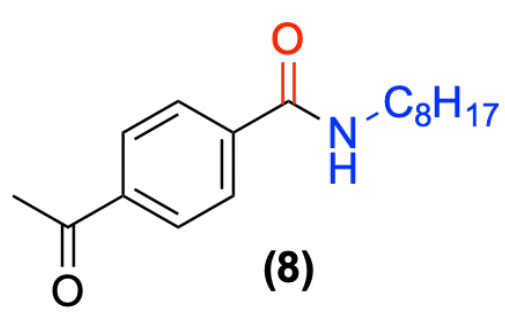

(8)

Reaction by Biphasic Cascade (Method A) gave a crude yield of 71% by NMR. Crude ^{1}H NMR was matched with values from the isolated compound synthesised below. ESI-MS (+, MeOH): m/z calculated for $C_{17}H_{25}NO_2$ + H^+: 276.1964. Found: 276.1962.

Reaction by General Batch Synthesis (Method B) yielded the isolated product after column chromatography (SiO_2 | Et_2O; Rf = 0.5) as a *crystalline white solid*. Yield: 77% (42.3 mg, 0.15 mmol).

^{1}H NMR (700 MHz, $CDCl_3$) δ (ppm): 7.99 (d, *J* = 8.5 Hz, 2H), 7.83 (d, *J* = 8.4 Hz, 2H), 6.22 (s, 1H), 3.46 (q, *J* = 6.6, 6.2 Hz, 2H), 2.63 (s, 3H), 1.65 – 1.59 (m, 2H), 1.41 – 1.35 (m, 2H), 1.35 – 1.23 (m, 8H), 0.87 (t, J = 6.9 Hz, 3H). **^{13}C NMR** (176 MHz, $CDCl_3$) δ (ppm): 197.58, 166.63, 139.19, 138.91, 128.65, 127.30, 40.43, 31.92, 29.74, 29.41, 29.33, 27.14, 26.94, 22.77, 14.22. **ESI-MS** (+, MeOH): m/z calculated for $C_{17}H_{25}NO_2$ + H^+: 276.1964. Found: 276.1964. **IR** (ATR): 3335, 2918, 2849, 1680, 1625, 1608, 1581, 1547, 1503, 1462, 1440, 1250, 1175, 1028 cm^{-1}. **UV-Vis** (EtOAc): λ_{max} 253 nm. **m.p.** 125 °C.

<u>4-cyano-N-octylbenzamide (**9**)</u>

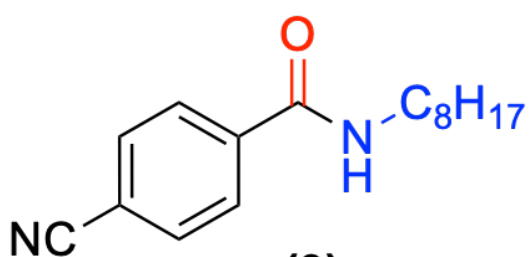

(9)

Reaction by Biphasic Cascade (Method A) gave a crude yield of 88% by NMR. Crude ^{1}H NMR was matched with values from the isolated compound synthesised below. ESI-MS (+, MeOH): m/z calculated for $C_{16}H_{22}ON_2$ + H^+: 259.1810. Found: 259.1810.

Reaction by General Batch Synthesis (Method B) yielded the isolated product after column chromatography (SiO_2 | Et_2O; Rf = 0.8) as a *crystalline red solid*. Yield: 90% (46 mg, 0.18 mmol).

^{1}H NMR (700 MHz, $CDCl_3$) δ (ppm): 7.86 (d, *J* = 8.6 Hz, 2H), 7.70 (d, *J* = 8.6 Hz, 2H), 6.47 (s, 1H), 3.42 (td, *J* = 7.3, 5.7 Hz, 2H), 1.63 – 1.56 (m, 2H), 1.38 – 1.32 (m, 2H), 1.31 – 1.23 (m, 8H), 0.86 (t, *J* = 7.1 Hz, 3H). **^{13}C NMR** (176 MHz, $CDCl_3$) δ (ppm): 165.82, 138.89, 132.46, 127.75, 118.14, 114.93, 40.50, 31.86, 29.61, 29.35, 29.28, 27.08, 22.72, 14.17. **ESI-MS** (+, MeOH): m/z calculated for $C_{16}H_{22}ON_2$ + H^+: 259.1810. Found: 259.1805. **IR** (ATR): 3348, 2954, 2918, 2848, 1638, 1537, 1501, 1464, 1348, 1316, 1281 cm^{-1}. **UV-Vis** (EtOAc): λ_{max} 252 nm. **m.p.** 83 °C.

Methyl 4-(octylcarbamoyl)benzoate (**10**)

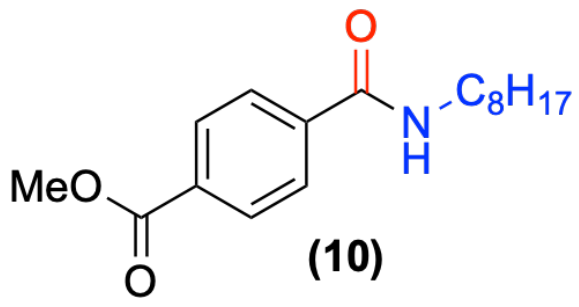

(10)

Reaction by Biphasic Cascade (Method A) gave a crude yield of 94% by NMR. Crude ^{1}H NMR was matched with values from the isolated compound synthesised below. ESI-MS (−, MeOH): m/z calculated for $C_{17}H_{25}NO_3 - H^+$: 290.1756. Found: 290.1759.

Reaction by General Batch Synthesis (Method B) yielded the isolated product after column chromatography (SiO_2 | Et_2O; Rf = 0.6) as a *crystalline pale red solid*. Yield: 64% (37.3 mg, 0.13 mmol).

^{1}H NMR (700 MHz, $CDCl_3$) δ (ppm): 8.04 (d, *J* = 8.5 Hz, 2H), 7.80 (d, *J* = 8.5 Hz, 2H), 6.44 (s, 1H), 3.91 (s, 3H), 3.42 (q, *J* = 6.6, 6.1 Hz, 2H), 1.59 (m, *J* = 7.4 Hz, 2H), 1.35 (p, *J* = 6.5 Hz, 2H), 1.32 – 1.21 (m, 8H), 0.86 (t, *J* = 7.0 Hz, 3H). **^{13}C NMR** (176 MHz, $CDCl_3$) δ (ppm): 166.77, 166.43, 138.90, 132.58, 129.86, 127.04, 52.46, 40.40, 31.89, 29.70, 29.38, 29.30, 27.11, 22.73, 14.18. **ESI-MS** (+, MeOH): m/z calculated for $C_{17}H_{25}NO_3 + H^+$: 292.1908. Found: 292.1917. **IR** (ATR): 3319, 2957, 2918, 2850, 1720, 1637, 1574, 1537, 1498, 1467, 1435, 1278, 1194, 1149, 1110 cm^{-1}. **UV-Vis** (EtOAc): λ_{max} 252 nm. **m.p.** 115-117 °C.

4-nitro-N-octylbenzamide (**11**)

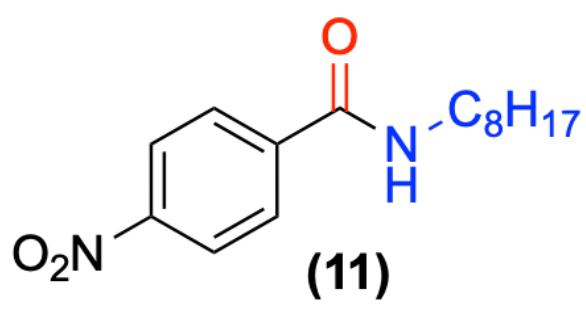

(11)

Reaction by Biphasic Cascade (Method A) gave a crude yield of 50% by NMR. Crude ^{1}H NMR was matched with values from the isolated compound synthesised below. ESI-MS (−, MeOH): m/z calculated for $C_{15}H_{22}N_2O_3 - H^+$: 277.1552. Found: 277.1554.

Reaction by General Batch Synthesis (Method B) yielded the isolated product after column chromatography (SiO_2 | Hexane:Et_2O, 1:1; Rf = 0.3) as a *crystalline pale red solid*. Yield: 94% (50 mg, 0.18 mmol).

^{1}H NMR (700 MHz, $CDCl_3$) δ (ppm): 8.26 (d, J = 8.9 Hz, 2H), 7.92 (d, J = 8.9 Hz, 2H), 6.32 (s, 1H), 3.48 – 3.43 (m, 2H), 1.62 (p, J = 7.5 Hz, 2H), 1.40 – 1.33 (m, 2H), 1.33 – 1.24 (m, 8H), 0.87 (t, J = 7.1 Hz, 3H). **^{13}C NMR** (176 MHz, $CDCl_3$) δ (ppm): 165.59, 149.61, 140.56, 128.19, 123.91, 40.61, 31.90, 29.66, 29.38, 29.31, 27.11, 22.76, 14.21. **ESI-MS** (+, MeOH): m/z calculated for $C_{15}H_{22}N_2O_3$ + H^+: 279.1709. Found: 279.1717. **IR** (ATR): 3304, 2921, 2853, 1637, 1596, 1537, 1511, 1343, 1285 cm^{-1}. **UV-Vis** (EtOAc): λ_{max} 264 nm. **m.p.** 93-95 °C.

N-octyl-4-(trifluoromethyl)benzamide (**12**)

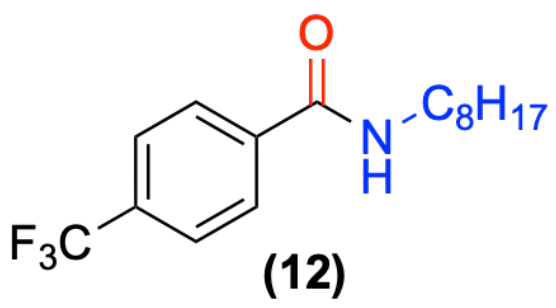

(12)

Reaction by Biphasic Cascade (Method A) gave a crude yield of 47% by NMR. Crude ^{1}H NMR was matched with values from the isolated compound synthesised below. ESI-MS (+, MeOH): m/z calculated for $C_{16}H_{22}NOF_3$ + H^+: 302.1732. Found: 302.1744.

Reaction by General Batch Synthesis (Method B) yielded the isolated product after column chromatography (SiO_2 | Hexane:Et_2O, 7:3; Rf = 0.6) as a *crystalline pale red solid*.[12] Yield: 65% (39 mg, 0.13 mmol).

^{1}H NMR (700 MHz, $CDCl_3$) δ (ppm): 7.85 (d, J = 8.1 Hz, 2H), 7.66 (d, J = 8.2 Hz, 2H), 6.38 (s, 1H), 3.44 (td, J = 7.3, 5.7 Hz, 2H), 1.65 – 1.56 (m, 2H), 1.36 (dq, J = 8.9, 6.6, 6.1 Hz, 2H), 1.32 – 1.22 (m, 8H), 0.88 (s, 3H). **^{13}C NMR** (176 MHz, $CDCl_3$) δ (ppm): 166.40, 138.27, 133.04 (q, $J_{F\text{-}C}$ = 32.6 Hz), 127.47, 125.55 (q, $J_{F\text{-}C}$ = 3.8 Hz), 123.67 (d, $J_{F\text{-}C}$ = 272.6 Hz), 40.46, 31.91, 29.71, 29.40, 29.33, 27.13, 22.76, 14.19. ^{19}F NMR (400 MHz, $CDCl_3$) δ (ppm): -62.88 (s). **ESI-MS** (+, MeOH): m/z calculated for $C_{16}H_{22}NOF_3$ + H^+: 302.1732. Found: 302.1741. **IR** (ATR): 3333, 2918, 2850, 1634, 1580, 1535, 1511, 1469, 1406, 1331, 1166, 1126, 1071, 1018 cm^{-1}. **UV-Vis** (EtOAc): λ_{max} 254 nm. **m.p.** 64-66 °C.

1,3-bis(4-methoxyphenyl)prop-2-yn-1-one (**13**)

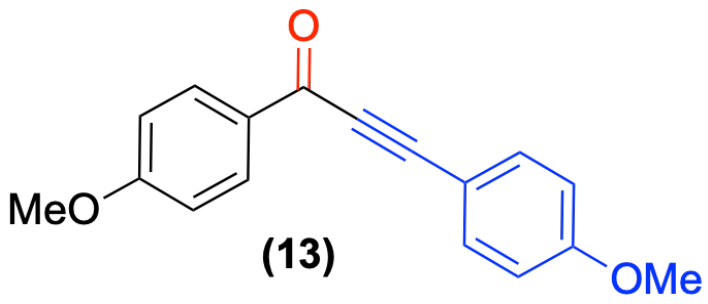

(13)

Reaction by Biphasic Cascade (Method A) with the following modification gave a crude NMR yield of 40% and 68% with RuPS/CoCat1 and CuPS/CoCat2 systems, respectively. CuI (20 mol%) and trioctylamine (0.06 mmol, 3 equiv.) were added in the organic reaction with Ar-I (0.02 mmol, 1 equiv.), $Pd(OAc)_2$ (5 mol%) and Xantphos (10 mol%) in dry toluene (2 mL) under N_2, prior to the addition of Nu (0.1 mmol, 5 equiv.), and the reaction vial was illuminated for 4 h at 25 °C before heating it at 80 °C for 72 h. Crude ^1H NMR was matched with isolated compounds in the literature.[13,14] ESI-MS (+, MeOH): m/z calculated for $C_{17}H_{14}O_3 + H^+$: 267.1021. Found: 267.1006.

1-(4-(4-(*tert*-butyl)benzoyl)phenyl)ethan-1-one (**14**)

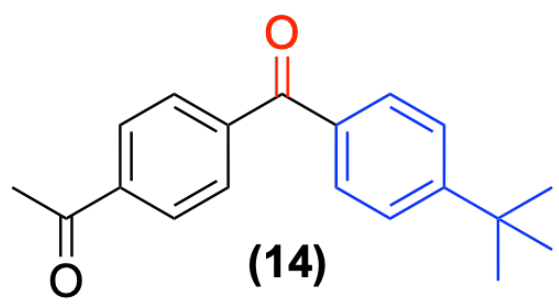

(14)

Reaction by Biphasic Cascade (Method A) with the following modification gave a crude yield of 47% and 31% with RuPS/CoCat1 and CuPS/CoCat2 systems, respectively, by NMR. The organic layer was prepared by dissolving Ar-I (0.02 mmol, 1 equiv.), $Pd(OAc)_2$ (5 mol%) and CatacXium·HI (10 mol%) in dry 4:1 Toluene:1,2-difluorobenzene under N_2, prior to the addition of Nu (0.1 mmol, 5 equiv.). The reaction vial was illuminated for 4 h at 25 °C before heating it at 80 °C for 24 h. Crude ^1H NMR was matched with isolated compounds in the literature.[17] ESI-MS (+, MeOH): m/z calculated for $C_{19}H_{20}O_2 + H^+$: 281.1542. Found: 281.1543.

N-(4-(2-(dimethylamino)ethoxy)benzyl)-3,4-dimethoxybenzamide (**15**)

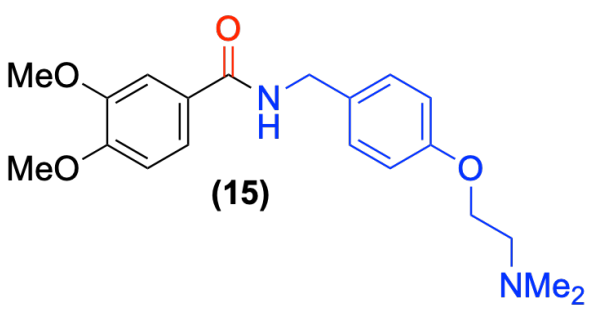

(15)

Reaction by Biphasic Cascade (Method A) with the following modification gave a crude yield of 50% and 20% with RuPS/CoCat1 and CuPS/CoCat2 systems, respectively, by NMR. Nu (0.03 mmol, 1.5 equiv.) was used in this case. Trioctylamine (0.06 mmol, 3 equiv.) was added in the organic reaction with the aforementioned reagents, and the reaction vial was illuminated for 4 h at 25 °C before heating it at 80 °C for 24 h. Crude ^{1}H NMR was matched with isolated compounds in the literature.[18] ESI-MS (+, MeOH): m/z calculated for $C_{20}H_{26}N_2O_4 + H^+$: 359.1971. Found: 359.1985.

Same reaction repeated in dry toluene with 2.5 equiv. injected CO yields 60% of product formation. Crude ^{1}H NMR was matched with isolated compounds in the literature.[18]

Supplementary Tables

Table S1. Tabulated values from photocatalytic experiments and exclusion controls.[a]

Misc	Volume (mL)	Cat (µM)	PS (µM)	Red (mM)	Buffer (mM)	CO (µmol)		TON_{CO}		TOF_{CO} (h^{-1})		Sel% $CO:H_2$	
						Mean	SD	Mean	SD	Mean	SD	Mean	SD
All	6	10	500	100	100	73.5	0.2	1225	3	51.1	0.1	83.0	<0.1
All	2	0.1	500	100	100	1.4	0.1	7033	253	293	11	15.0	0.1
All	2	1	500	100	100	3.0	0.1	1520	39	63	2	29.0	0.7
All	2	5	500	100	100	13.0	0.9	1283	89	53	4	69.0	1.2
All	2	30	500	100	100	37.6	-	627	-	26	-	89	-
All	2	50	500	100	100	51	1	510	10	21.2	0.4	88.0	0.6
All	2	10	10	100	100	0.9	-	46.4	-	1.9	-	83.0	-
All	2	10	100	100	100	6.0	0.2	302	10	13.0	0.4	75.0	0.3
All	2	10	250	100	100	24.3	0.4	1216	20	51.0	0.8	83.0	0.1
All	2	10	500	100	100	22.6	0.2	1129	11	47.0	0.0	84.0	0.3
All	4	10	500	100	100	35	8	1772	406	74	17	82	6
All	8	10	500	100	100	83	1	1382	18	58.0	0.7	81.0	0.7
No Cat	2	0	500	100	100	1.3	-	-	-	-	-	12.0	-
No PS	2	10	0	100	100	0.0	-	-	-	-	-	-	-
No Red	2	10	500	0	100	0.0	-	-	-	-	-	-	-
No Buffer	2	10	500	100	0	13.9	-	696	-	29	-	54	-
No CO_2 nor Buffer	2	10	500	100	0	4.5	-	253	-	11	-	52	-
No light	2	10	500	100	100	0.0	-	-	-	-	-	-	-
0 h	6	10	500	100	100	0.0	-	-	-	-	-	-	-
2 h	6	10	500	100	100	16.8	0.1	280	2	140.0	0.1	78.0	0.9
4 h	6	10	500	100	100	33.7	0.2	561	4	140.3	0.2	81.0	<0.1
8 h	6	10	500	100	100	54.8	0.6	913	11	114.1	0.4	82.0	<0.1
16 h	6	10	500	100	100	69.6	0.0	1160	0.4	72.5	0.0	83.0	<0.1
24 h	6	10	500	100	100	73.5	0.2	1225	3	51.1	0.1	83.0	<0.1
48 h[b]	6	10	500	100	100	69	17	1149	17	24.0	0.7	83.0	<0.1
0 h with Toluene[c]	6	10	500	100	100	0.0	-	-	-	-	-	-	-

2 h with Toluene[c]	6	10	500	100	100	11.9	0.3	228	5	114.2	0.2	71.0	0.2
4 h with Toluene[c]	6	10	500	100	100	21	1	412	21	103.1	0.9	74.0	0.2
8 h with Toluene[c]	6	10	500	100	100	33	2	635	40	79.4	2	75.0	0.2
16 h with Toluene[c]	6	10	500	100	100	41	4	796	17	49.8	3	76.0	0.3
24 h with Toluene[c]	6	10	500	100	100	40	4	765	72	31.9	3	75.0	0.3
48 h with Toluene[c]	6	10	500	100	100	40	4	724	71	15.1	3	73.0	0.4

[a] Cat = CoCat1, PS = RuPS, Red. = NaHAsc, Buffer. = $NaHCO_3$. Reaction conditions: CO_2-sat. aqueous solutions (pH 6.8) sampled after 24 h LED illumination (447 nm, 2.3 W) at 25 °C with 250 rpm orbital shaking. Reactions in duplicate, errors given as ± 1 standard deviation with values in the main text and plots using a minimum error value of 5%. Quantification by GC. [b] Different reaction batch compared to 2-24 h datapoints in the set. [c] 2 mL of N_2-sat. toluene added on top of aqueous layer.

Table S2. Tabulated values to measure dissolved CO in the toluene layer.[a]

Misc	CO in Headspace (µmol)	
	Mean	SD
H_2O	51.1	-
Aqueous $NaHCO_3$	51.0	0.8
Toluene on Aqueous $NaHCO_3$	42.7	0.5

[a] GC headspace analysis of ambient pressure equilibrated vials containing water (air sat., 6 mL); aqueous $NaHCO_3$ (CO_2-sat., pH 6.8, 6 mL); or biphasic with aqueous $NaHCO_3$ (CO_2-sat., pH 6.8, 6 mL) and toluene (N_2-sat., 2 mL). Measured in duplicate with errors given as ± 1 standard deviation. 50 µmol of exogenous CO was injected into the headspace above the solutions.

Table S3. Tabulated values from photocatalytic experiments with different systems.[a]

System	Cat (µM)	PS (µM)	Red (mM)	Buffer (mM)	Yield CO (µmol)		TON_{CO}		TOF_{CO}		Sel% CO:H2	
					Mean	SD	Mean	SD	Mean	SD	Mean	SD
RuPS/CoCat1	10	500	100	100	73.5	0.2	1225	3	51.1	0.1	83.0	<0.1
RuPS/CoCat2	10	500	100	100	15	5	258	77	11	3	45	12
CuPS/CoCat1	10	500	100	100	35	-	584	-	24	-	89	-
CuPS/CoCat2	10	500	100	100	105	3	1742	49	73	2	81.0	0.4

[a] Red. = NaHAsc, Buffer. = $NaHCO_3$. Reaction conditions: CO_2-sat. aqueous solutions (pH 6.8, 6 mL) sampled after 24 h LED illumination (447 nm for RuPS or 405 nm for CuPS, 2.3 W) at 25 °C with 250 rpm orbital shaking. Reactions in duplicate, errors given as ± 1 standard deviation with values in the main text and plots using a minimum error value of 5%. Quantification by GC.

Table S4. Tabulated values from carbonylation in a biphasic system with injected CO.[a]

Misc	CO (µmol)		Aryl Iodide 1 (µmol)		Benzamide 3 (µmol)	
	Mean	SD	Mean	SD	Mean	SD
0 h	48	1	15.0	0.1	0.0	0
2 h	45.6	0.6	13.2	0.2	2.5	0.2
4 h	44.8	0.6	11.8	0.5	3.6	0.5
8 h	43.7	0.6	10.4	0.1	5	1
16 h	40.9	0.5	8	1	8	2
24 h	38	1	5	1	11	3
48 h[b]	18	3	0.0	0.0	21	5

[a] Biphasic reaction with organic layer of iodoanisole (**1**, 0.02 mmol, 1 equiv.), octylamine (**2**, 100 µmol, 5 equiv.), $Pd(OAc)_2$ (5 mol%), Xantphos (10 mol%) in toluene (N_2 sat., 2 mL) on aqueous layer of $NaHCO_3$ (100 mM) in H_2O (CO_2 sat., pH 6.8, 6 mL) stirred at 25 °C for 24 h. Quantification by GC (gases) and GC-MS (liquids). Reactions in duplicate, errors given as ± 1 standard deviation with values in the main text and plots using a minimum error value of 5%. [b] Different batch of reaction.

Table S5. Optimisation table for carbonylation reaction coupled with photocatalysis using Ru system.[a]

Entry	2 (equiv.)	Pd (mol%)	Xantphos (mol%)	Yield of (3) (%)
1[b]	1.5	2	2	5
2	3	2	2	13
3	5	2	2	9
4	10	2	2	24
5	100	2	2	n.d.
6	3	5	5	11
7[c]	3	10	10	39
8	3	5	10	22
9	3	2	10	13
10	3	10	10	55
11	3	10	20	73
12	5	10	10	80
13	5	10	20	87
14	10	10	20	94
15	5	5	10	71
16	5	5	20	60
17[c]	5	5	10	21
18	1.5	10	10	30

[a] Biphasic reaction with organic layer of iodoanisole (**1**, 0.02 mmol, 1 equiv.), octylamine (**2**), $Pd(OAc)_2$, Xantphos in toluene (N_2 sat., 2 mL); aqueous layer: CoCat1 (10 µM), RuPS (500 µM), NaHAsc (100 mM), $NaHCO_3$ (100 mM) in H_2O (CO_2 sat., pH 6.8, 6 mL); illumination from blue LED (447 nm, 2.3 W), 25 °C,

250 rpm orbital shaking. Yields determined by ^{1}H NMR by integration using 1,3,5-trimethoxybenzene as an internal standard, n.d. = none detected. [b] DABCO = 3 equiv. [c] organic layer purged with CO_2.

Table S6. Ligand screening for Pd for carbonylation reaction under biphasic conditions.[a]

Entry	2 (equiv.)	Pd (mol%)	Ligand (mol%)	Yield % (3)
1	5	5	L_1 (7.5)	73
2	5	5	L_2 (7.5)	17
3	5	5	L_3 (7.5)	n.d.
4	5	5	L_4 (15)	3
5	5	5	L_5 (15)	n.d.
6	5	5	L_6 (15)	n.d.
7	5	5	L_7 (15)	n.d.
8	5	5	L_8 (15)	n.d.

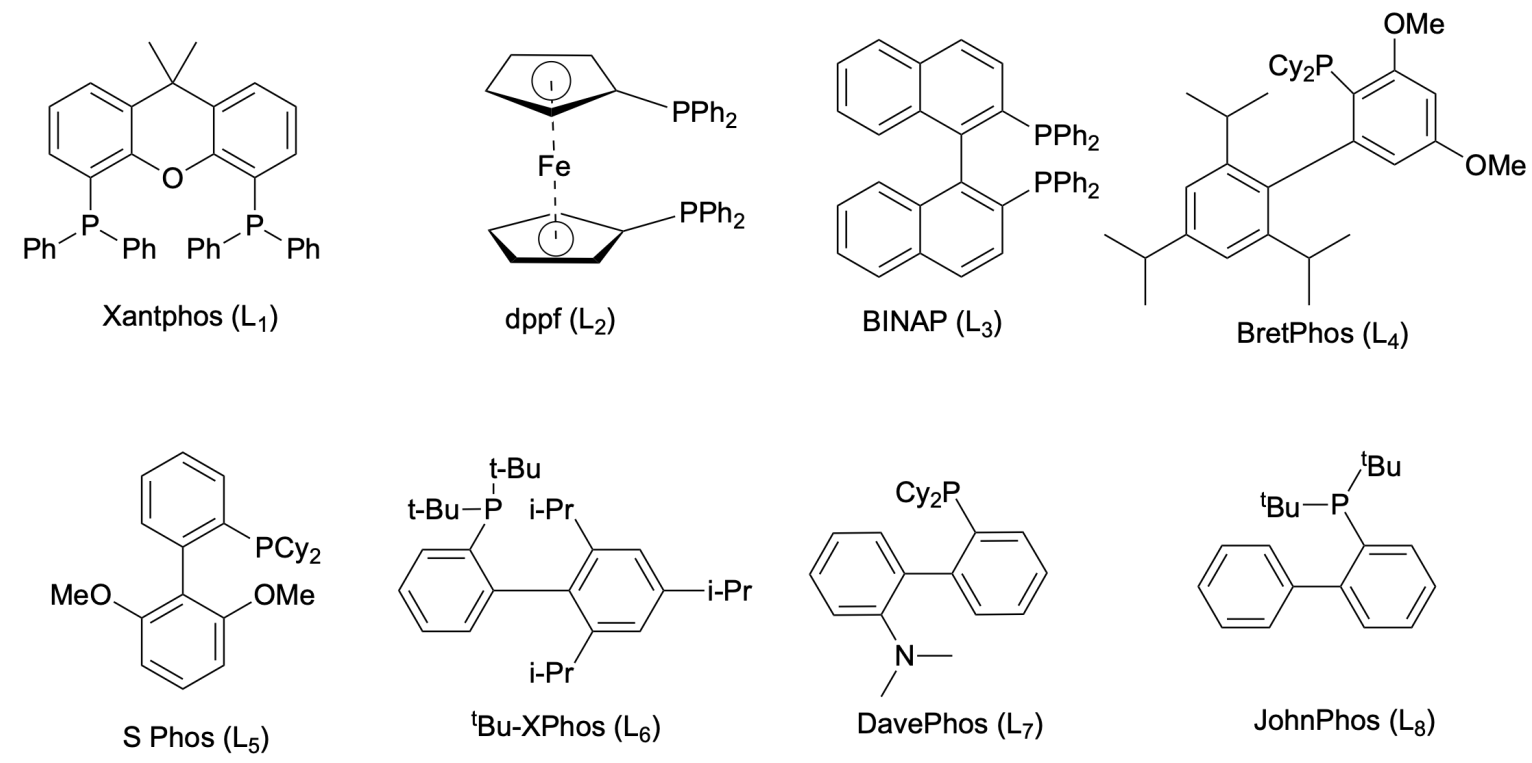

[a] Biphasic reaction with organic layer of iodoanisole (**1**, 0.02 mmol, 1 equiv.), octylamine (**2**, 100 µmol, 5 equiv.), $Pd(OAc)_2$ (5 mol%), ligand in toluene (N_2 sat., 2 mL) on aqueous layer of $NaHCO_3$ (100 mM) in H_2O (CO_2 sat., pH 6.8, 6 mL) stirred at 25 °C for 24 h. Yields determined by ^{1}H NMR by integration using 1,3,5-trimethoxybenzene as an internal standard, n.d. = none detected.

Table S7. Tabulated values from photocatalytic experiments coupled with carbonylation in a biphasic system.[a]

Misc	Cat (µM)	PS (µM)	Red (mM)	Buffer (mM)	CO (µmol)		TON_{CO}		Ar-I (1) (µmol)		Benzamide (3) (µmol)	
					Mean	SD	Mean	SD	Mean	SD	Mean	SD
0 h	10	500	100	100	0.0	0.0	0.0	0.0	16	2	0.0	0.0
2 h	10	500	100	100	4.6	0.8	77	12	14	2	1.9	0.6
4 h	10	500	100	100	9	1	148	21	13	1	2.6	0.2
8 h	10	500	100	100	14	2	226	30	13	2	2.8	0.0
16 h	10	500	100	100	17	2	279	39	10.4	0.6	4.6	0.4
24 h	10	500	100	100	14	3	230	46	7.3	0.3	8.0	0.8
48 h[b]	10	500	100	100	20	3	246	40	0.0	0.0	18	2

[a] Biphasic reaction with organic layer of iodoanisole (**1**, 0.02 mmol, 1 equiv.), octylamine (**2**, 100 µmol, 5 equiv.), $Pd(OAc)_2$ (5 mol%), Xantphos (10 mol%) in toluene (N_2 sat., 2 mL) on aqueous layer: CoCat1 (10 µM), RuPS (500 µM), NaHAsc (100 mM), $NaHCO_3$ (100 mM) in H_2O (CO_2 sat., pH 6.8, 6 mL); illumination from blue LED (447 nm, 2.3 W), 25 °C, 250 rpm orbital shaking. Quantification by GC (gases) and GC-MS (liquids). Reactions in duplicate, errors given as ± 1 standard deviation with values in the main text plots shown with a minimum error value of 5%. [b] Different batch of reaction.

Supplementary Figures

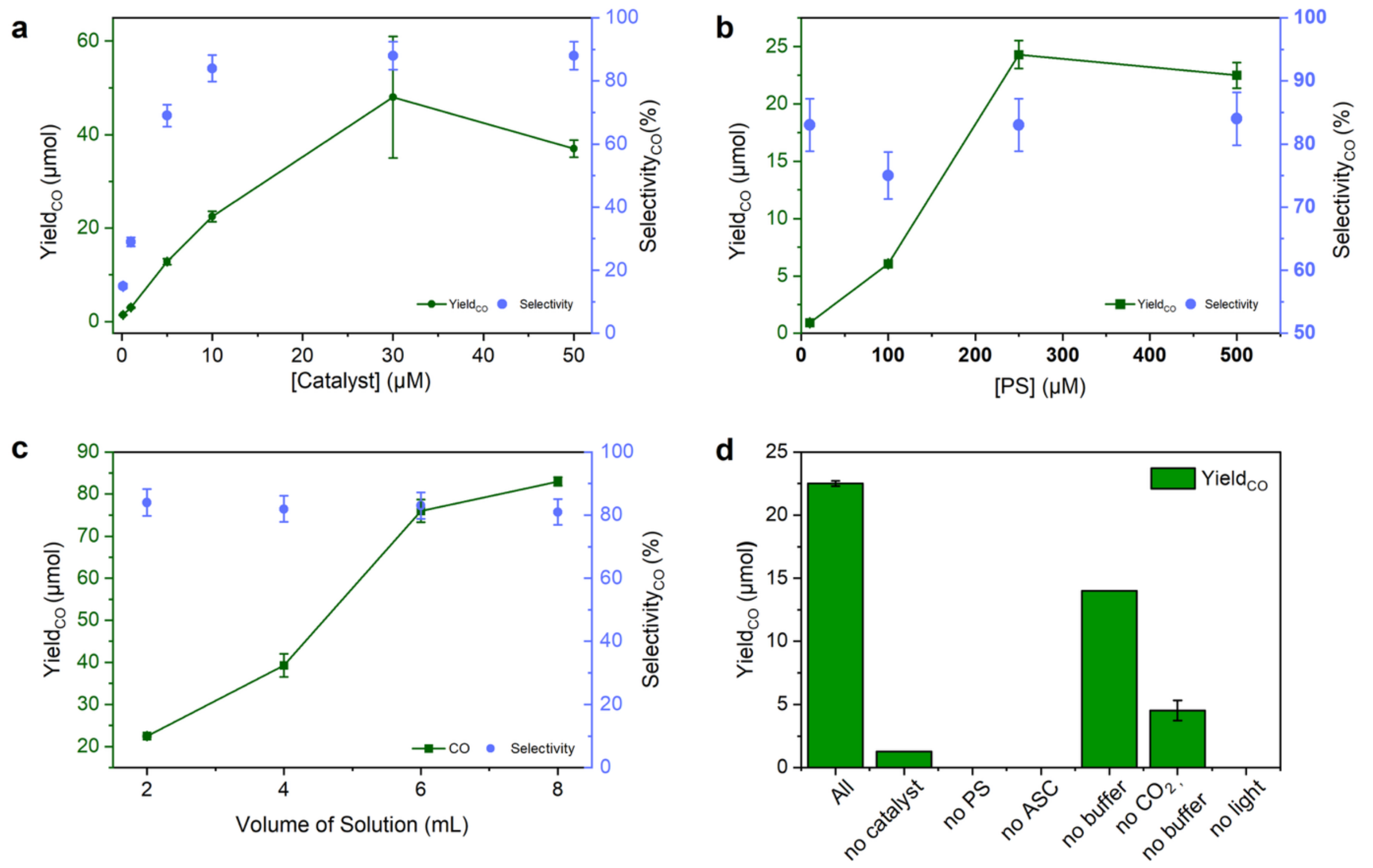

Figure S1. Photocatalytic system optimisation showing Yield$_{CO}$ and CO vs H_2 selectivity as a function of catalyst (**a**) and photosensitiser (**b**) solution volume (**c**) and exclusion controls (**d**). Unless specified, CoCat1 (10 µM), RuPS (500 µM), NaHAsc (100 mM), $NaHCO_3$ (100 mM) in H_2O (CO_2 sat., pH 6.8, 2 mL); 24 h LED illumination (447 nm for RuPS or 405 nm for CuPS, 2.3 W) at 25 °C with 250 rpm orbital shaking. Reactions in duplicate, errors plotted as ± 1 standard deviation with a minimum value of 5%. Quantification by GC.

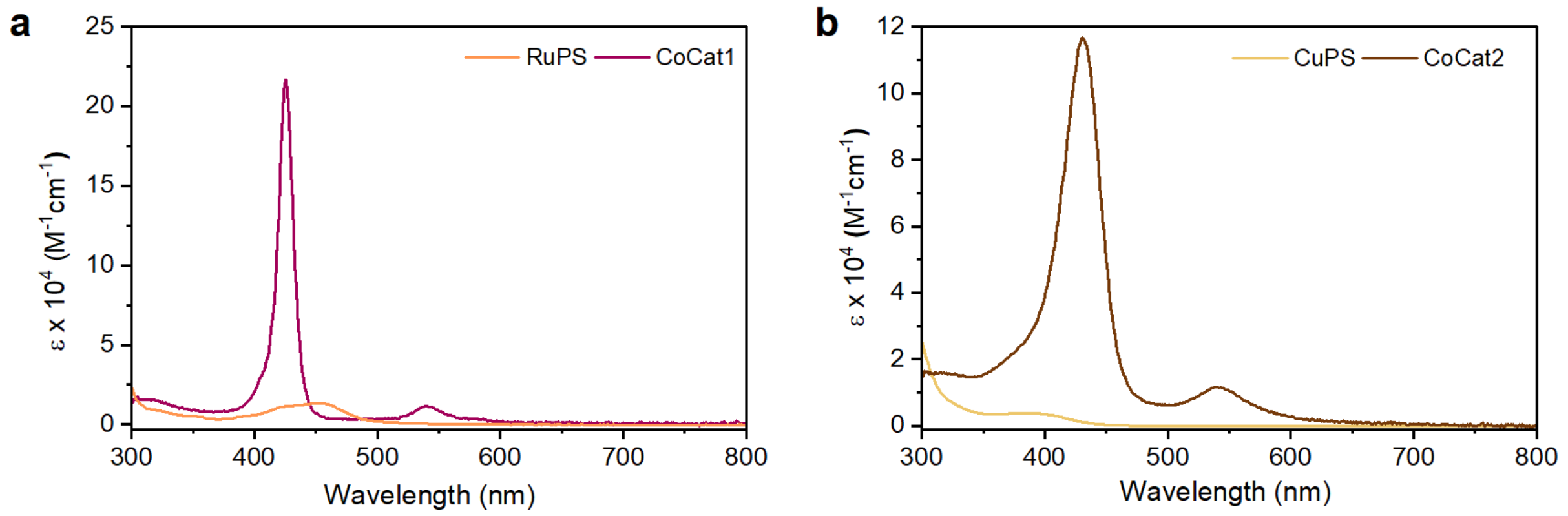

Figure S2. UV-Visible absorption spectra of (**a**) RuPS/CoCat1 in H_2O; and (**b**) CuPS/CoCat2 in H_2O.

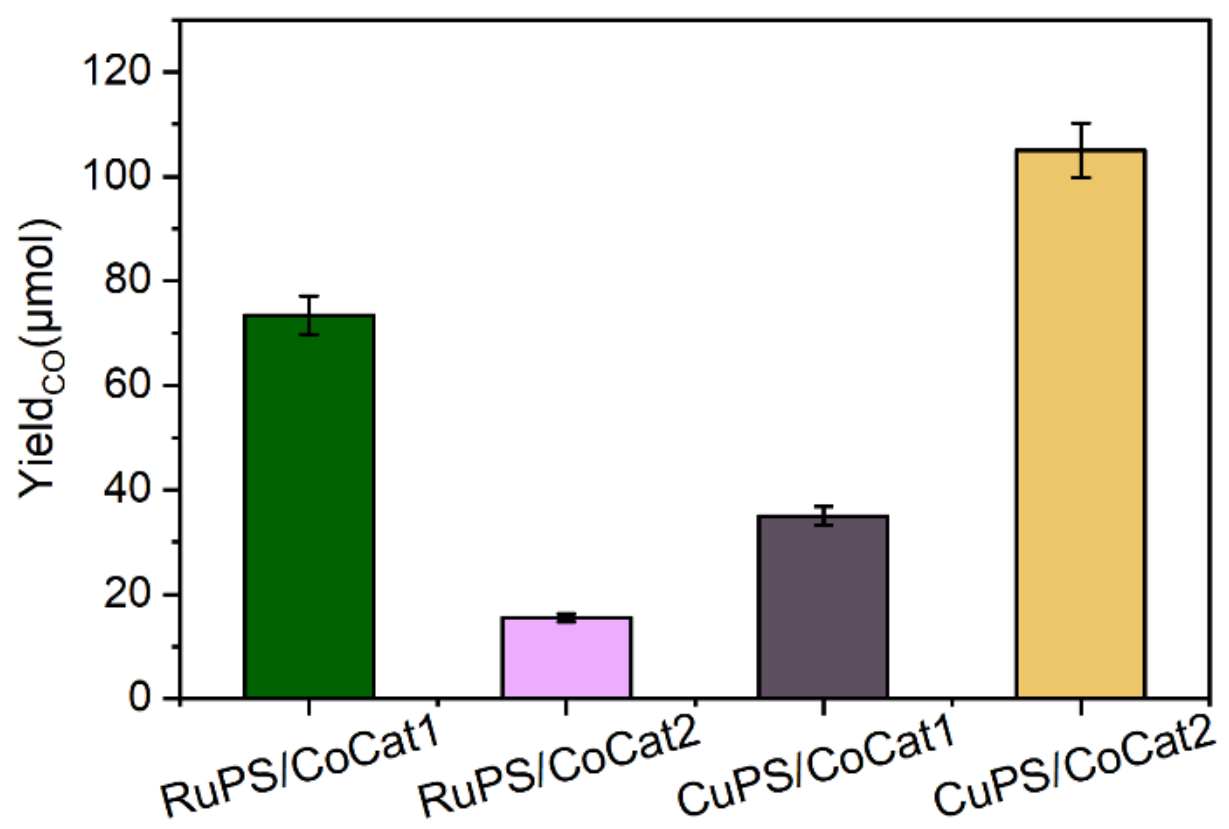

Figure S3. Comparison of CuPS, RuPS, CoCat1 and CoCat2 combinations for photocatalysis. Reaction conditions: CoCat1/CoCat2 (10 µM), RuPS/CuPS (500 µM), NaHAsc (100 mM), $NaHCO_3$ (100 mM) in H_2O (CO_2 sat., pH 6.8, 6 mL); 24 h LED illumination (447 nm for RuPS or 405 nm for CuPS, 2.3 W) at 25 °C with 250 rpm orbital shaking. Reactions in duplicate, errors plotted as ± 1 standard deviation. Quantification by GC.

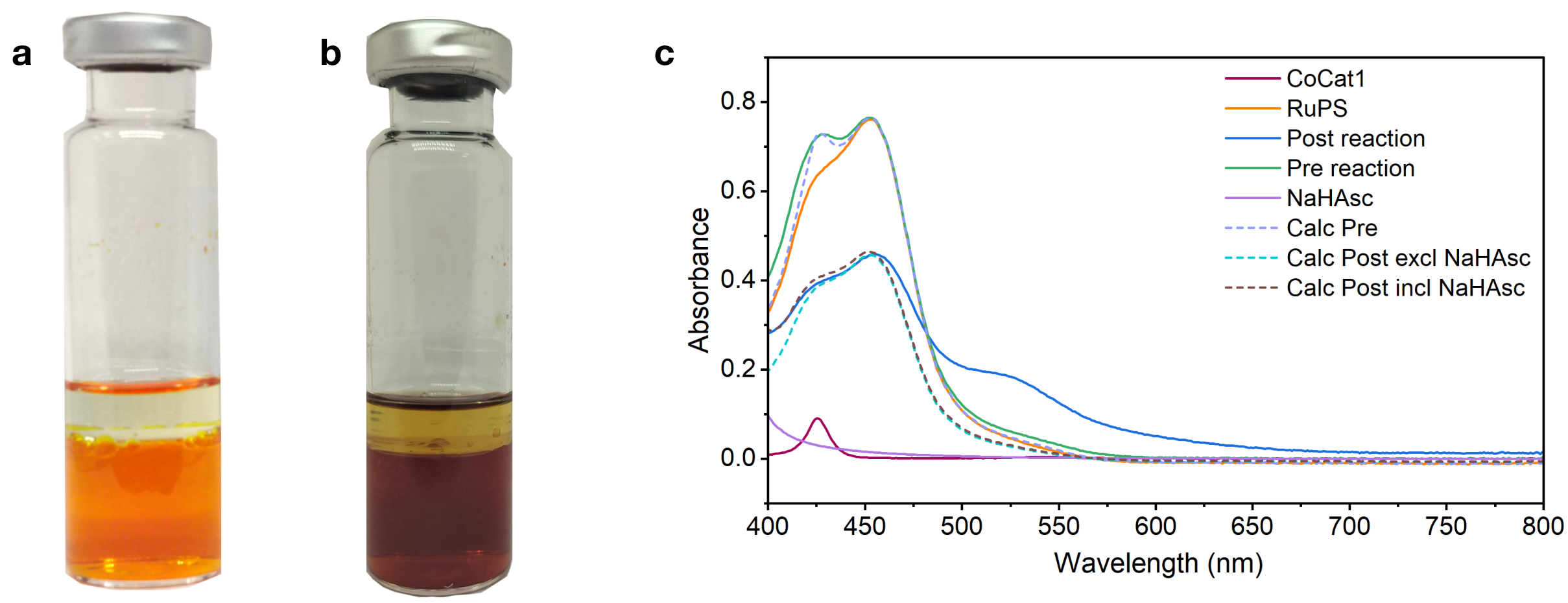

Figure S4. Photographs of the same reaction vial (**a**) before and (**b**) after 24 h photocatalysis and (**c**) corresponding UV-Vis spectra of the aqueous layer. Linear combination fitting of the UV-Vis spectra utilised the following coefficients: Calc Pre = RuPS + CoCat1, Calc post excl. NaHAsc = RuPS ×0.6 + CoCat1×0.05, Calc post incl. NaHAsc = RuPS ×0.6 + CoCat1×0 + NaHAsc (intensity normalised spectrum of ascorbate oxidised in air). Reaction conditions: organic layer: iodoanisole (20 µmol, 1 equiv.), octylamine (100 µmol, 5 equiv.), $Pd(OAc)_2$ (5 mol%), Xantphos (10 mol%), toluene (N_2 sat., 2 mL); aqueous layer: CoCat1 (10 µM), RuPS (500 µM), NaHAsc (100 mM), $NaHCO_3$ (100 mM) in H_2O (CO_2 sat., pH 6.8, 6 mL); 24 h illumination from blue LED (447 nm, 2.3 W), 25 °C, 250 rpm orbital shaking.

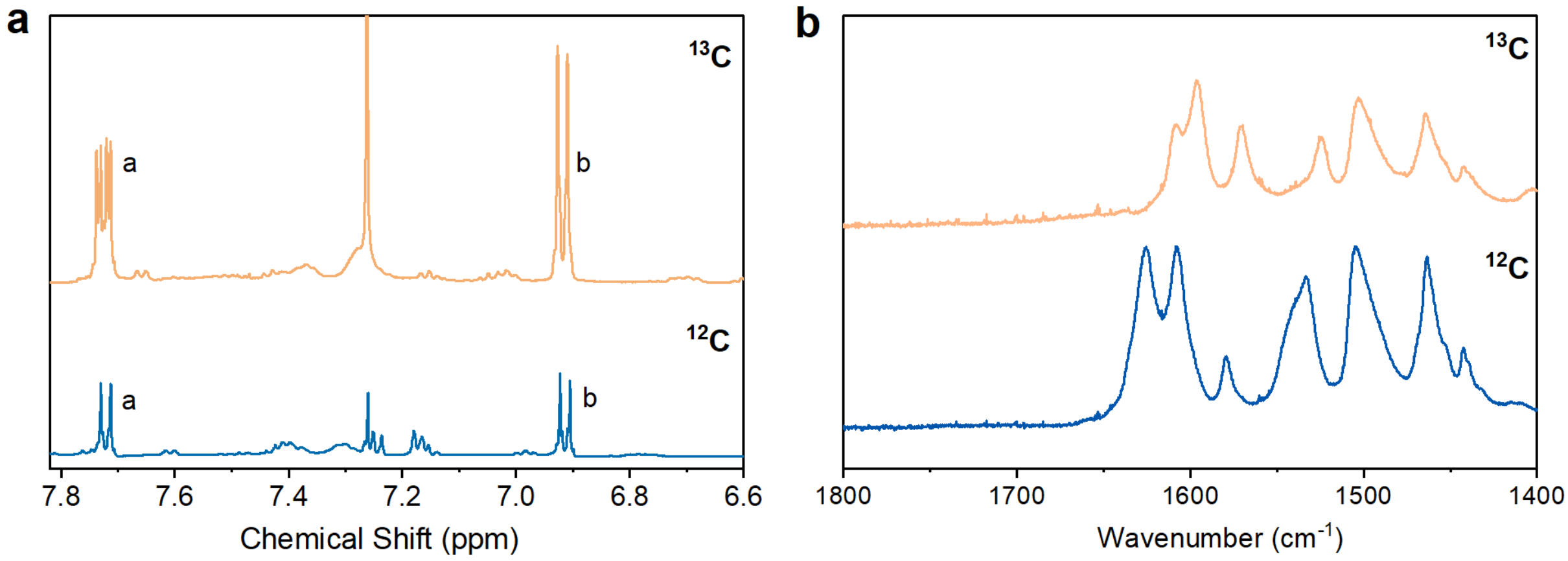

Figure S5. (**a**) Isotopic labelling using $^{12}CO_2$ or $^{13}CO_2$ with analysis of the crude benzamide product by ^{1}H NMR (500 MHz). (**b**) IR analysis of crude $^{13}CO_2$ reaction compared with the $^{12}CO_2$ sample. Reaction conditions: organic layer: iodoanisole (20 µmol, 1 equiv.), octylamine (100 µmol, 5 equiv), $Pd(OAc)_2$ (5 mol%), Xantphos (10 mol%), toluene (N_2 sat., 2 mL); aqueous layer: CoCat1 (10 µM), RuPS (500 µM), NaHAsc (100 mM), $NaHCO_3$ (100mM) in H_2O (CO_2 sat., pH 6.8, 6 mL); 24 h illumination from blue LED (447 nm, 2.3 W), 25 °C, 250 rpm orbital shaking.

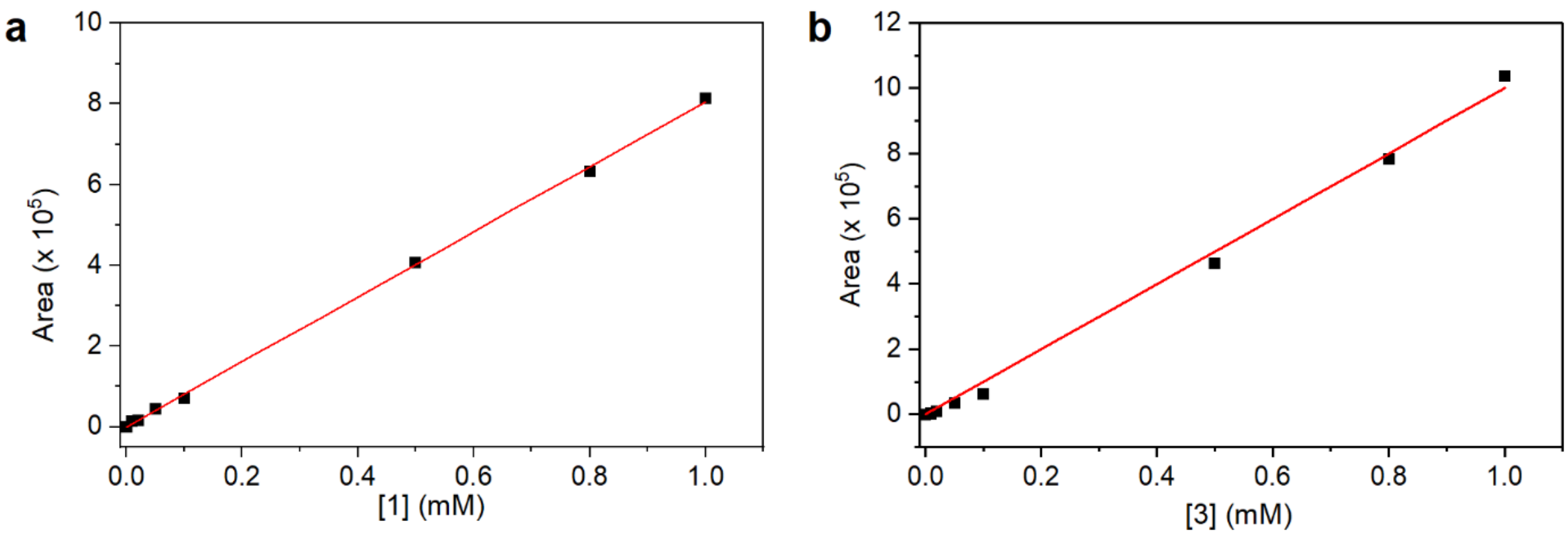

Figure S6. GC-MS calibration of **(a)** 4-Iodoanisole (**1**); **(b)** Benzamide product (**3**).

Molecular Characterisation of New Compounds (^{1}H and ^{13}C NMR)

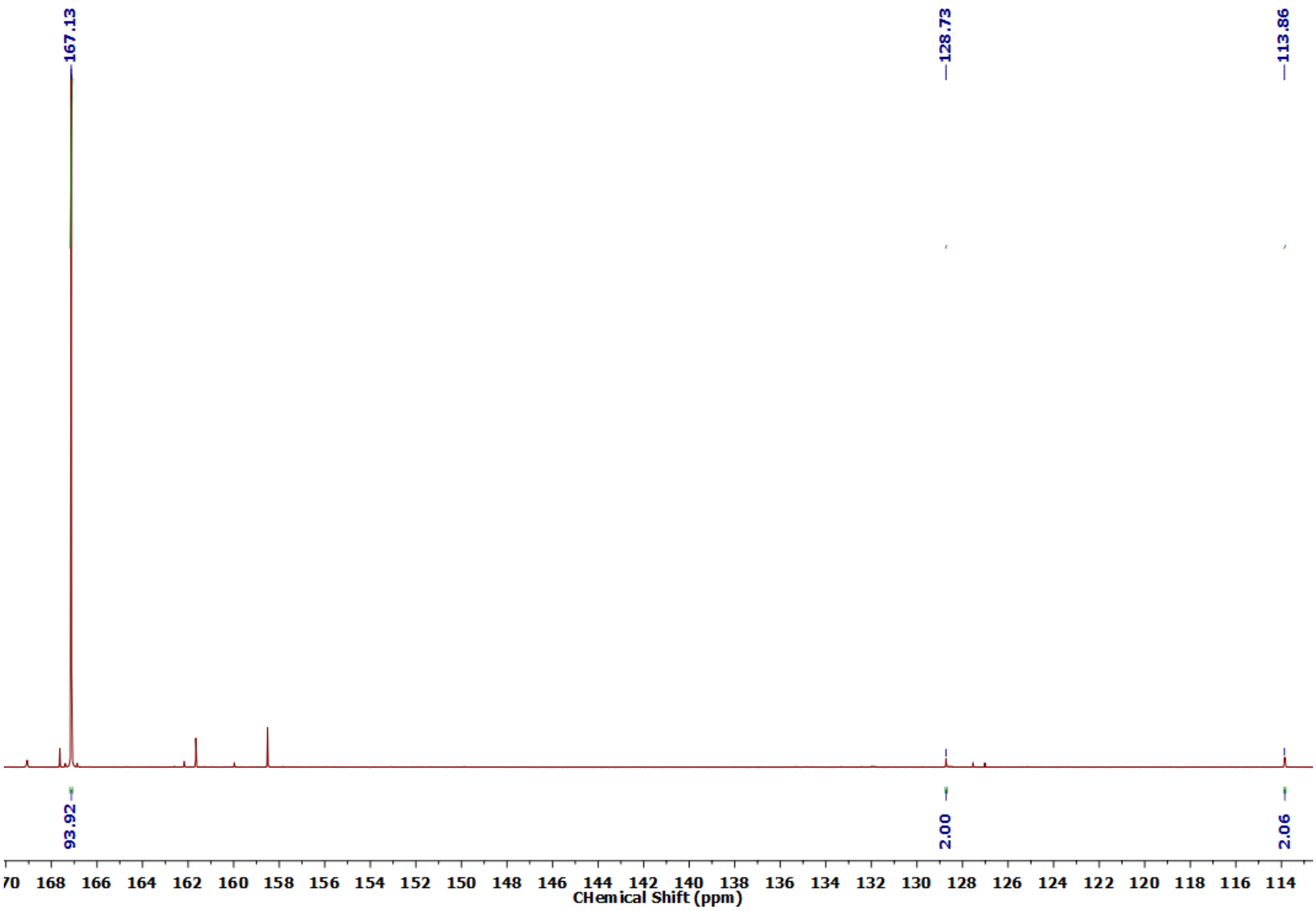

Figure S7. Isotopic labelling using $^{12}CO_2$ or $^{13}CO_2$ with analysis of the crude benzamide (**3**) product by ^{13}C (125 MHz) NMR spectroscopy.

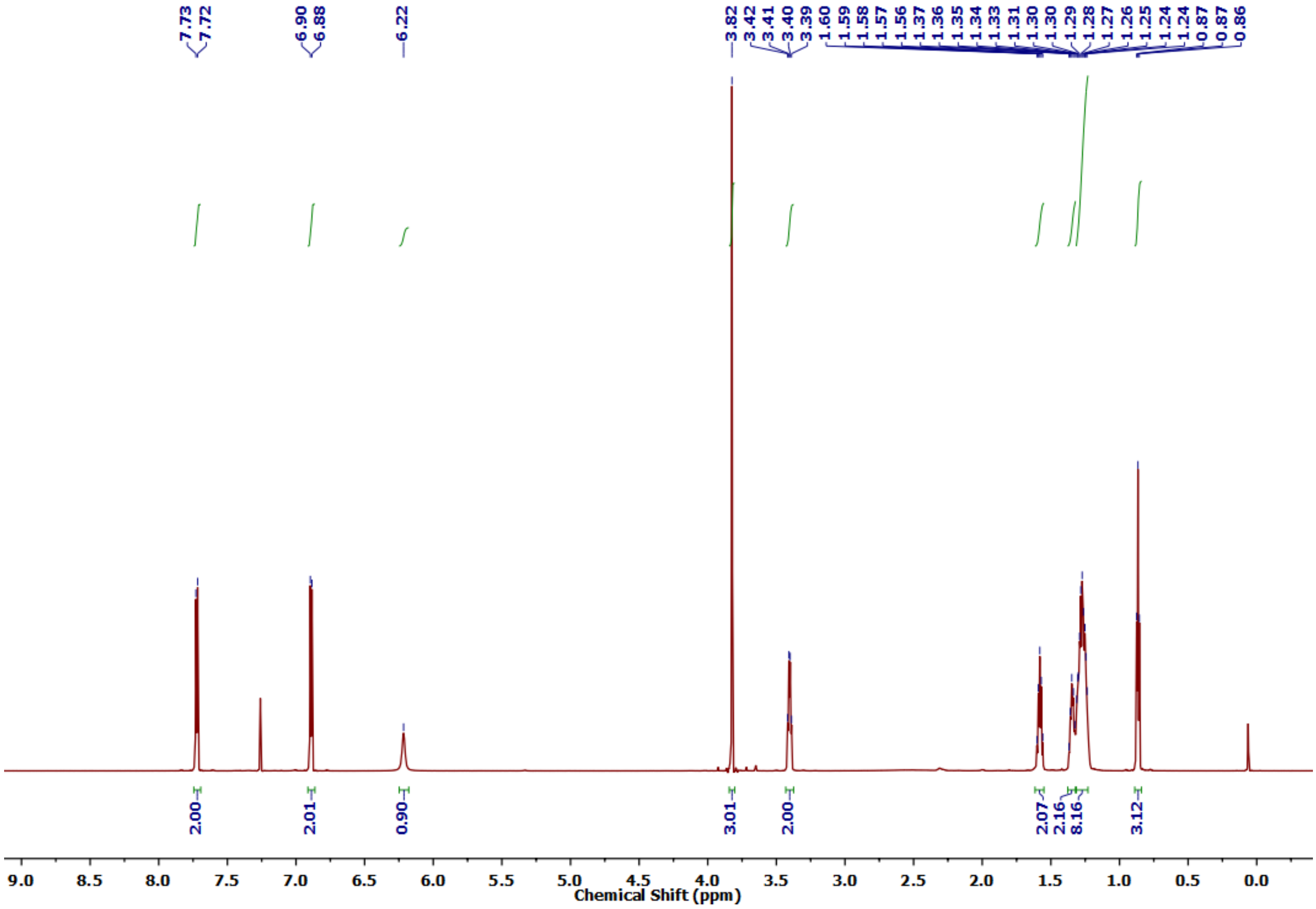

Figure S8. ^{1}H NMR analysis of **(3)** in $CDCl_3$ in 700 MHz.

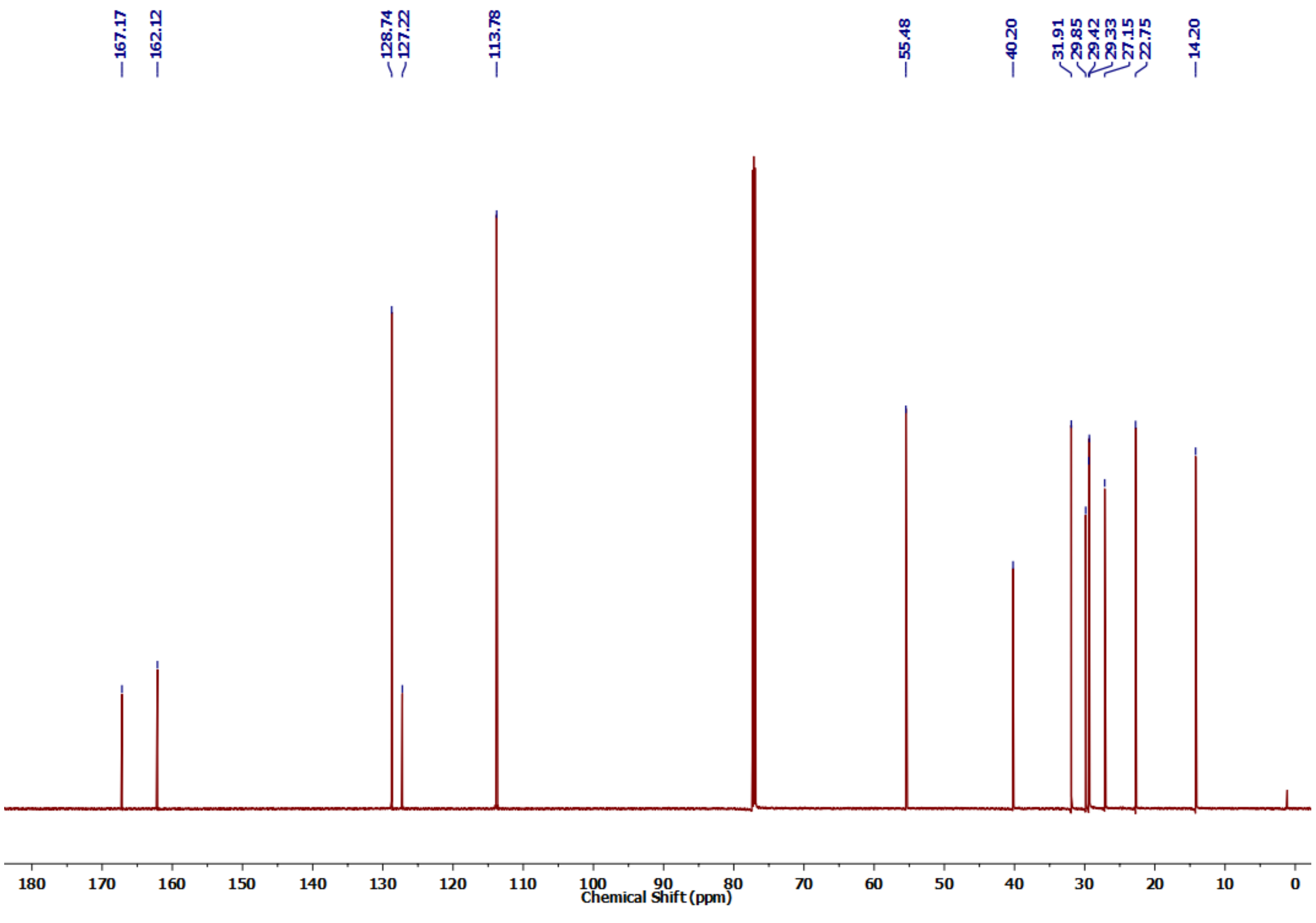

Figure S9. ^{13}C NMR analysis of **(3)** in $CDCl_3$ in 700 MHz.

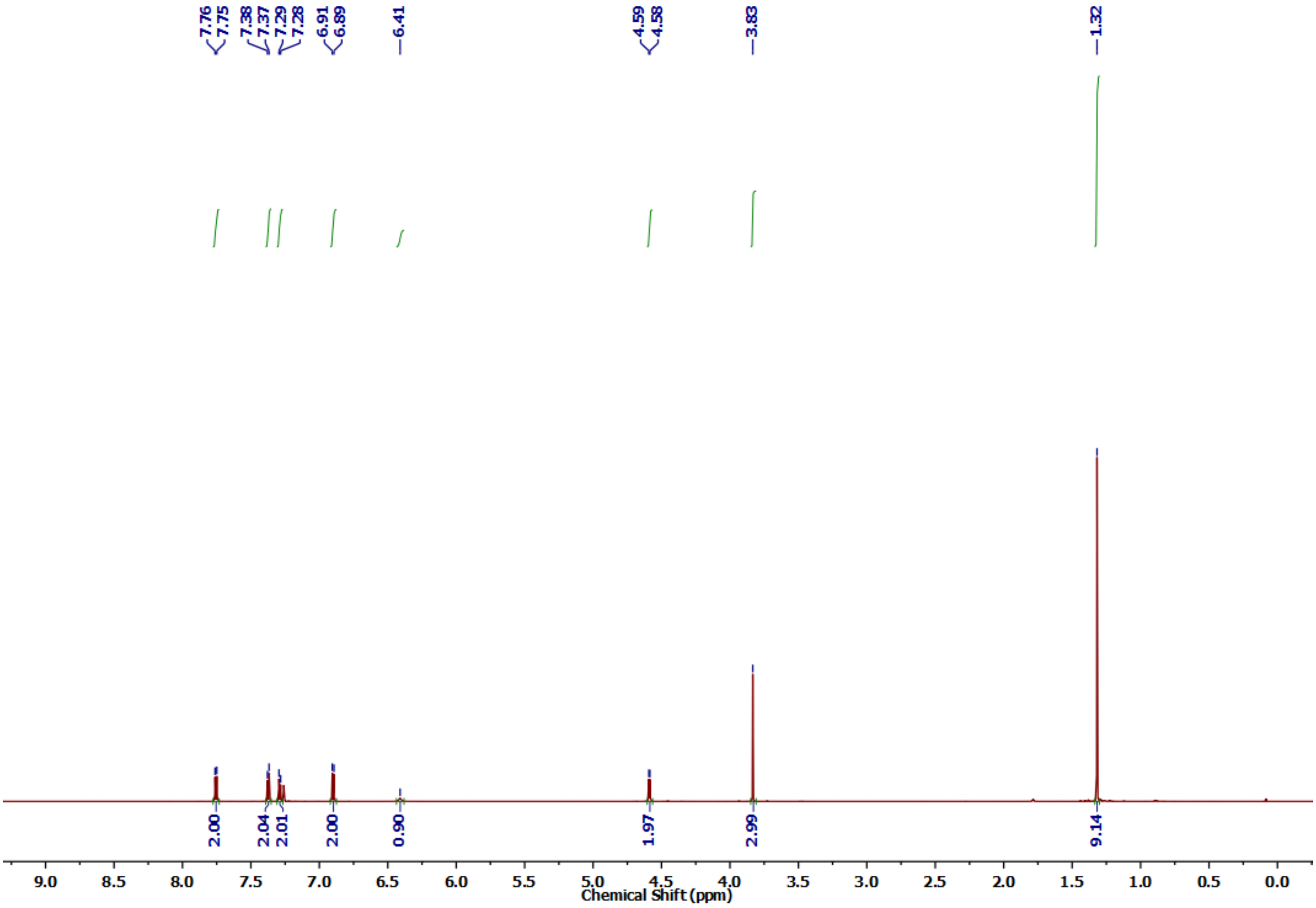

Figure S10. ^{1}H NMR analysis of **(4)** in $CDCl_3$ in 700 MHz.

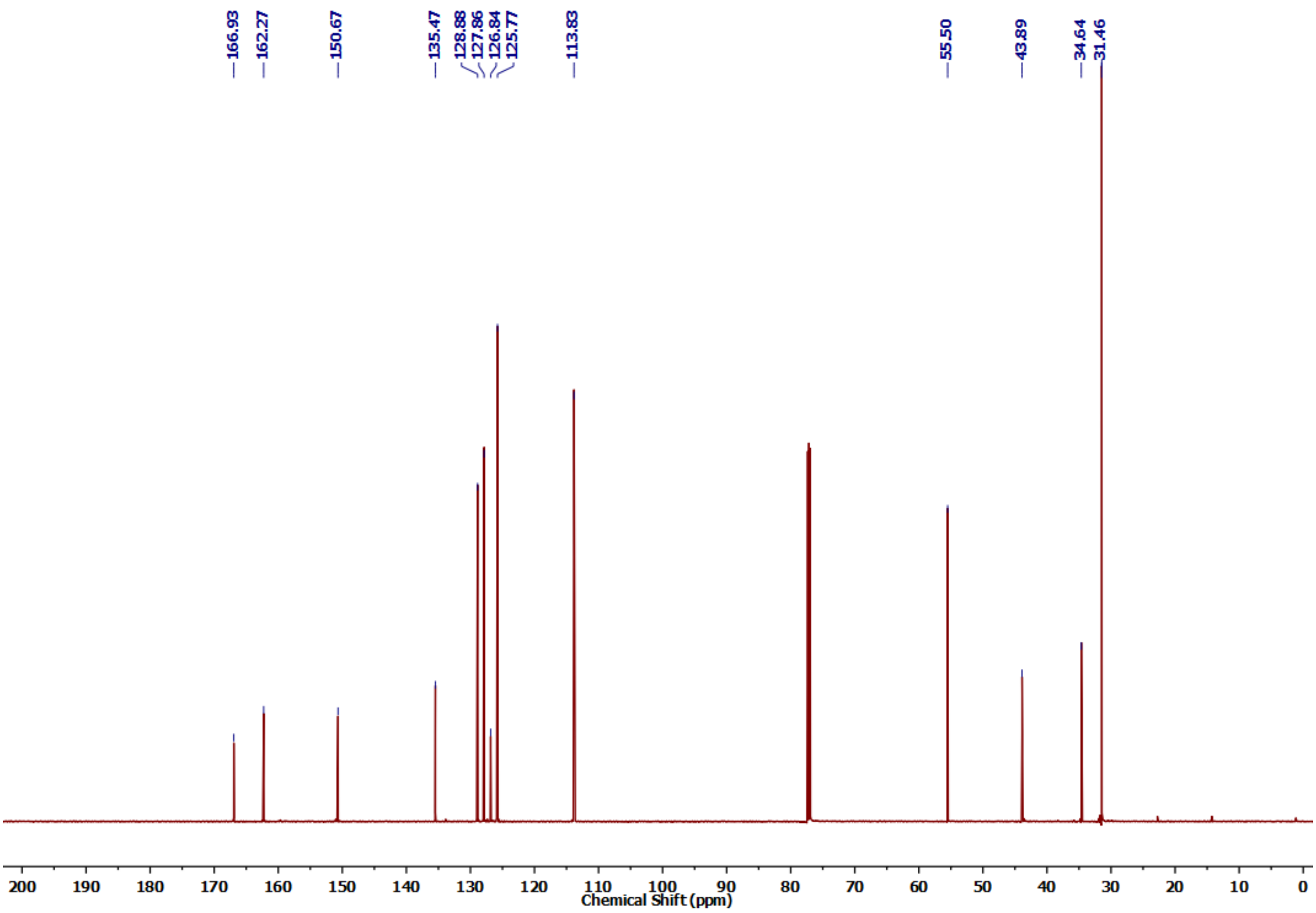

Figure S11. ^{13}C NMR analysis of **(4)** in $CDCl_3$ in 700 MHz.

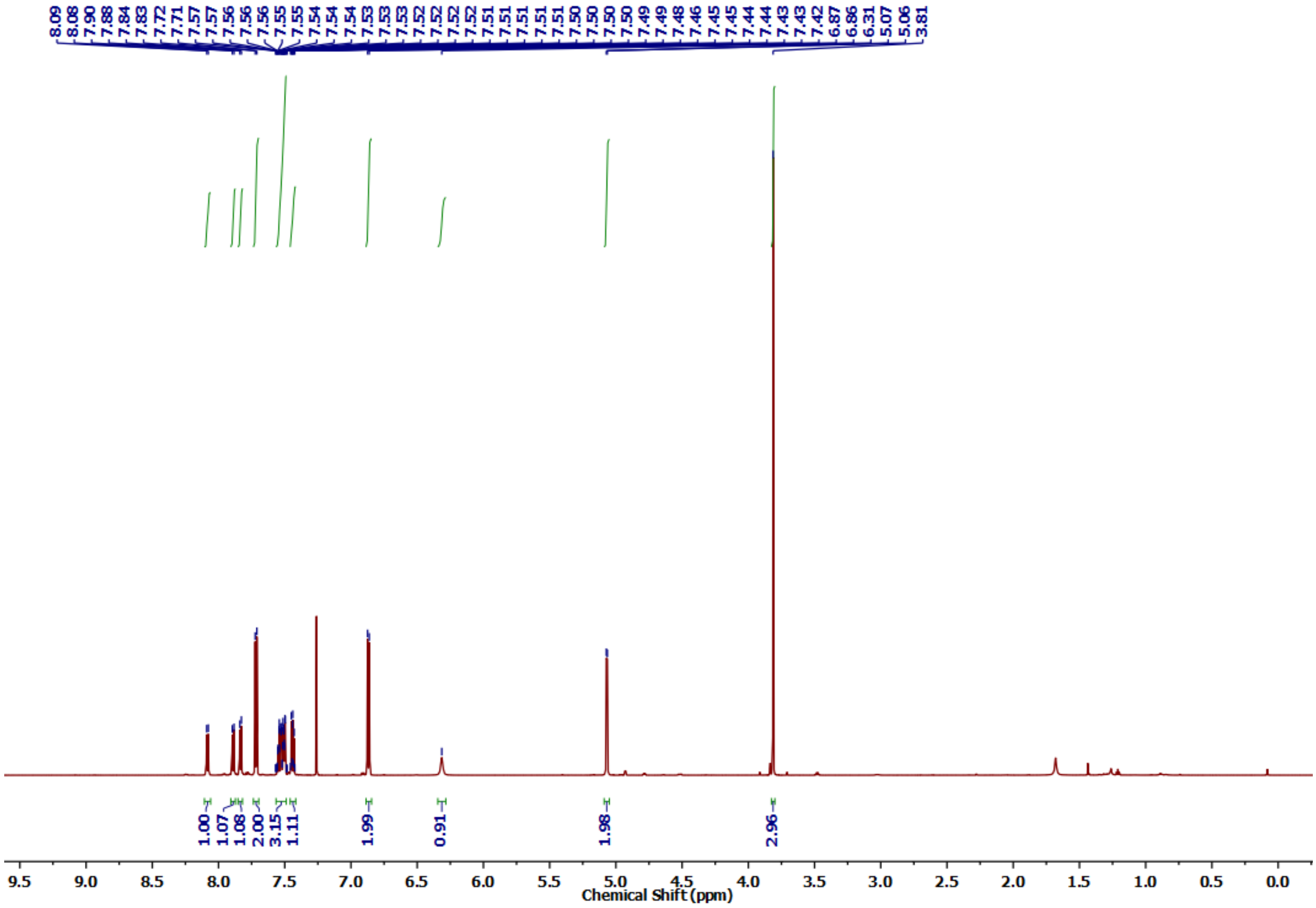

Figure S12. ^{1}H NMR analysis of **(5)** in $CDCl_3$ in 700 MHz.

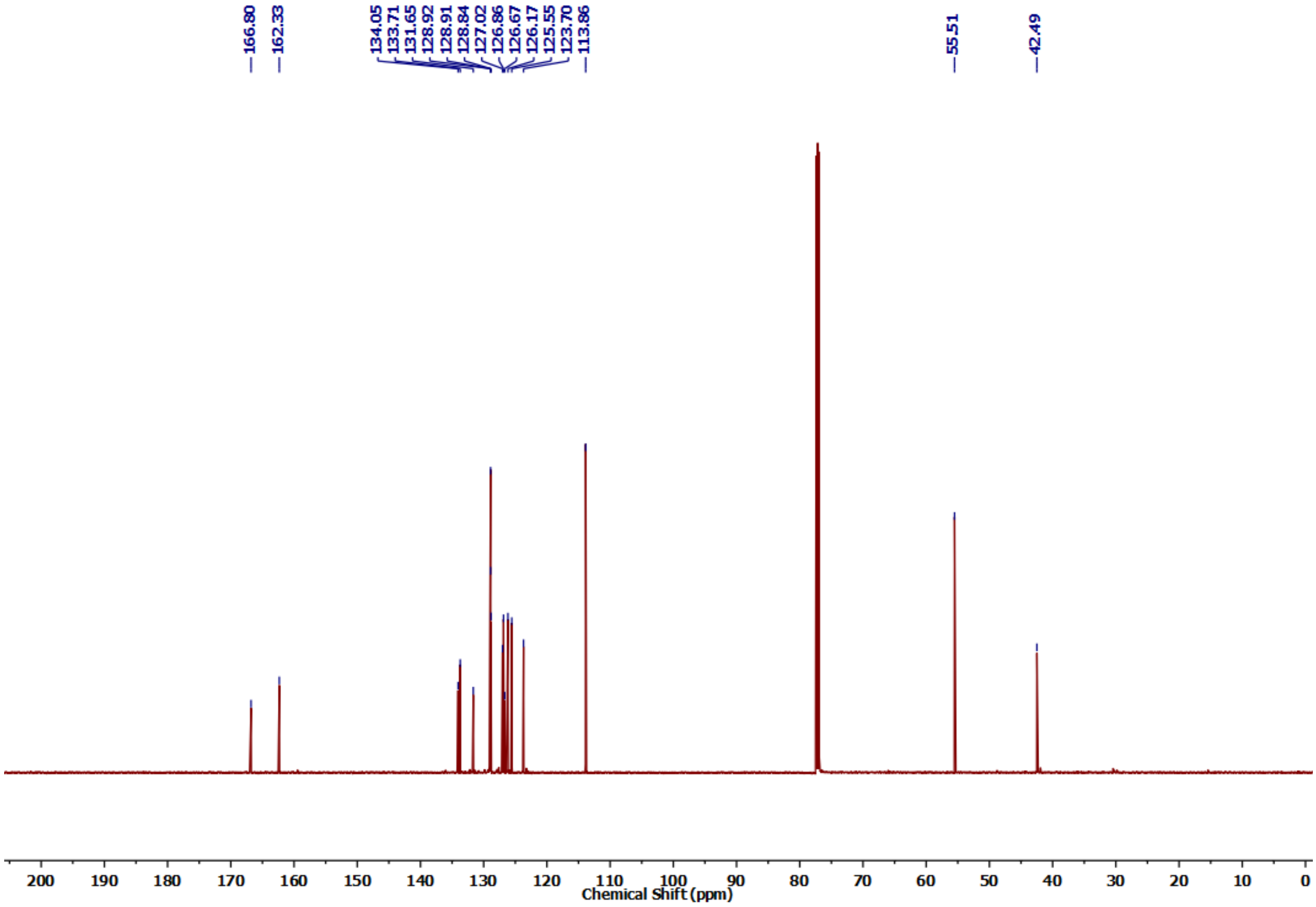

Figure S13. ^{13}C NMR analysis of **(5)** in $CDCl_3$ in 700 MHz.

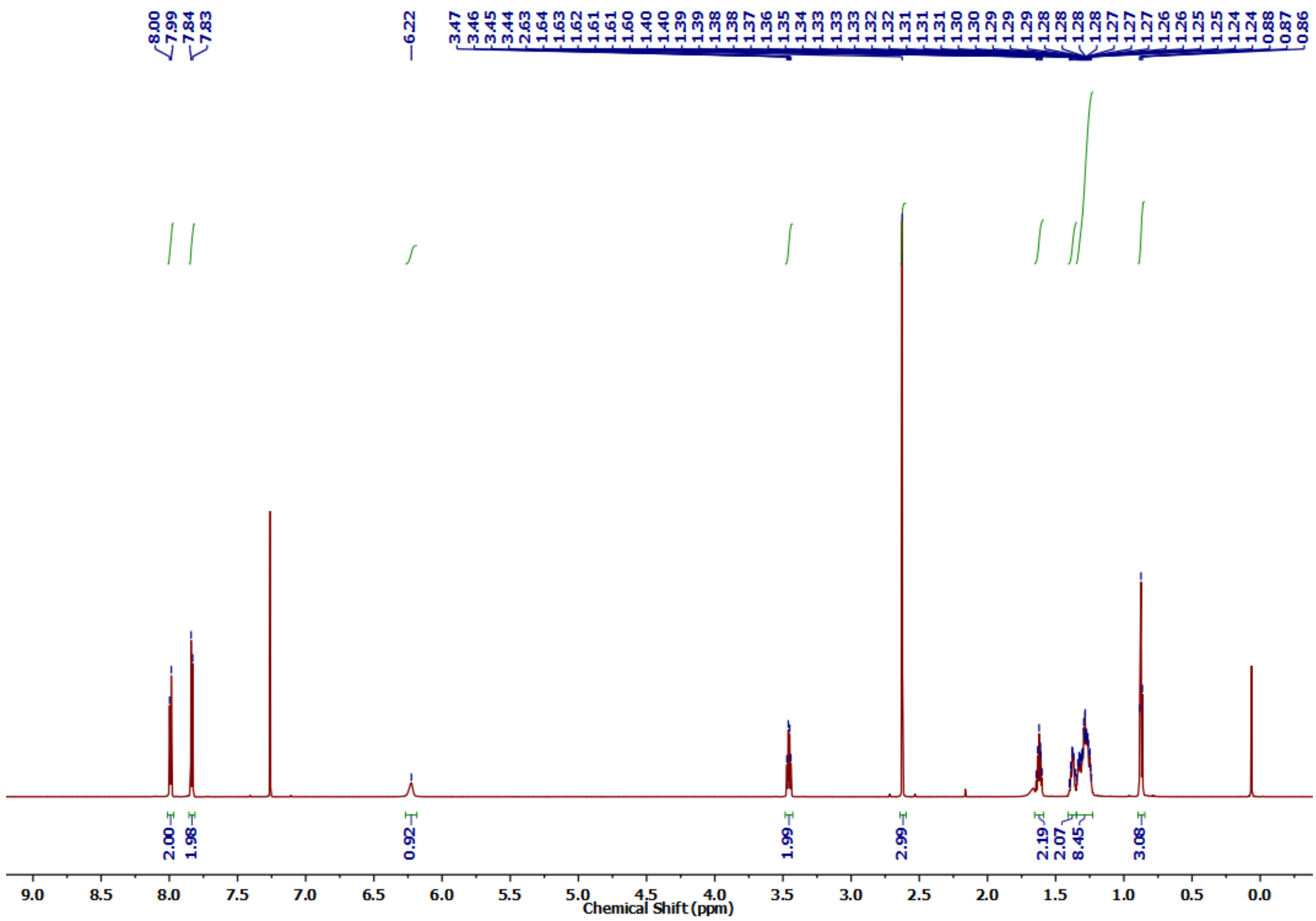

Figure S14. ^{1}H NMR analysis of **(8)** in $CDCl_3$ in 700 MHz.

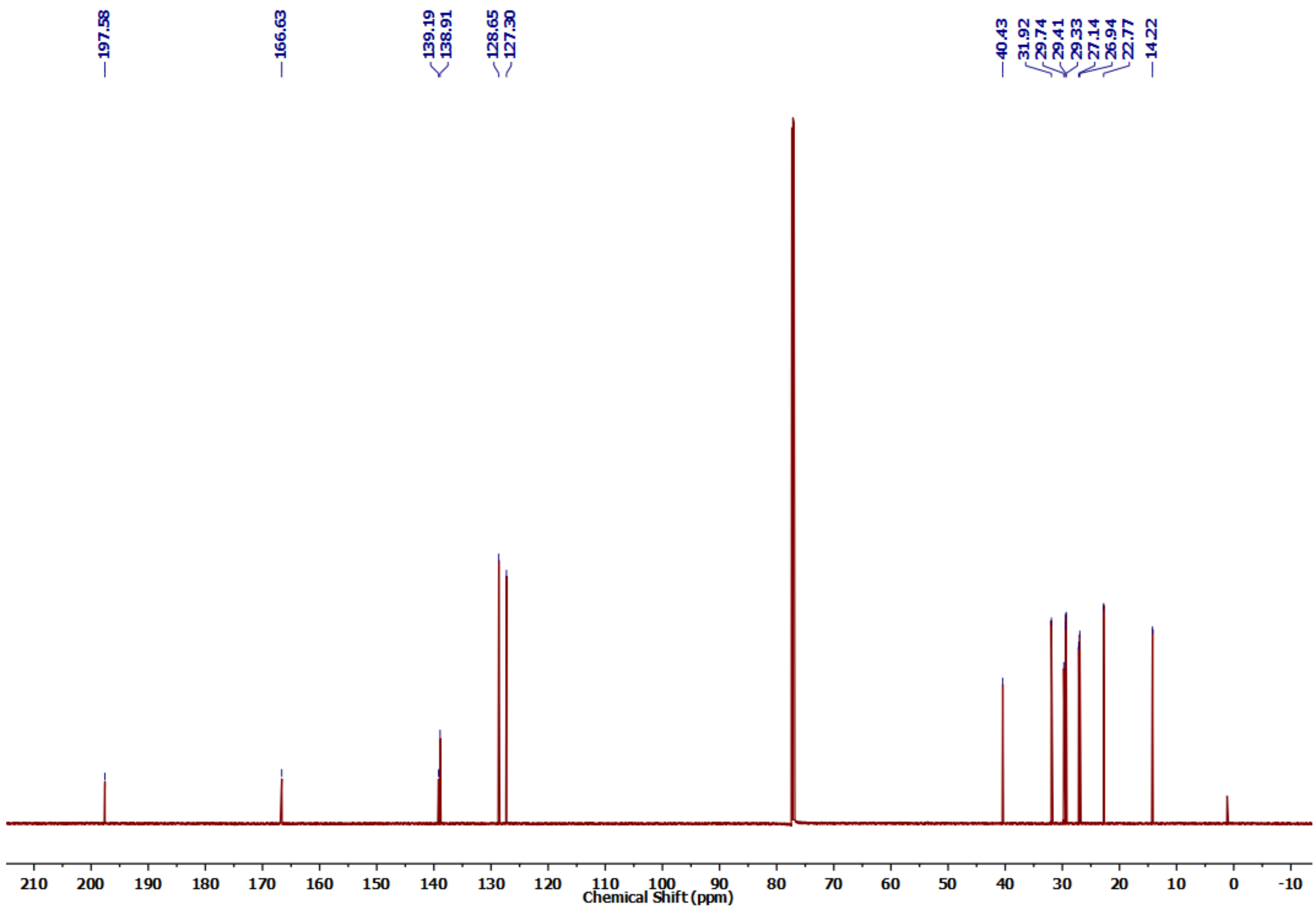

Figure S15. ^{13}C NMR analysis of **(8)** in $CDCl_3$ in 700 MHz.

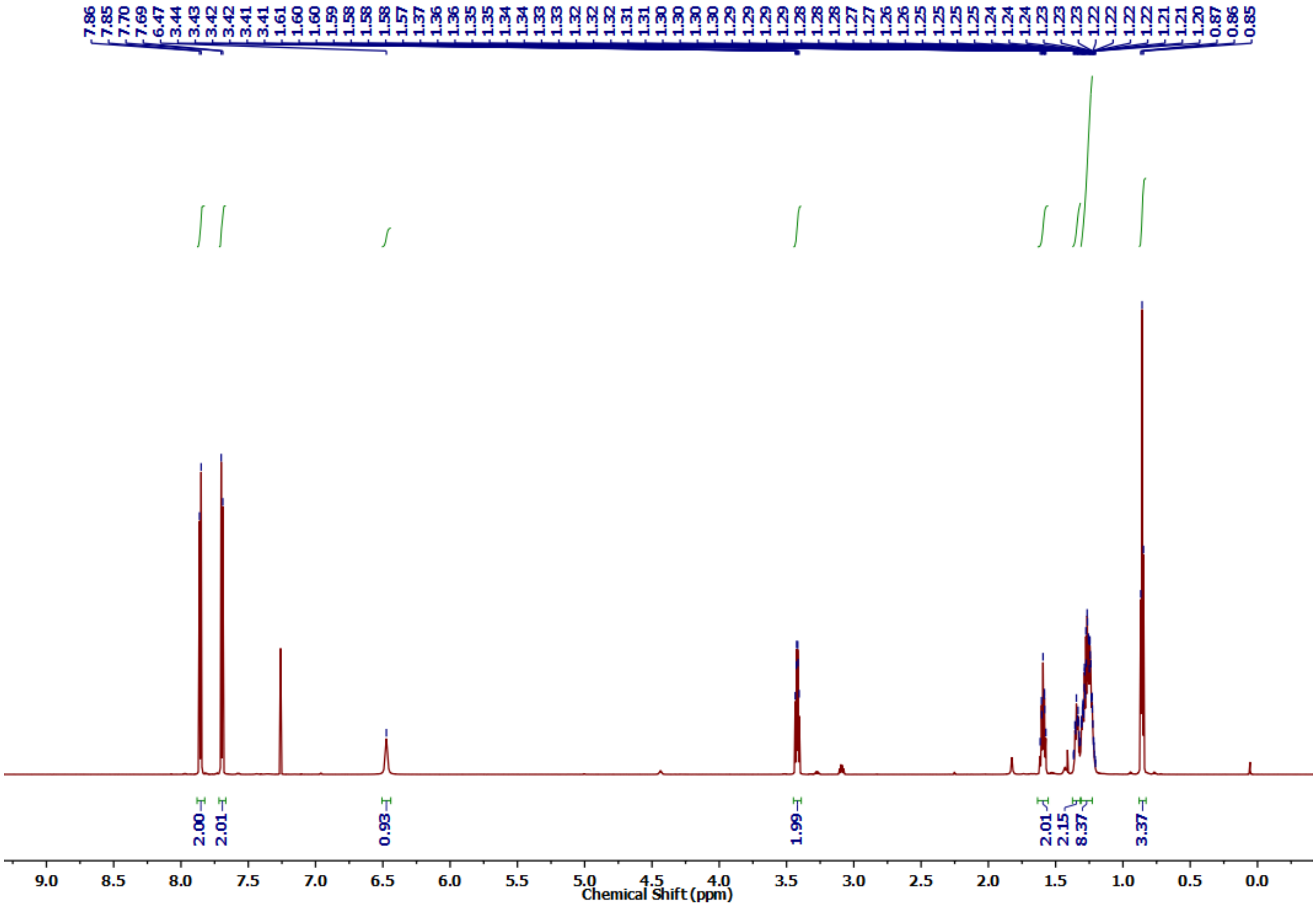

Figure S16. ^{1}H NMR analysis of **(9)** in $CDCl_3$ in 700 MHz.

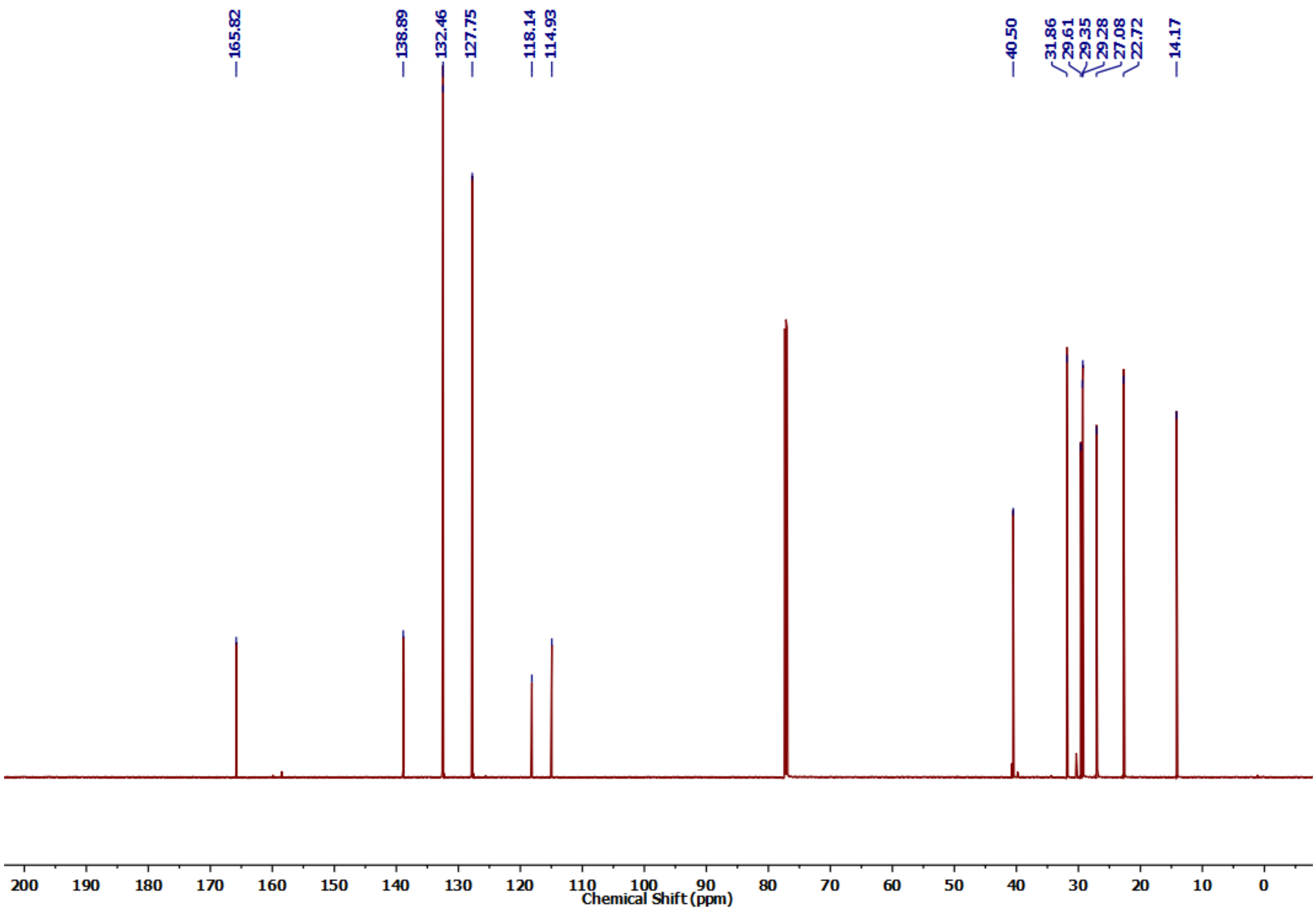

Figure S17. ^{13}C NMR analysis of **(9)** in $CDCl_3$ in 700 MHz.

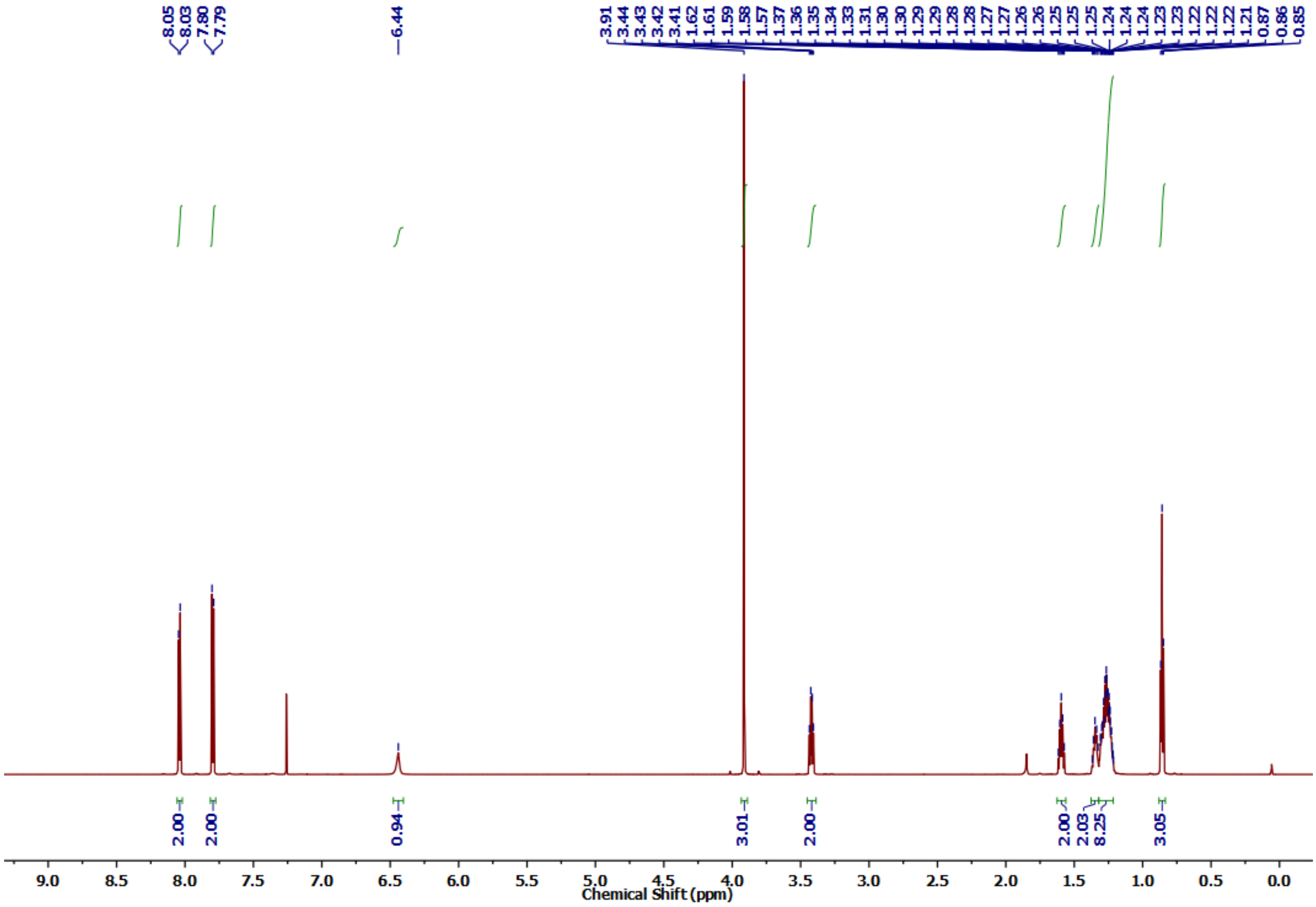

Figure S18. ^{1}H NMR analysis of **(10)** in $CDCl_3$ in 700 MHz.

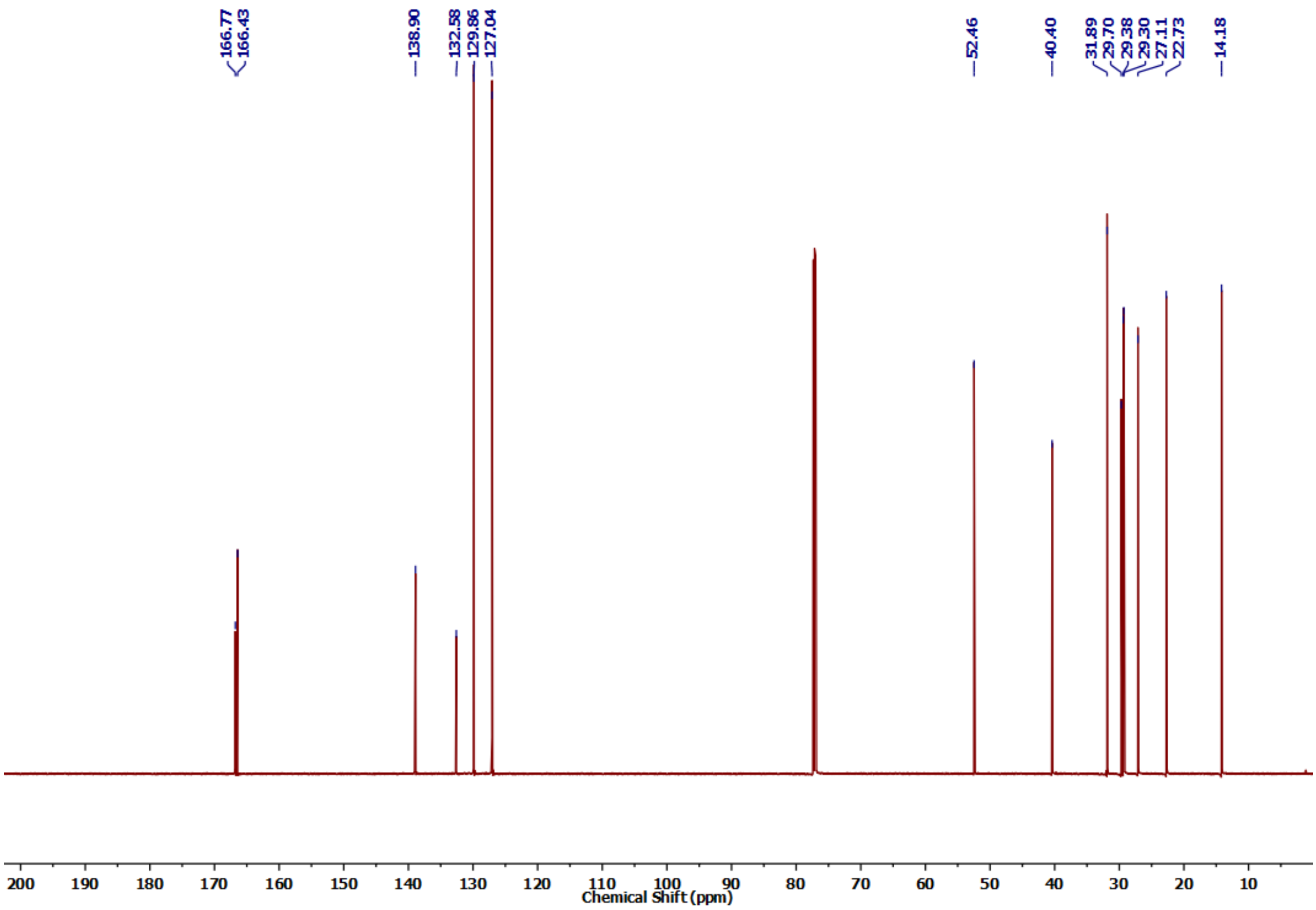

Figure S19. ^{13}C NMR analysis of **(10)** in $CDCl_3$ in 700 MHz.

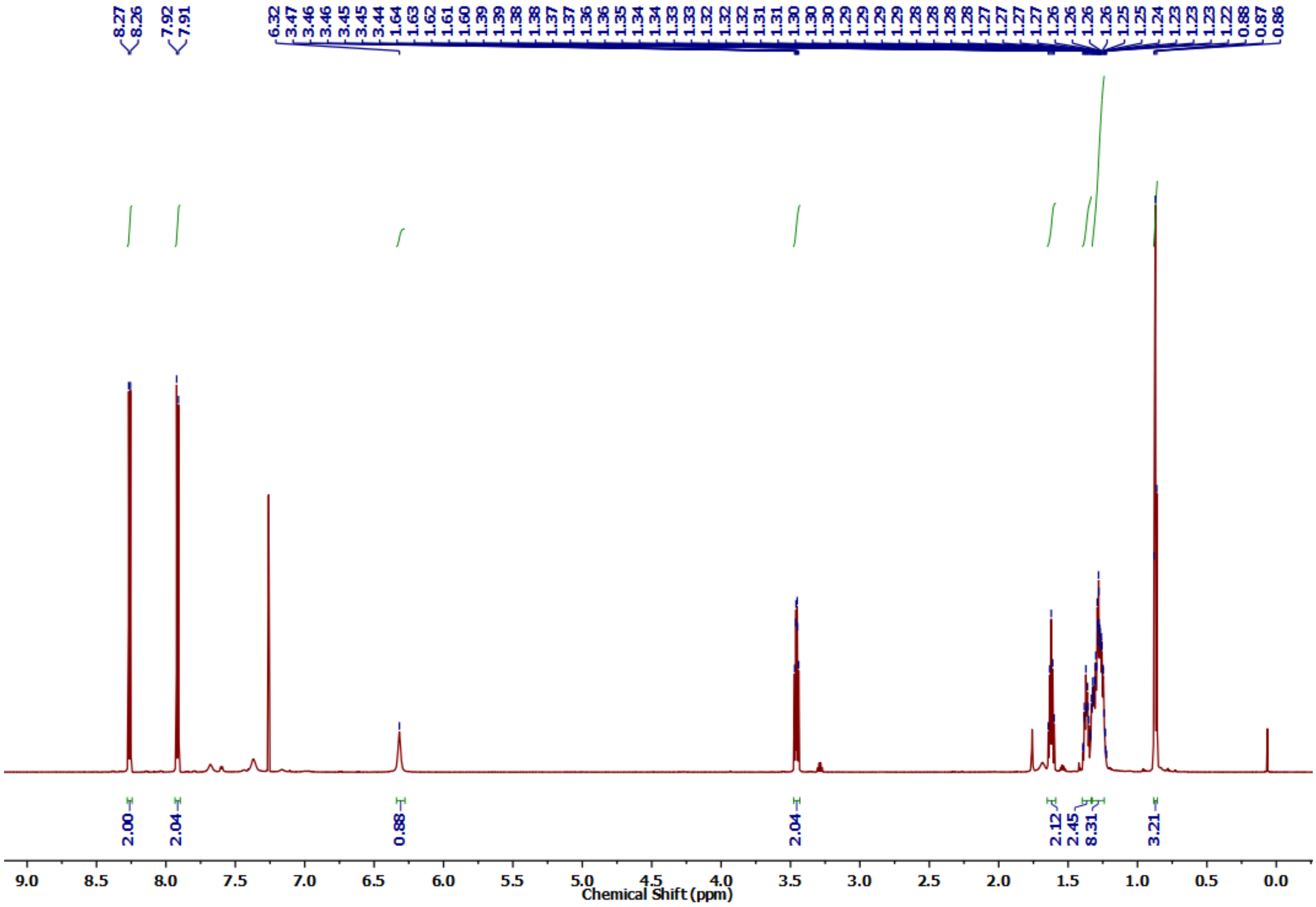

Figure S20. ^{1}H NMR analysis of **(11)** in $CDCl_3$ in 700 MHz.

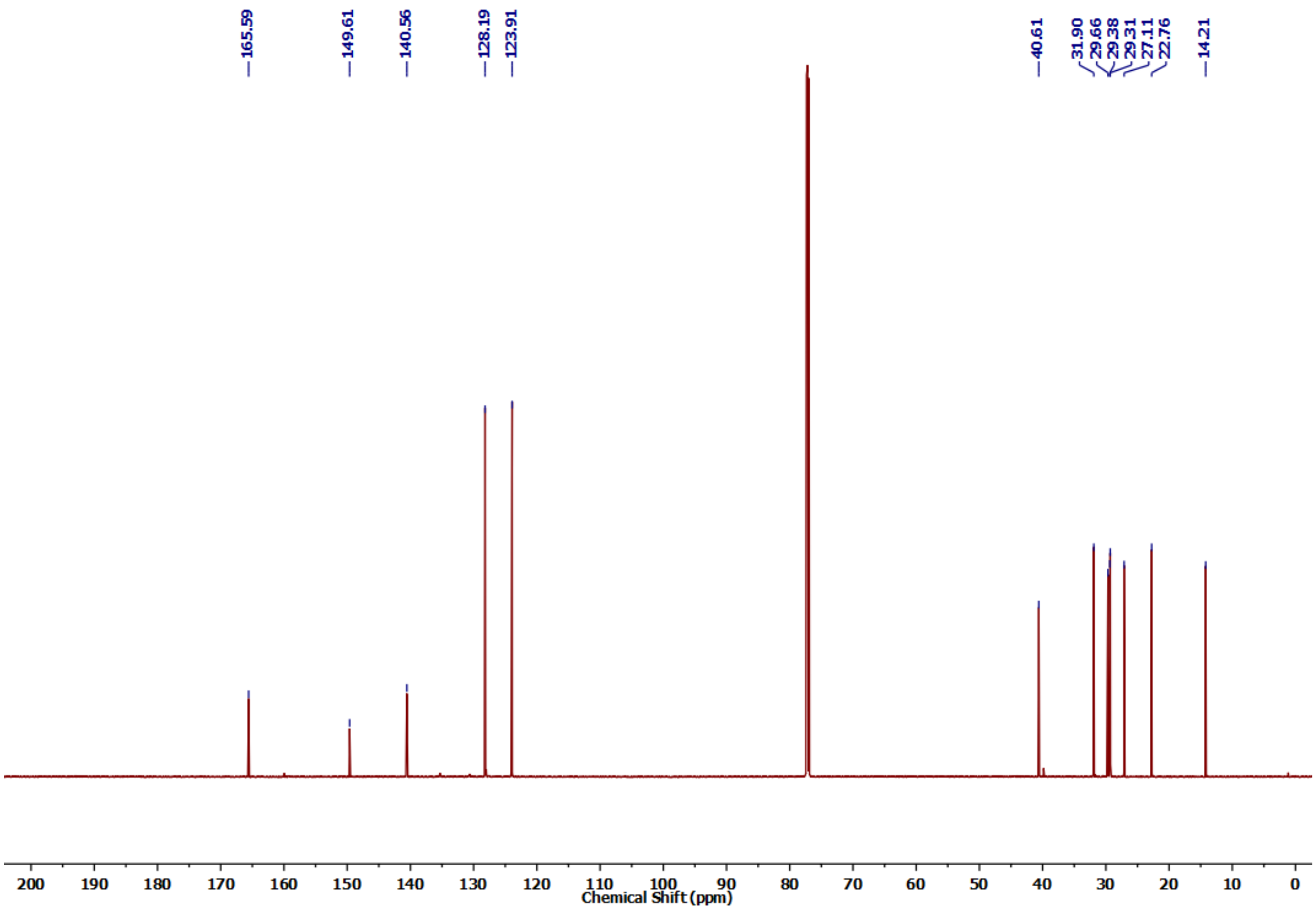

Figure S21. ^{13}C NMR analysis of **(11)** in $CDCl_3$ in 700 MHz.

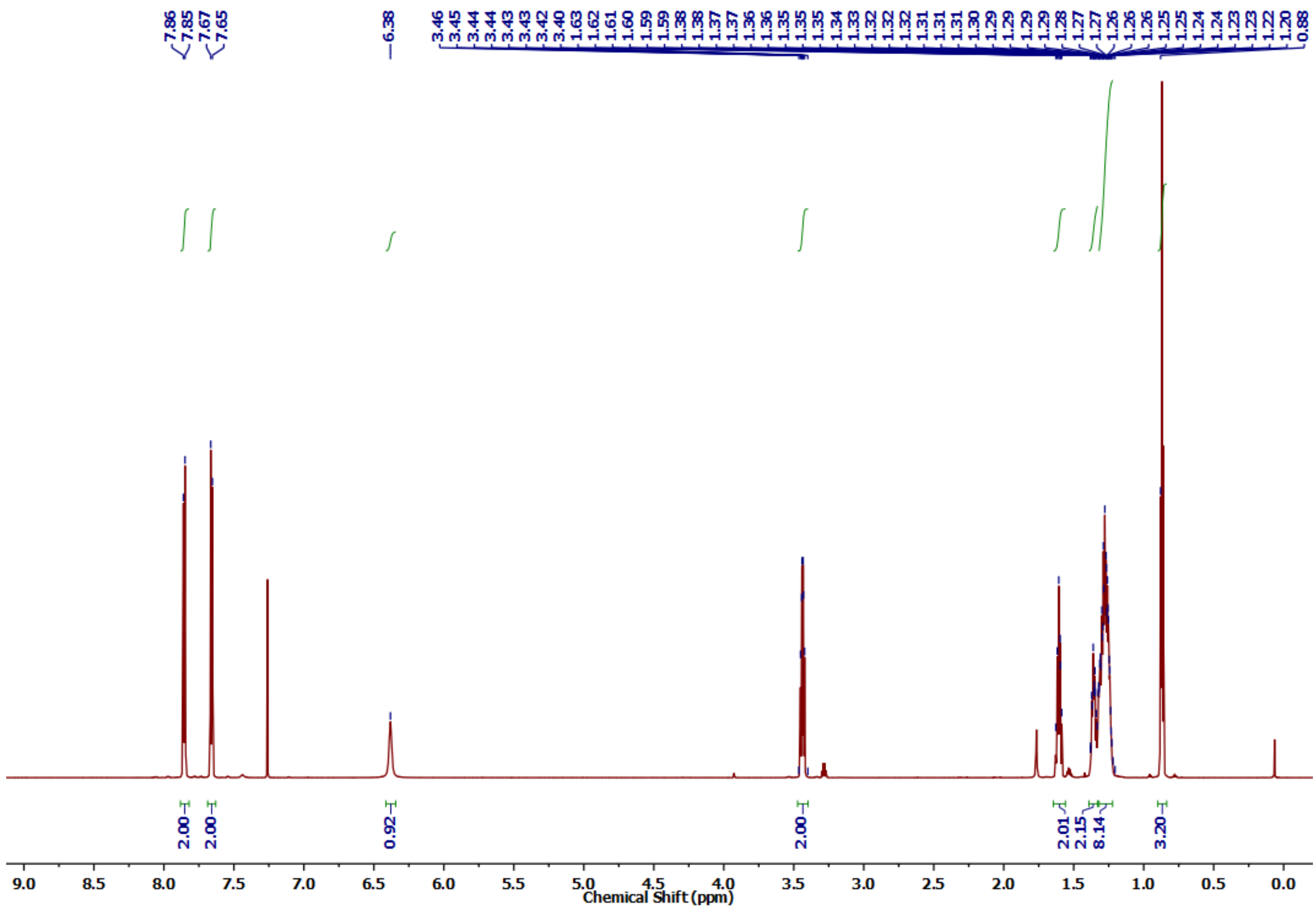

Figure S22. ^{1}H NMR analysis of **(12)** in $CDCl_3$ in 700 MHz.

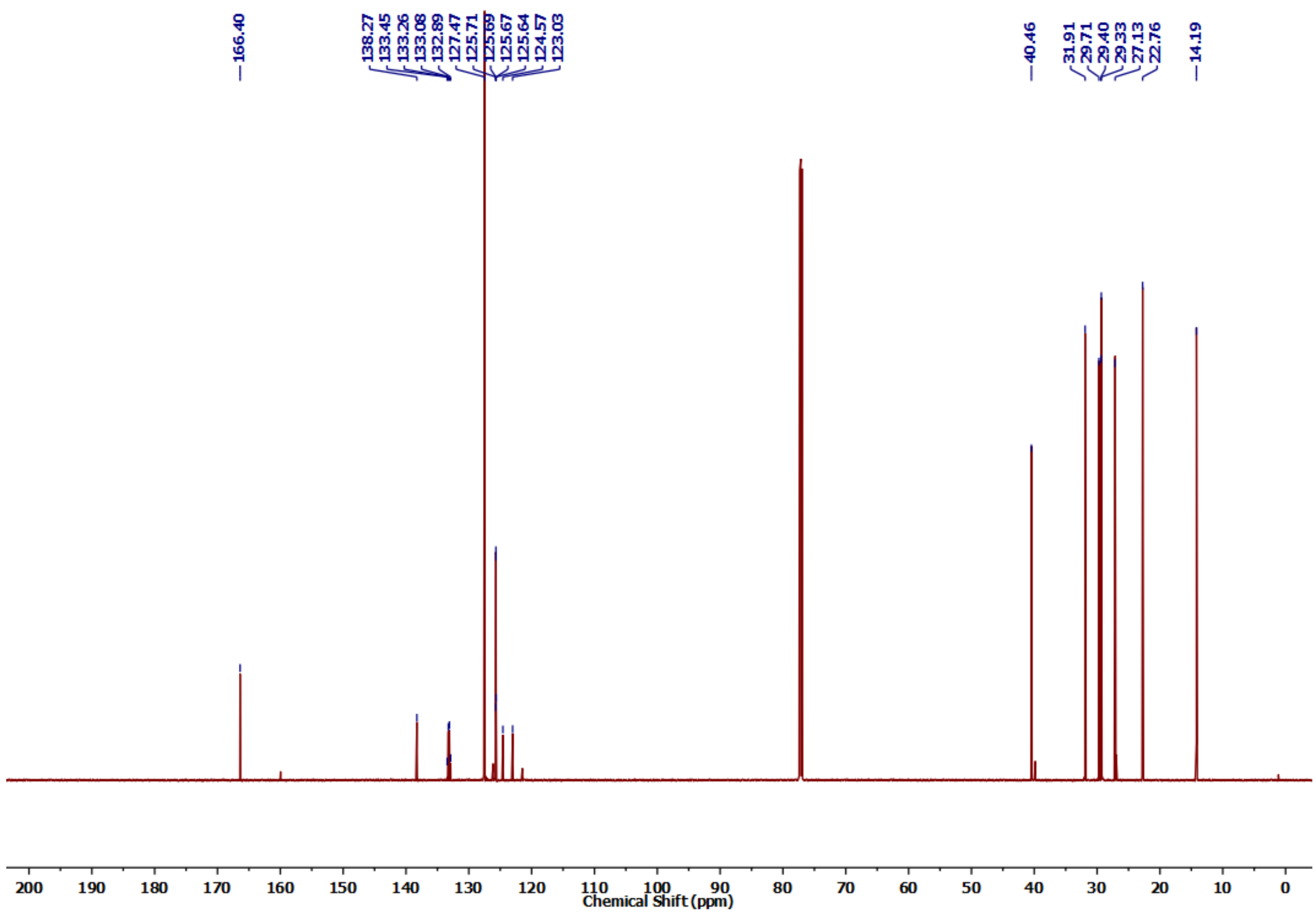

Figure S23. ^{13}C NMR analysis of **(12)** in $CDCl_3$ in 700 MHz.

Molecular Characterisation of New Compounds (MS)

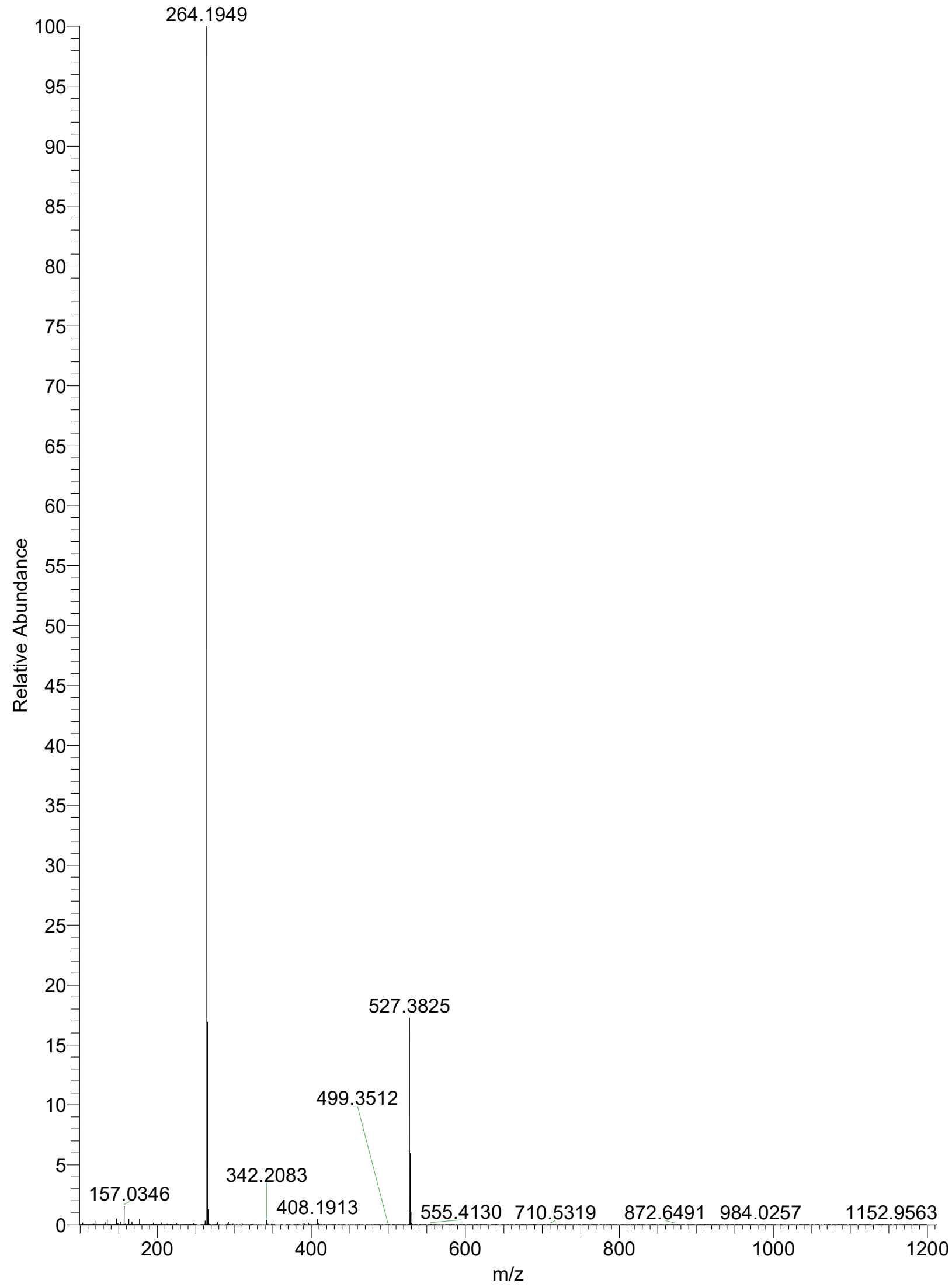

Figure S24. LC-MS analysis of **(3)** in MeOH.

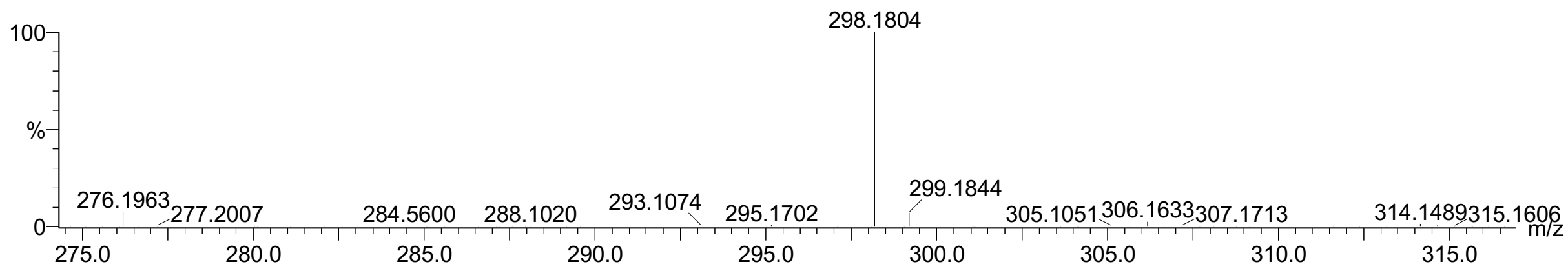

Minimum:				-1.5				
Maximum:		5.0	1000.0	50.0				
Mass	Calc. Mass	mDa	PPM	DBE	i-FIT	Norm	Conf(%)	Formula
298.1804	298.1807	-0.3	-1.0	8.5	42.1	n/a	n/a	C19 H24 N O2

Figure S25. LC-MS analysis of **(4)** in MeOH.

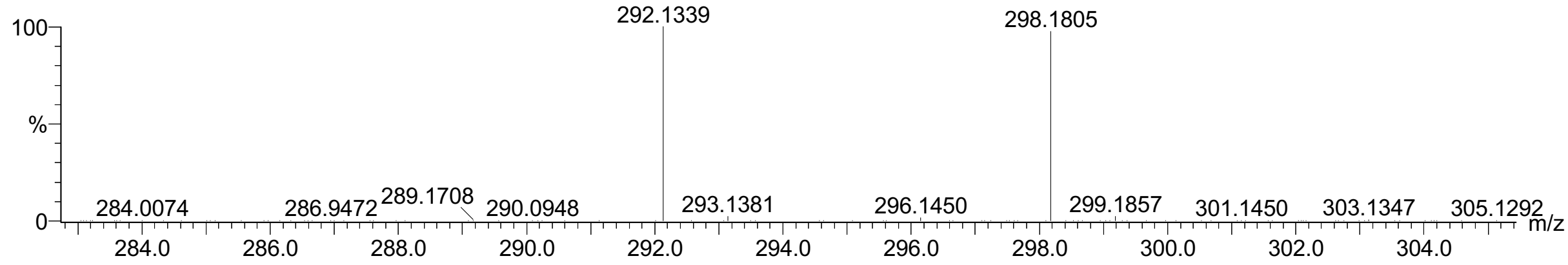

Minimum:				-1.5				
Maximum:		5.0	1000.0	50.0				
Mass	Calc. Mass	mDa	PPM	DBE	i-FIT	Norm	Conf(%)	Formula
292.1339	292.1338	0.1	0.3	11.5	87.9	n/a	n/a	C19 H18 N O2

Figure S26. LC-MS analysis of **(5)** in MeOH.

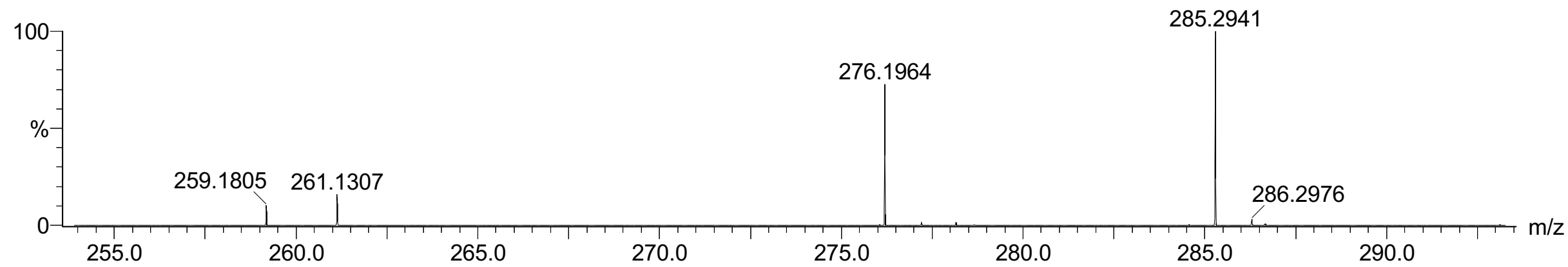

Minimum:				-1.5	
Maximum:		5.0	1000.0	50.0	
Mass	Calc. Mass	mDa	PPM	DBE	Formula
276.1964	276.1964	0.0	0.0	5.5	C17 H26 N O2

Figure S27. LC-MS analysis of **(8)** in MeOH.

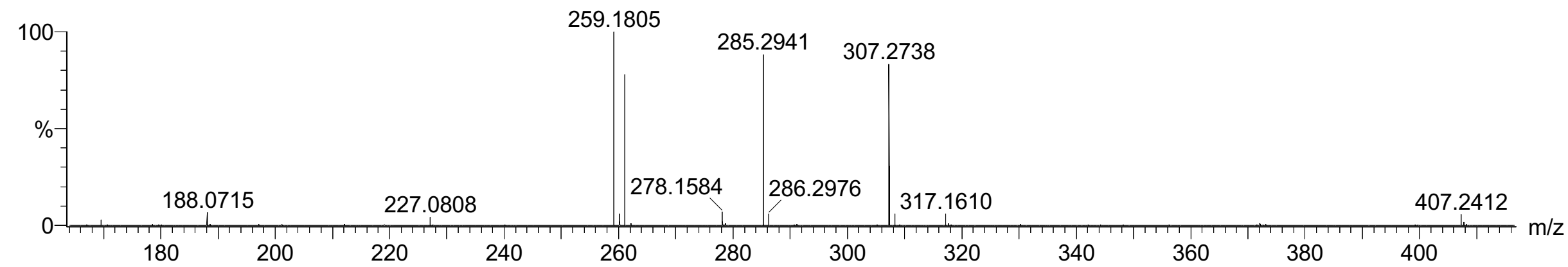

Minimum:				-1.5	
Maximum:		5.0	1000.0	50.0	
Mass	Calc. Mass	mDa	PPM	DBE	Formula
259.1805	259.1810	-0.5	-1.9	6.5	C16 H23 N2 O

Figure S28. LC-MS analysis of **(9)** in MeOH.

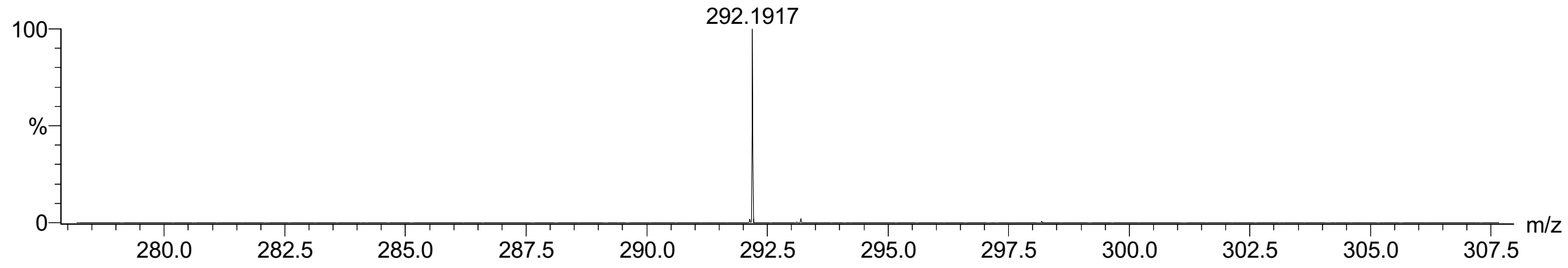

	Mass	Calc. Mass	mDa	PPM	DBE	Formula
Minimum:					-1.5	
Maximum:			5.0	1000.0	50.0	
	292.1917	292.1913	0.4	1.4	5.5	C17 H26 N O3

Figure S29. LC-MS analysis of **(10)** in MeOH.

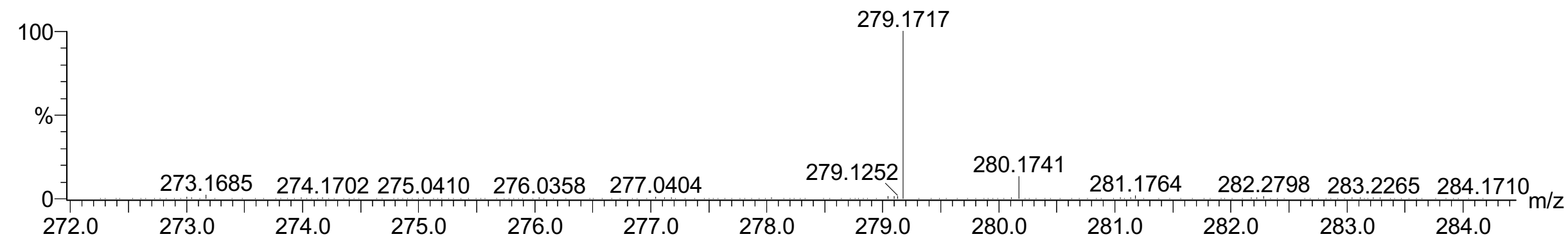

	Mass	Calc. Mass	mDa	PPM	DBE	i-FIT	Norm	Conf(%)	Formula
Minimum:					-1.5				
Maximum:			5.0	1200.0	50.0				
	279.1717	279.1709	0.8	2.9	5.5	348.7	n/a	n/a	C15 H23 N2 O3

Figure S30. LC-MS analysis of **(11)** in MeOH.

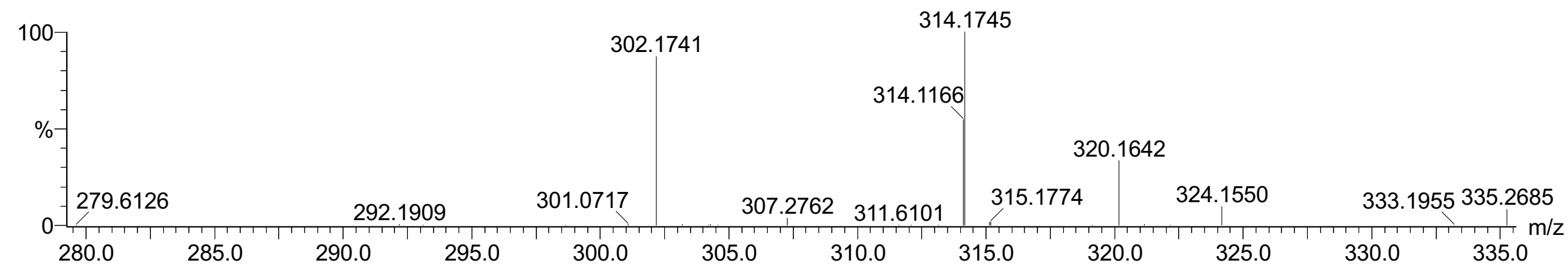

	Mass	Calc. Mass	mDa	PPM	DBE	i-FIT	Norm	Conf(%)	Formula
Minimum:					-1.5				
Maximum:			5.0	1000.0	50.0				
	302.1741	302.1732	0.9	3.0	4.5	39.4	n/a	n/a	C16 H23 N O F3

Figure S31. LC-MS analysis of **(12)** in MeOH.

References

[1] A. Call, M. Cibian, K. Yamamoto, T. Nakazono, K. Yamauchi, K. Sakai, "Highly Efficient and Selective Photocatalytic CO_2 Reduction to CO in Water by a Cobalt Porphyrin Molecular Catalyst" *ACS Catal.* **2019**, *9*, 4867–4874.

[2] A. Nakada, K. Koike, T. Nakashima, T. Morimoto, O. Ishitani, "Photocatalytic CO_2 Reduction to Formic Acid Using a Ru(II)–Re(I) Supramolecular Complex in an Aqueous Solution" *Inorg. Chem.* **2015**, *54*, 1800–1807.

[3] T. Nakazono, A. R. Parent, K. Sakai, "Cobalt porphyrins as homogeneous catalysts for water oxidation" *Chem. Commun.* **2013**, *49*, 6325–6327.

[4] X. Zhang, M. Cibian, A. Call, K. Yamauchi, K. Sakai, "Photochemical CO_2 Reduction Driven by Water-Soluble Copper(I) Photosensitizer with the Catalysis Accelerated by Multi-Electron Chargeable Cobalt Porphyrin" *ACS Catal.* **2019**, *9*, 11263–11273.

[5] S.-P. Luo, E. Mejía, A. Friedrich, A. Pazidis, H. Junge, A.-E. Surkus, R. Jackstell, S. Denurra, S. Gladiali, S. Lochbrunner, M. Beller, "Photocatalytic Water Reduction with Copper-Based Photosensitizers: A Noble-Metal-Free System" *Angew. Chem. Int. Ed.* **2013**, *52*, 419–423.

[6] M. Karnahl, E. Mejía, N. Rockstroh, S. Tschierlei, S.-P. Luo, K. Grabow, A. Kruth, V. Brüser, H. Junge, S. Lochbrunner, M. Beller, "Photocatalytic Hydrogen Production with Copper Photosensitizer–Titanium Dioxide Composites" *ChemCatChem* **2014**, *6*, 82–86.

[7] A. Call, C. Casadevall, F. Acuña-Parés, A. Casitas, J. Lloret-Fillol, "Dual cobalt–copper light-driven catalytic reduction of aldehydes and aromatic ketones in aqueous media" *Chem. Sci.* **2017**, *8*, 4739–4749.

[8] C. Casadevall, J. Aragón, S. Cañellas, M. A. Pericàs, J. Lloret-Fillol, X. Caldentey in *Power High-Throughput Exp. Gen. Top. Enabling Technol. Synth. Catal. Vol. 1*, American Chemical Society, **2022**, pp. 145–165.

[9] D. Sah, Surabhi, P. Gupta, J. Shabir, M. Dhama, S. Mozumdar, "Diamine-functionalized porous graphene oxide sheets decorated with palladium oxide nanoparticles for the oxidative amidation of aldehydes" *New J. Chem.* **2022**, *46*, 20828–20843.

[10] T. Ben Halima, J. K. Vandavasi, M. Shkoor, S. G. Newman, "A Cross-Coupling Approach to Amide Bond Formation from Esters" *ACS Catal.* **2017**, *7*, 2176–2180.

[11] M. K. Gupta, A. Panda, N. K. Sharma, "Ru(II)-Catalyzed C(sp^2)–H Activation Annulation: Synthesis of Fluorescent Benzoisoquinolonyl Acetate/Peptides from N-Arylamides and Ethyne at Room Temperature" *J. Org. Chem.* **2024**, *89*, 7485–7494.

[12] A. R. Prosser, J. E. Banning, M. Rubina, M. Rubin, "Formal Nucleophilic Substitution of Bromocyclopropanes with Amides en route to Conformationally Constrained β-Amino Acid Derivatives" *Org. Lett.* **2010**, *12*, 3968–3971.

[13] R. E. Islas, J. Cárdenas, R. Gaviño, E. García-Ríos, L. Lomas-Romero, J. A. Morales-Serna, "Phosphinito palladium(II) complexes as catalysts for the synthesis of 1,3-enynes, aromatic alkynes and ynones" *RSC Adv.* **2017**, *7*, 9780–9789.

[14] B. Yu, H. Sun, Z. Xie, G. Zhang, L.-W. Xu, W. Zhang, Z. Gao, "Privilege Ynone Synthesis via Palladium-Catalyzed Alkynylation of 'Super-Active Esters'" *Org. Lett.* **2015**, *17*, 3298–3301.

[15] E. Wilhelm, R. Battino, "Thermodynamic functions of the solubilities of gases in liquids at 25.deg." *Chem. Rev.* **1973**, *73*, 1–9.

[16] J. R. Martinelli, D. A. Watson, D. M. M. Freckmann, T. E. Barder, S. L. Buchwald, "Palladium-Catalyzed Carbonylation Reactions of Aryl Bromides at Atmospheric Pressure: A General System Based on Xantphos" *J. Org. Chem.* **2008**, *73*, 7102–7107.

[17] K. M. Bjerglund, T. Skrydstrup, G. A. Molander, "Carbonylative Suzuki Couplings of Aryl Bromides with Boronic Acid Derivatives under Base-Free Conditions" *Org. Lett.* **2014**, *16*, 1888–1891.

[18] P. Hermange, A. T. Lindhardt, R. H. Taaning, K. Bjerglund, D. Lupp, T. Skrydstrup, "Ex Situ Generation of Stoichiometric and Substoichiometric ^{12}CO and ^{13}CO and Its Efficient Incorporation in Palladium Catalyzed Aminocarbonylations" *J. Am. Chem. Soc.* **2011**, *133*, 6061–6071.
